# Structures, functions and adaptations of the human LINE-1 ORF2 protein

**DOI:** 10.1038/s41586-023-06947-z

**Published:** 2023-12-14

**Authors:** Eric T. Baldwin, Trevor van Eeuwen, David Hoyos, Arthur Zalevsky, Egor P. Tchesnokov, Roberto Sánchez, Bryant D. Miller, Luciano H. Di Stefano, Francesc Xavier Ruiz, Matthew Hancock, Esin Işik, Carlos Mendez-Dorantes, Thomas Walpole, Charles Nichols, Paul Wan, Kirsi Riento, Rowan Halls-Kass, Martin Augustin, Alfred Lammens, Anja Jestel, Paula Upla, Kera Xibinaku, Samantha Congreve, Maximiliaan Hennink, Kacper B. Rogala, Anna M. Schneider, Jennifer E. Fairman, Shawn M. Christensen, Brian Desrosiers, Gregory S. Bisacchi, Oliver L. Saunders, Nafeeza Hafeez, Wenyan Miao, Rosana Kapeller, Dennis M. Zaller, Andrej Sali, Oliver Weichenrieder, Kathleen H. Burns, Matthias Götte, Michael P. Rout, Eddy Arnold, Benjamin D. Greenbaum, Donna L. Romero, John LaCava, Martin S. Taylor

**Affiliations:** 1 ROME Therapeutics, Boston, MA USA; 2 Laboratory of Cellular and Structural Biology, The Rockefeller University, New York, NY USA; 3 Computational Oncology, Department of Epidemiology & Biostatistics, Memorial Sloan Kettering Cancer Center, New York, NY USA; 4 Department of Bioengineering and Therapeutic Sciences University of California, San Francisco, San Francisco, CA USA; 5 Department of Pharmaceutical Chemistry, University of California, San Francisco, San Francisco, CA USA; 6 Quantitative Biology Institute, University of California, San Francisco, San Francisco, CA USA; 7 Department of Medical Microbiology and Immunology, University of Alberta, Edmonton, Alberta Canada; 8 Department of Pathology, Dana Farber Cancer Institute and Harvard Medical School, Boston, MA USA; 9 European Research Institute for the Biology of Ageing, University Medical Center Groningen, Groningen, The Netherlands; 10 Center for Advanced Biotechnology and Medicine and Department of Chemistry and Chemical Biology, Rutgers University, Piscataway, NJ USA; 11 Charles River Laboratories, Chesterford Research Park, Saffron Walden, UK; 12 Proteros Biostructures GmbH, Martinsried, Planegg, Germany; 13 Whitehead Institute for Biomedical Research, Cambridge, MA USA; 14 Department of Structural Biology, Stanford University School of Medicine, Stanford, CA USA; 15 Department of Chemical and Systems Biology, Stanford University School of Medicine, Stanford, CA USA; 16 Stanford Cancer Institute, Stanford University School of Medicine, Stanford, CA USA; 17 Structural Biology of Selfish RNA, Department of Protein Evolution, Max Planck Institute for Biology, Tübingen, Germany; 18 Johns Hopkins University School of Medicine, Baltimore, MD USA; 19 Department of Biology, University of Texas at Arlington, Arlington, TX USA; 20 Physiology, Biophysics & Systems Biology, Weill Cornell Medicine, Weill Cornell Medical College, New York, NY USA; 21 Department of Pathology, Massachusetts General Hospital and Harvard Medical School, Boston, MA USA

**Keywords:** X-ray crystallography, Transposition, Cryoelectron microscopy

## Abstract

The LINE-1 (L1) retrotransposon is an ancient genetic parasite that has written around one-third of the human genome through a ‘copy and paste’ mechanism catalysed by its multifunctional enzyme, open reading frame 2 protein (ORF2p)^1^. ORF2p reverse transcriptase (RT) and endonuclease activities have been implicated in the pathophysiology of cancer^2,3^, autoimmunity^4,5^ and ageing^6,7^, making ORF2p a potential therapeutic target. However, a lack of structural and mechanistic knowledge has hampered efforts to rationally exploit it. We report structures of the human ORF2p ‘core’ (residues 238–1061, including the RT domain) by X-ray crystallography and cryo-electron microscopy in several conformational states. Our analyses identified two previously undescribed folded domains, extensive contacts to RNA templates and associated adaptations that contribute to unique aspects of the L1 replication cycle. Computed integrative structural models of full-length ORF2p show a dynamic closed-ring conformation that appears to open during retrotransposition. We characterize ORF2p RT inhibition and reveal its underlying structural basis. Imaging and biochemistry show that non-canonical cytosolic ORF2p RT activity can produce RNA:DNA hybrids, activating innate immune signalling through cGAS/STING and resulting in interferon production^6–8^. In contrast to retroviral RTs, L1 RT is efficiently primed by short RNAs and hairpins, which probably explains cytosolic priming. Other biochemical activities including processivity, DNA-directed polymerization, non-templated base addition and template switching together allow us to propose a revised L1 insertion model. Finally, our evolutionary analysis demonstrates structural conservation between ORF2p and other RNA- and DNA-dependent polymerases. We therefore provide key mechanistic insights into L1 polymerization and insertion, shed light on the evolutionary history of L1 and enable rational drug development targeting L1.

## Abstract (web summary)

X-ray crystallography, cryo-electron microscopy, structural modelling, biochemistry, cell biology, and evolutionary analysis enable characterization of ORF2p, the reverse transcriptase of the ancient ‘parasitic’ LINE-1 retrotransposon that has written around one-third of the human genome.

## Main

**Fig. 1:**
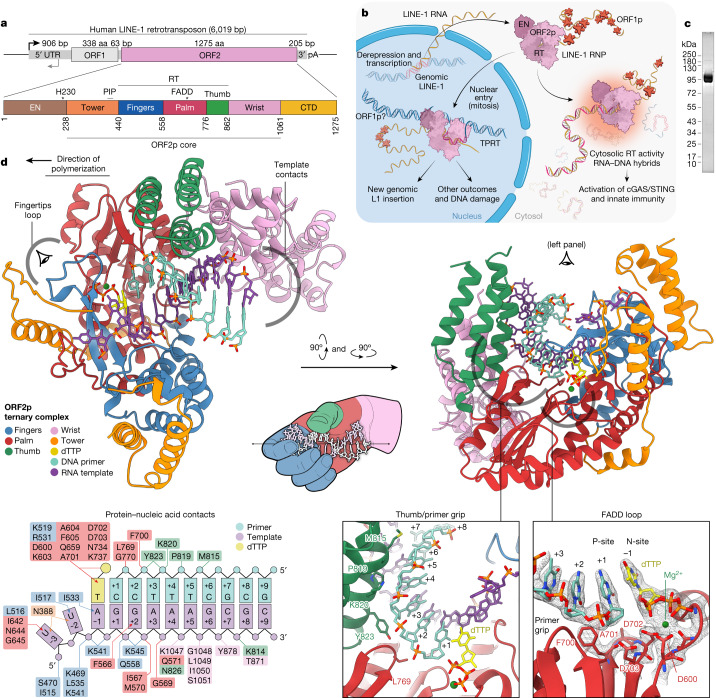
Pathogenic replication cycle of L1 and the 2.1 Å resolution crystal structure of human ORF2p core in a ternary complex. **a**, The 6 kb human L1 element contains an internal 5′ untranslated region (UTR) promoter, two proteins ORF1p and ORF2p in a bicistronic arrangement separated by 63 nt and a short 3′ UTR. **b**, Replication cycle of L1, a streamlined self-copying DNA parasite. Derepression of genomic L1s results in Pol II transcription and export of the L1 RNA, which is translated to form an RNP complex containing one copy ORF2p, a multifunctional enzyme, and many copies of ORF1p, a homotrimeric chaperone involved in nuclear entry that can form phase-separated granules. Canonically, in the nucleus, ORF2p integrates a new copy of the L1 RNA into the genome in a mechanism termed TPRT, in which cleavage by the L1 EN liberates a genomic DNA (gDNA) 3′-OH used to prime reverse transcription of the L1 RNA, followed by insertion by poorly understood mechanisms (‘Discussion’, Fig. 6). Non-canonical outcomes contribute to pathology: failed insertions and aberrant EN activity result in DNA damage and translocations, and aberrant cytosolic RT activity generates inflammatory RNA:DNA hybrids. Host proteins (not shown) are associated at every step and may repress L1 or function as essential cofactors. **c**, Sodium dodecyl sulfate polyacrylamide gel electrophoresis analysis of pure, monodisperse 97 kDa ORF2p core after size exclusion chromatography. **d**, Two new domains (tower and wrist) and three canonical RT subdomains (fingers, palm, thumb) coordinate with a hybrid duplex RNA template (purple) and DNA primer (cyan) and incoming dTTP nucleotide (yellow) for ORF2p core RT activity in the 2.1 Å resolution crystal structure in a ‘right-hand’ RT fold that is uniquely adapted. All five ORF2p core domains contact the template or primer, and numerous residues contact the incoming base; protein contacts are summarized in the inset schematic.

Recent primate transposon evolution is dominated by RNA ‘copy and paste’ retrotransposons that insert RNA intermediates into the genome by encoded reverse transcriptase (RT) activity^9^. These retrotransposons are divided into two classes: (1) endogenous retroviruses (ERVs), flanked by long terminal repeats (LTRs); and (2) the non-LTR retrotransposon long interspersed element-1 (LINE-1, L1)^1^. ERVs are no longer thought to be active in humans^1^. By contrast, each person inherits about 100 polymorphic and fixed potentially active L1s, a small subset of the approximately half a million inactive L1 copies and fragments^1^. LINEs have been coevolving with their hosts for 1–2 billion years, since the emergence of eukaryotes. Human L1 encodes two proteins, ORF1p^10^ and ORF2p, the latter having endonuclease (EN) and RT activities^11–13^, along with three other domains with unknown functions (Fig. 1a,b). ORF2p cotranslationally binds its encoding L1 RNA, a property termed ‘*cis* preference’^14–17^, forming a ribonucleoprotein (RNP) complex with many copies of ORF1 and host proteins^10,15,17–19^ (Fig. 1b). New insertions begin with the target primed reverse transcription (TPRT) priming mechanism: an EN nick on the ‘bottom’ DNA strand liberates a DNA 3′-OH used to prime RT and generate an RNA:DNA hybrid intermediate^20–23^. The details of TPRT in L1, second strand synthesis and how the resulting intermediates are resolved remain unclear, although it is known that a subsequent staggered break in the second ‘top’ DNA strand^24^ results in a characteristic target site duplication of typically less than 20 base pairs (bp) flanking L1-mediated insertions^24,25^. Despite its *cis* preference, ORF2p also binds and inserts other RNAs, including messenger RNA sequences and short interspersed element RNAs such as *Alu*.

Derepressed L1 elements can contribute to the pathology of cancer, ageing, neurodegeneration and inflammation (mechanisms posited in Fig. 1b). Consistent with this, RT inhibitors have shown promising results in model systems^6–8,26,27^ and in clinical studies of colorectal cancer^28^ and Aicardi–Goutières syndrome, a rare Mendelian interferonopathy characterized by accumulation of L1 intermediates^4,27,29^. However, our knowledge of the mechanistic details of both L1 insertion and how L1 contributes to pathophysiology is limited. The best characterized L1 relatives are insect R2 LINE elements^21^ and bacterial group II mobile introns^30,31^, which lack the amino-terminal apurinic/apyrimidinic EN (APE)-like EN of ORF2p^12,13^ and diverged from the human lineage around 700 million and 4 billion years ago, respectively. Both recognize and mobilize unique DNA and RNA sequences, limiting comparison with L1.

To address knowledge gaps in L1 biology and facilitate the potential for drug discovery, we have established systems to purify both full-length ORF2p and a minimal ‘core’, characterized ORF2p RT activity, and determined its structure using various modalities. Our investigation revealed (1) efficient RT priming by short RNAs and hairpins; (2) direct cytosolic synthesis of RNA:DNA hybrids that activate cGAS-STING, resulting in interferon production; (3) a series of conformational adaptations in the ‘right-handed’ fingers, palm and thumb RT fold that are likely to modulate biochemical activities required for the replication cycle of L1; (4) the presence of two previously undescribed domains in the RT core, which we name ‘tower’ and ‘wrist’; and (5) concerted dynamics of the N-terminal EN and carboxy-terminal domain (CTD). Informed by this structure, we elucidate the evolutionary relationships between conserved structural features in ORF2p. Our results shed light on previously enigmatic steps in the L1 replication cycle, its roles in pathophysiology and potential routes to therapeutics.

## Purification of highly active ORF2p RT

**Extended Data Fig. 1:**
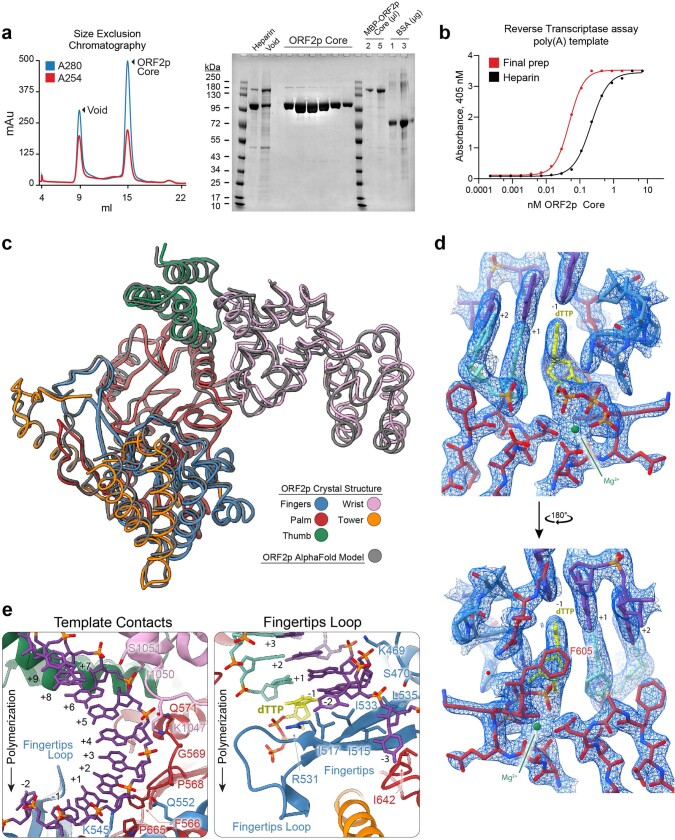
Purification and crystal structure of ORF2p core. **a**, Size exclusion chromatography (SEC) of recombinant ORF2p core (left, Superdex 200 increase 10/300 GL column, Cytiva) shows a homogenous and Gaussian peak corresponding to the expected retention time of a ~ 100 kDa monomer. SDS-PAGE analysis of peak fractions (right) shows the ORF2p core peak is >99% pure with contaminants and uncleaved MBP-ORF2p core removed in the void volume; a trace amount of uncleaved MBP-ORF2p remains in the preparation. **b**, In an ELISA-based reverse transcriptase assay (Roche), ORF2p core shows increased activity after SEC relative to heparin chromatography alone against an oligo(A) template. **c**, Comparison of ORF2p core crystal structure with AlphaFold model used for molecular replacement shows remarkable similarity, with a final root-mean-square deviation (RMSD) of 0.946 Å from the search model. ORF2p core comprises 46 secondary structural elements divided between 10 beta strands and 36 helices and is resolved from residues 251–1061 with gaps from 304–388, 799–803, 851–871, 905–912, and 923–927. **d**, 2Fo-Fc electron density map of the ORF2p core crystal with built model at a threshold of 2σ shows clear side chain density for important residues near the active site. The highlighted “gatekeeper” residue F605 sterically selects against ribonucleotides by clashing with the 2’-OH^61^, providing a rationale for ORF2p’s low RNA synthesis activity. **e**, Detailed view of key contacts between the primer and template and residues of the fingers (K541, K545, Q552), palm (F566, I567, P568, G569, M570, Q571, G660, P665) and wrist (Y878, K1047,G1048, I1050, S1051).

Previous efforts to measure ORF2p enzymatic activity have been limited by an inability to purify more than trace amounts of ORF2p RT, with limited characterization of impure enzyme indicating that ORF2p may be able to perform DNA synthesis using RNA or DNA templates^20,32,33^. Here, we optimized purification of the ORF2p core (residues 238–1061) to yield milligram quantities of more than 99% pure enzyme (Fig. 1c) that was monomeric (Extended Data Fig. 1a) and highly active against oligo(A) templates (Extended Data Fig. 1b), enabling structural and kinetic analyses, as well as single-base-resolution assays with various substrates and inhibitors.

## A 2.1 Å crystal structure of the ORF2p core

**Fig. 2:**
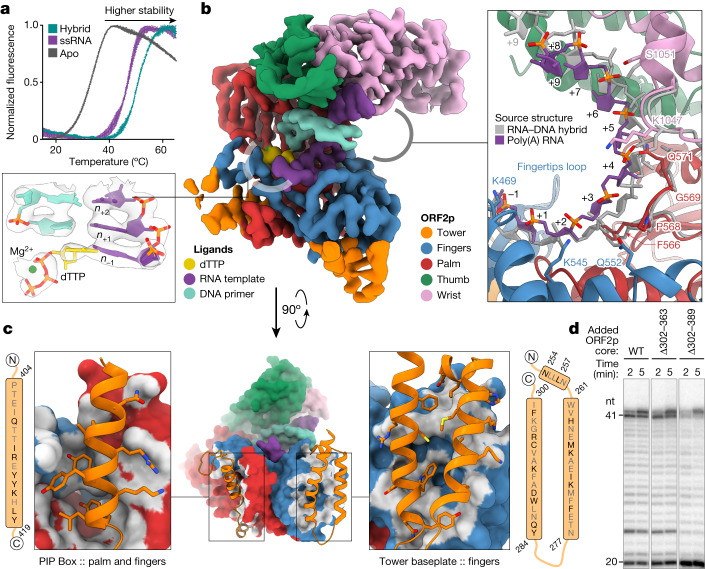
Cryo-EM structures of ORF2p core in *apo*, ssRNA and RNA:DNA hybrid-bound states. **a**, ORF2p is unstable in the absence of nucleic acids (*T*_m_ = 34.1 °C ± 0.35) but is significantly stabilized by the binding of ssRNA (*T*_m_ = 47.5 °C ± 0.32) and RNA:DNA heteroduplex (*T*_m_ = 50.2 °C ± 0.1) as determined by differential scanning fluorimetry. **b**, Density map of the 3.3 Å cryo-EM reconstruction of the ORF2p core in ternary complex with RNA template–DNA primer heteroduplex and dTTP, coloured by proximity to modelled domains with fit atomic model (inset left), which shows clear density for primer, template and dTTP base for addition. Deviation of RNA template (inset right) in the ssRNA cryo-EM structure (purple) from the heteroduplex (grey, backbone RMSD of 3.76 Å). **c**, Structural schematic of the contacts between the PIP box (inset left) and baseplate (inset right) subdomains of the ORF2p tower with the canonical RT subdomains of palm and fingers. **d**, Denaturing gel RT assay with ORF2p core (wild type; WT) or tower deletions (∆302–363, ∆302–389) shows similar RT activity with and without the tower and tower lock. Data are representative of three (**a**) and two (**d**) independent experiments.

To characterize domains of ORF2p of previously unknown function, understand how these domains interact during priming and reverse transcription, and elucidate the structural basis of differential RT inhibition as a basis for rational drug design, we solved the crystal structure of ORF2p core in an active configuration, using an AlphaFold model for molecular replacement (Extended Data Table 1 and Extended Data Fig. 1c). The structure represents a ternary complex with an incoming deoxythymidine triphosphate (dTTP) nucleotide and a template–primer heteroduplex containing a three-nucleotide (nt) 5′ overhang in the RNA template and 3′ dideoxy-terminated DNA primer. The complex crystallized in space group C2, with one monomer in the asymmetric unit. The structure (Fig. 1d) reveals the fingers, palm and thumb of a characteristic right-hand RT fold but also shows key differences compared with other RTs. Two folded domains which we name ‘wrist’ (863–1061) and ‘tower’ (240–440, Figs. 1d and 2, described below) are absent from other known structures of RT enzymes from viruses or mobile elements. All five domains make extensive contact with the bound nucleic acid (Supplementary Methods, Fig. 1d inset diagram and Extended Data Fig. 1e).

## Five ORF2p core domains all bind nucleic acid

As in other RTs, the fingers, palm and thumb domains form a groove that cradles the RNA template–DNA primer heteroduplex. Nucleotide positions in the template and primer are numbered *n*_−3_ to *n*_+10_ relative to 5′, and *n*_−1_ is the templating ribonucleoside and incoming deoxyribonucleoside triphosphate (dNTP) (Fig. 1d, insets, and Extended Data Fig. 1e). We identify template contacts in both new domains: the tower contacts the 5′ RNA template at the *n*_−3_ base, and the wrist makes multiple contacts with the downstream region of the template (3′ end). The overall configuration of the active site and resultant catalytic mechanism are highly conserved throughout RTs and related polymerases^30,34^: in a region of the palm termed the N-site, the incoming dNTP base pairs with the *n*_−1_ base on the template and is poised for covalent linkage to the 3′ hydroxyl of the primer *n*_+1_ deoxyribose ring. The catalytic triad of aspartic acids (D600, D702, D703) resides at the active site and coordinates a Mg^2+^ ion and the dNTP; D702 and D703 form the base of the FADD loop (Fig. 1d, inset). The gatekeeping residue F605 has an aromatic side chain that selects against ribonucleotides with a 2′ hydroxyl, which probably explains the inability of ORF2p to function as an RNA-dependent RNA polymerase (RdRp); Extended Data Figs. 1d and 4c and Supplementary Fig. 3c). The 5′ upstream RNA template enters ORF2p above the fingertips, with eight residues contacting *n*_−3_, including hydrogen bonding between the base and an extended palm loop and the tower. The template next interacts with the R0 loop, which forms a ‘lid’ over the template RNA. This loop is a portion of the R0 region, also called the N-terminal extension (NTE)-0, which is found in non-LTR retrotransposons, the group IIC intron and HCV RdRp, but not in viral RTs^30^, and has been demonstrated to be important for template jumping and/or switching activity^35,36^ (‘Domain comparison of ORF2p and other RTs’). The downstream template makes extensive interactions continuing until the *n*_+8_ position with fingers, palm, wrist and thumb (Fig. 1, diagram). The DNA primer is contacted through the *n*_+5_ position, held upstream by the primer grip and downstream by the thumb with the helix clamp at its base.

## Structure of the L1 wrist domain

The wrist domain (863–1061) has not been previously recognized, although experiments deleting large portions of the wrist and the subsequent CTD have shown that both domains are required for efficient retrotransposition^37^. Scanning mutagenesis also has shown numerous wrist regions required for retrotransposition^38^. The fold consists of 12 helices anchored to the RT through interactions with the thumb helices and palm through a helix at residues 573–581 and a short β turn at residues 688–695. Searches on similarity servers Dali and Foldseek show weak similarity to a sterile alpha motif-like domain, indicating possible roles in nucleic acid binding or protein–protein interactions. In the structure, the wrist makes numerous backbone contacts with the RNA template through *n*_+4_ to *n*_+7_, and trialanine mutants spanning these residues have resulted in reduced or no retrotransposition activity^38^.

## ORF2p cryo-electron microscopy structures in three states

**Extended Data Fig. 2:**
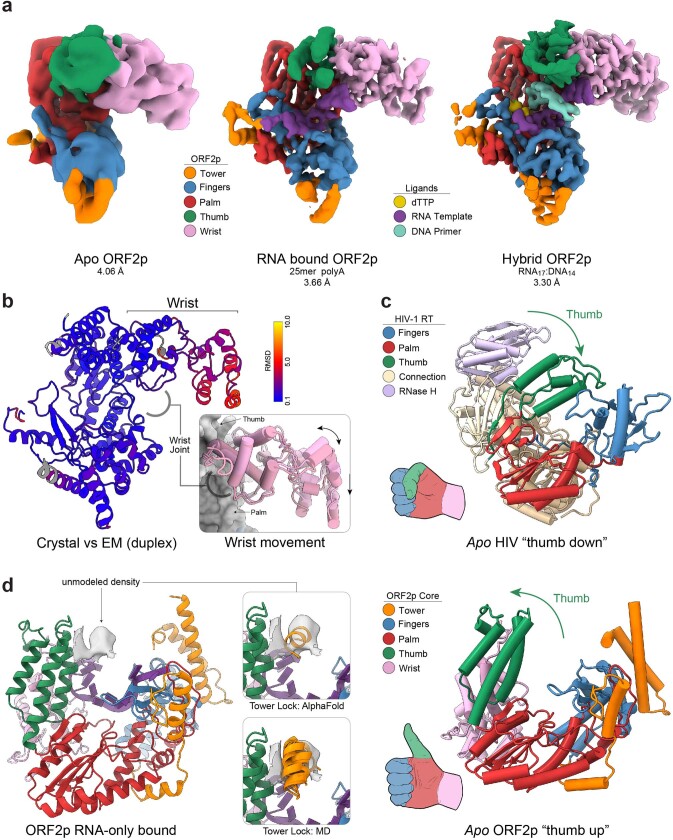
Comparison of cryo-EM maps and models. **a**, Final cryo-EM maps of ORF2p *apo* (left), bound to ssRNA (middle) or template: primer hybrid (right) colored by corresponding ORF2p region. There is an expected clear lack of density in the active site for the *apo* ORF2p map and in the primer-binding region for the ssRNA map. Consistent with *apo* ORF2p being unstable in vitro, it represents the lowest resolution reconstruction, and no corresponding atomic model was built, but rigid body fitting of the hybrid-bound atomic model fills the density. **b**, Coloring of the refined ORF2p structural model by RMSD from the ORF2p crystal structure reveals little difference in the thumb-fingers-palm-thumb subdomains (RMSD = 1.01 Å) but significant deviation of the wrist (RMSD = 4.01 Å). Superposition of the crystal and cryo-EM derived structures (inset) shows a rotational motion of 4 Å and an upwards translation of 7.5 Å occurs in the distal wrist; the palm-adjacent wrist helices are completely superposed, and both structures maintain the same template contacts. **c**, Comparison of the structure of *apo* HIV-1 RT (PDB: 1dlo^62^) and rigid body fit *apo* ORF2p core. In *apo* HIV-1 RT, the enzyme is in an inactive conformation with the thumb occupying the active site or” thumb down”; alternatively, *apo* ORF2p closely resembles the active form of the enzyme with the “thumb up”. This “thumb up” form would not require a conformational change for accepting incoming template, like in HIV-1 RT. **d**, Orphan density from the ORF2p core-ssRNA map low-pass filtered to 4.5 Å. This density is consistent with the predicted location of the tower lock from AlphaFold (inset, top) and molecular dynamics simulations of the full tower domain cluster the tower lock near this density (bottom). This location is also consistent with the position of the R2Bm tower lock portion of the tower-like domain, which binds to the 3’UTR RNA (PDB 8gh6)^22^.

We next measured the thermal stability of ORF2p in differential scanning fluorometry assays, in which heat-induced denaturation results in increasing exposure of the hydrophobic core of the protein and resultant binding and fluorescence of the SYPRO Orange dye. *Apo* ORF2p, lacking bound nucleic acid, was unstable, with a melting temperature (*T*_m_) of 34.1 ± 0.4 °C. ORF2p was markedly stabilized by binding single-stranded RNA (ssRNA) (Δ*T*_m_ = 14.4 ± 0.6 °C) and further stabilized by binding an RNA:DNA hybrid (Fig. 2a; Δ*T*_m_ from ssRNA-bound = 2.7 ± 0.4 °C, Δ*T*_m_ from *apo* = 16.1 ± 0.4 °C). To understand the structural changes resulting from binding of the primer and template, we used single-particle cryo-electron microscopy (cryo-EM; Extended Data Table 2 and Supplementary Figs. 1 and 2) to obtain reconstructions of ORF2p in three distinct states: in an active ternary complex with incoming dTTP and template–primer; bound to oligo-25(A) ssRNA; and in *apo* form (to 3.30, 3.66 and 4.06 Å resolution, respectively; Extended Data Fig. 2a). This is the first reported structure of an RT bound with ssRNA in the active site.

The density for the active ternary complex was complete and facilitated building of a structural model with clear density for the incoming dNTP, Mg^2+^ and template–primer (Fig. 2b, inset left). The cryo-EM-derived atomic model was predominantly indistinguishable from the crystal structure, with an overall root mean square deviation (RMSD) of 1.01 Å in tower–fingers–palm–thumb. There was apparent flexibility between the wrist and the rest of ORF2p, but the wrist fold itself was predominantly unchanged between the two structures (wrist backbone RMSD of 4.04 Å, aligned wrist RMSD = 1.01 Å, overall RMSD including wrist 3.68 Å; Extended Data Fig. 2b). Comparison of heteroduplex and ssRNA-bound states revealed distinct template paths (template RMSD of 3.76 Å; Fig. 2b, inset right) but overall maintenance of similar contacts through movement of flexible loops, notably in the palm and wrist domains. Intriguingly, although the structure was not as high resolution, the *apo* ORF2p was found in a ‘thumb up’ conformation, in which the template binding and active sites were accessible; by contrast, *apo* viral RTs assumed an inactive ‘thumb down’ conformation, in which the thumb occupied the nucleic-acid-binding site (Extended Data Fig. 2c,d). This ‘thumb up’ conformation, the instability of the *apo* protein and tight RNA binding are likely to contribute to the *cis* preference of L1.

## Structure of the L1 tower domain

**Extended Data Fig. 3:**
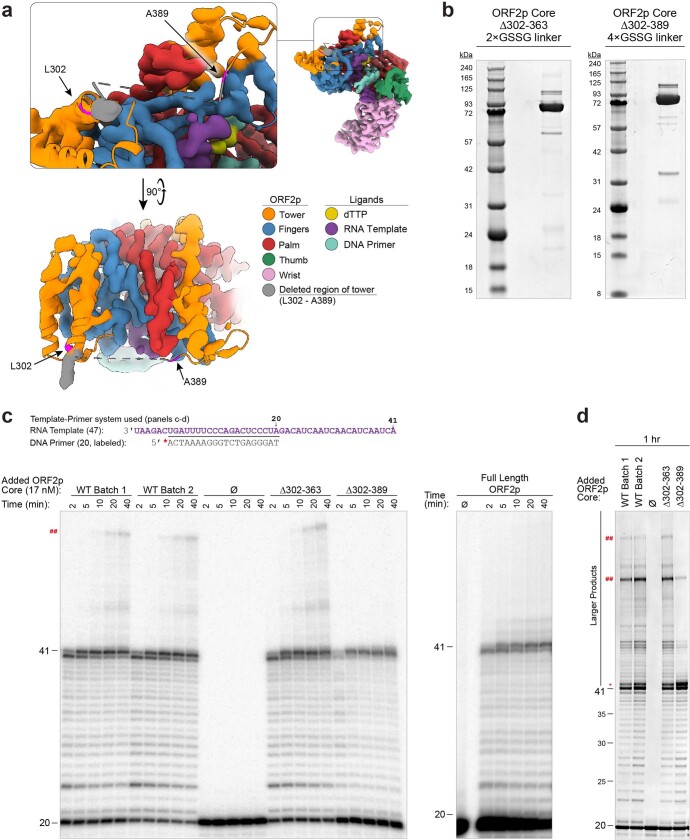
Design and characterization of the ORF2p tower domain deletions reveal it is not required for RT. To test the role of the tower and tower lock in reverse transcription, ORF2p core constructs where the tower was deleted at two different points were designed. **a**, The maximal tower deletion construct (∆302–389) represents removal of the unresolved tower and the tower lock residues from both the EM and crystal structures as evidenced by mapping the deletion back onto the EM structure of ORF2. The shorter tower deletion ∆302–363 deletes the tower but preserves the lock. **b**, SDS-PAGE analysis of monodisperse ORF2p core tower deletion constructs show relatively pure enzyme ( > 90%). **c**, Comparison of wildtype ORF2p core and full length versus ∆302–363 and ∆302–389 (cropped in Fig. 2d) shows similar RT activity between all constructs with little difference in efficiency of formation of 41 nt full length products over time; full length and ∆302–389 are slightly less specifically active, which may be due to batch effects, contaminants, or concentration estimation errors; 17 nM of purified ORF2p core constructs was reacted with 0.1 μM dNTP mixture and samples taken over time. Asterisk (*) ^32^P-labeled 5’-end of the primer. **d**, Comparison of wildtype and tower deletion ORF2p core constructs under longer reaction conditions with higher concentrations of enzyme and nucleotide shows all constructs form full length, NTA ( + and above), and template jumping/switching products (##), although the yield of these larger products correlates with specific activity; it appears that here and in panel (c), deletion of both the tower and lock (∆302–389) may selectively negatively impact template jumping/switching activity, although this may be attributable to either lock or the way in which it was deleted, and further investigation is warranted. These reactions are 1 h with 3-fold more enzyme (50 nM) and 10-fold more dNTPs (1 μM). Scanned gel images are cropped and corrected for distortion artifacts with contrast uniformly increased to facilitate the visualization of minor products.

ORF2p contains an N-terminal APE-like EN^13^ and is the first such retrotransposon to be structurally characterized; other classes of non-LTR retrotransposons have C-terminal restriction-like ENs (RLE)^22–24^. The tower domain (239–440) corresponds to the region between the EN and RT domains and consists of four key components, (1) a baseplate (residues 254–300), (2) the protruding tower helices (residues 301–370), (3) the subsequent tower lock (residues 374–382) and (4) a PIP box helix (PCNA-interacting protein, residues 404–419), and encompasses regions previously termed ‘cryptic’ or ‘desert’^38,39^. Structure similarity searches did not show significant similarities to other proteins. The tower baseplate (Fig. 2c) was resolved to residue 304 in the crystal and 310 in our EM model. The tower and lock were anchored to RT at two points: (1) by the baseplate to fingers through mostly hydrophobic contacts, and (2) by PIP to the palm and fingers by a mix of hydrophobic and polar interactions. Mutation of key residues in the baseplate reduce retrotransposition^39^, and PIP orchestrates an ORF2p–PCNA interaction that depends on EN and RT activities and is required for retrotransposition^17,18,39^. AlphaFold2 modelling indicates that the intervening helices form an elongated hairpin-like tower, which seems to be flexible. Modelling using molecular dynamics simulations and AlphaFold indicated that the tower lock is consistent with orphan density above the *n*_+4_ base in low-pass filtered cryo-EM maps of ssRNA-bound ORF2p and may therefore fold down and ‘cap’ the RNA template (Extended Data Fig. 2d). A functionally similar tower lock was present in the smaller tower-like domain in R2, despite sequence divergence (see domain comparison below)^22,23^. To test the importance of the unresolved tower and tower lock on RT activity, we purified ORF2p mutants that truncated the tower (Δ302–363) or tower and tower lock (Δ302–389), replacing them with short flexible linkers (Extended Data Fig. 3a,b). Both constructs were active similarly to the wild type in RT assays (Fig. 2d and Extended Data Fig. 3c,d), but trialanine mutagenesis has shown no retrotransposition with mutants in various regions of the tower and in the lock^38^. Together, these data demonstrate that the ORF2p tower is important for L1 retrotransposition but not RT activity. They also indicate that ORF2p fragments consisting of portions of the tower base may be able to bind to the rest of ORF2p in *trans*, enabling ‘bipartile’ *Alu* retrotransposition^39^.

## ORF2p RT and polymerase activities

**Fig. 3:**
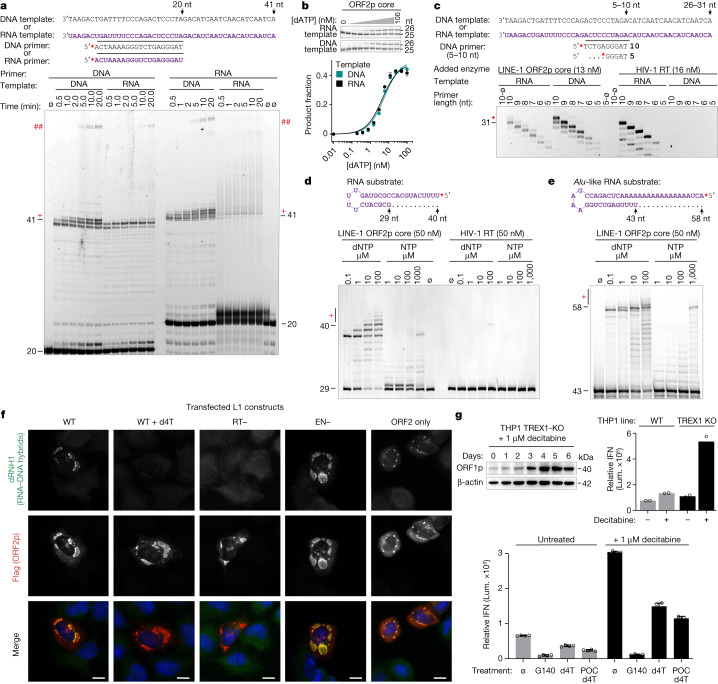
L1 biochemical activities, priming and cytoplasmic reverse transcription of L1. **a**, Denaturing gel ORF2p RT assay. ORF2p core was an efficient DNA polymerase on all template–primer combinations; RNA priming on an RNA template was reduced but remained significant, with time-dependent full template-length (FTL) reaction products. NTA (+) and template jumping/switching (##) larger products were clearer on longer exposure (Extended Data Figs. 3–5 and Supplementary Figs. 3 and 4). **b**, ORF2p core (33 nM) single dATP incorporation kinetics with RNA or DNA template and 20 nt DNA primer. **c**, Extension of very short (5–10 nt) primers, pre-annealed to DNA or RNA templates, by ORF2p and HIV-1 RT; *n* = 4 (DNA), *n* = 3 (RNA) independent samples over two experiments. **d**, ORF2p RT assay showing efficient elongation of an RNA hairpin to FTL; HIV-1 RT showed minimal elongation. **e**, ORF2p efficiently extended a uridylated *Alu*-derived RNA hairpin. Ribonucleoside triphosphate incorporation was strongly selected against. **f**, Immunofluorescence of HeLa cells transfected for 24 h with WT or mutant L1 constructs (*ORFeus*-Hs) stained for RNA:DNA hybrids with catalytically inactive RNase H1 (dRNH1) and ORF2p (Flag). Cytosolic RNA:DNA hybrids colocalized with ORF2p, depended on RT activity, were ablated by 50 µM d4T and did not depend on EN activity, ruling out a nuclear origin. Hybrids were most prominent in L1 granules but were still present when ORF1p was removed (ORF2 only, monocistronic). **g**, Top left, ORF1p induction by 1 µM decitabine in THP1 monocytes. Concomitantly, interferon (IFN) production increased (secreted luciferase reporter, top right; lum., luminescence), further augmented by knockout of TREX1, a nuclease that degrades L1 cDNA. Bottom: treatment of these cells with 10 µM cGAS inhibitor G140 or 50 µM d4T RTI reduced baseline and decitabine-induced IFN production; 10 µM POC d4T, a more efficiently triphosphorylated d4T prodrug, reduced IFN further. For IFN, *n* = 4 biologically independent samples over two experiments. Scale bars, 10 μm. All error bars indicate s.d.

**Extended Data Fig. 4:**
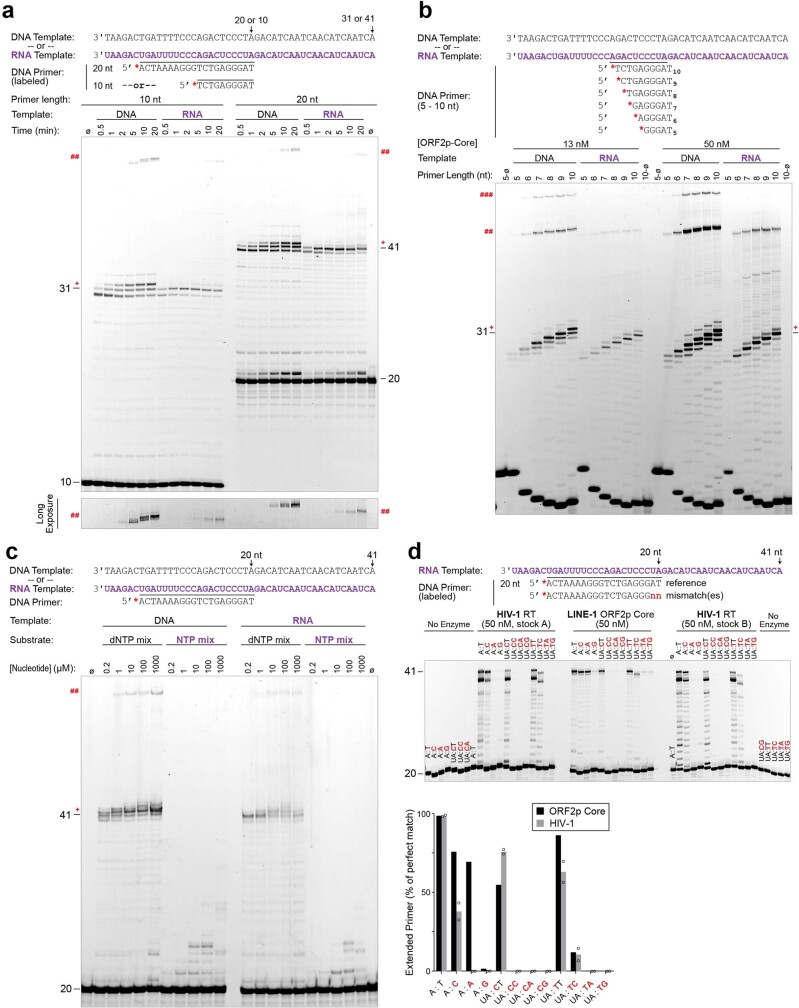
Priming requirements and mismatch tolerance of ORF2p core. **a**, Comparison of 10 nt vs 20 nt DNA primers reveals little difference in efficiency of formation of products, including larger template jumping/switching products (##). **b**, ORF2p performs DNA synthesis with 5–10 nt DNA primers, although 5 nt and, to a lesser extent 6 nt, are slightly less efficiently used. As seen consistently above, RNA templates are slightly less efficient than DNA. Higher concentrations of ORF2p core result in higher activity in all conditions and more template jumping/switching products. Scanned gel images are cropped and corrected for distortion artifacts with contrast uniformly increased to facilitate the visualization of minor products. (* indicates Cy5 label, all panels). **c**, RNA synthesis is strongly selected against, as indicated by nucleotide (dNTP or NTP) incorporation activity of LINE-1 RT on DNA or RNA using a DNA primer. Denaturing PAGE migration pattern of the reaction products generated after 5 min of dNTP or NTP incorporation along DNA and RNA templates using 20-nt primers. **d**, Priming activity of ORF2p and HIV-1 with one or two terminal mismatches; two enzyme preps of HIV-1 RT are compared to ORF2p, and additional unextended substrates are shown. L1 tolerates all terminal mismatches against an A template to some extent, as well as some penultimate mismatches; A:G is inefficient. In contrast, HIV-1 is less tolerant of A:A and A:G terminal and U:A and U:G penultimate mismatches; n = 1 (LINE-1) and n = 2 (HIV-1) points quantified from 2 independent experiments.

**Extended Data Fig. 5:**
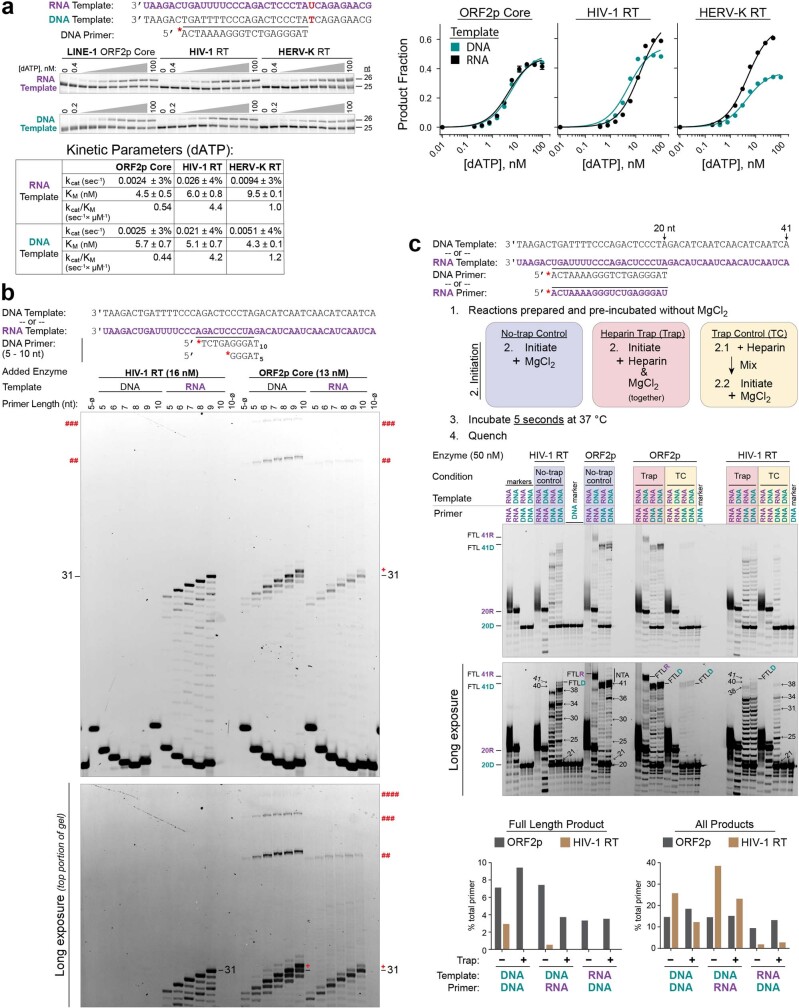
Comparative enzymology of ORF2p RT with HIV-1 and HERV-K. **a**, Single nucleotide incorporation kinetic curves and parameters of dATP with 36-nt RNA or DNA template and 20-nt DNA primer with ORF2p core (33 nM), HIV-1 RT (4 nM) and HERV-K RT (12 nM). For each enzyme, Michaelis-Menten parameter k_cat_/*K*_*M*_ is nearly identical on both templates (n = 3 (DNA template) and n = 4 (RNA template) independent samples over 2 independent experiments; data represented as mean ± SD). **b**, Comparison of HIV-1 RT and ORF2p in extension of very short (5–10 nt) primers, pre-annealed to DNA and RNA templates. ORF2p extends all DNA and RNA primer lengths, with somewhat reduced efficiency at 5 nt and, to a lesser extent, 6 nt. In contrast, HIV-1 does not extend the same DNA:DNA template:primer mixes of these lengths and does not extend 5 nt and has reduced activity with 6 nt DNA primers on RNA templates. ORF2p also makes NTA (+) and template jumping/switching (##) larger products; more visible on longer exposure. Notably, neither of these larger products are detectable with HIV-1 RT; quantification represents n = 3 (DNA) and n = 4 (RNA) samples from two independent experiments. **c**, Heparin trap processivity assay for ORF2p vs HIV-1 RT; heparin sulfate is a negatively charged sugar polymer that competes for nucleic acid binding sites. The indicated RNA or DNA primers and templates were pre-annealed and reactions were prepared and preincubated as indicated, then initiated with Mg^2+^ as a control, with heparin and Mg^2+^ together, or with a two-step “Trap control” procedure in which heparin and Mg^2+^ are added sequentially. Reactions are quenched after 5 seconds. At this very short time point, ORF2p produces full template length product (FTL, 3–9% of total signal in the lane in all conditions) and is unaffected by the heparin trap; in contrast, HIV-1 RT produces 0–3% FTL product without trap and no detectable FTL product with trap. When all products are quantified, HIV-1 extends 21–37% of primers, and this is roughly halved by the heparin trap; ORF2p extends ~10–18% of primers and is unaffected by the trap. In the trap control (TC) RNA template:DNA primer lanes, HIV- 1 performs a small amount of residual RT, consistent with a distributive pattern of synthesis, whereas ORF2p is inhibited, bound to the heparin trap. These are all consistent with high processivity for ORF2p and low- processivity distributive pattern synthesis for HIV-1 RT. Asterisk (*) Cy5-5’-label on primer. n = 1 quantified samples shown representative of two independent experiments.

ORF2p can polymerize DNA on RNA or DNA templates (RT or pol activities) with approximately equal efficiency using either DNA or RNA primers. RNA priming of cDNA synthesis on an RNA template is less efficient but still occurs at a significant rate (Fig. 3a and Supplementary Fig. 3a,b). This reduced but significant level of L1 ORF2p RNA priming on RNA templates is in stark contrast with HIV-1 RT, for which only specialized RNA primers are used in initiation, at an efficiency reduced by orders of magnitude^40^. L1 ORF2p RNA synthesis (RdRp activity) was strongly selected against, with minimal detectable activity (Extended Data Fig. 4c and Supplementary Fig. 3c). In single-nucleotide additions with long 20 nt primers, ORF2p had no apparent preference for an RNA or DNA template. HIV-1 RT and human ERV K (HERV-K) RT^34^ also accept both templates and have roughly ten-fold and two-fold higher efficiency of single nucleotide incorporations than L1 ORF2p, respectively. By contrast, whereas ORF2p efficiently extended 5 nt DNA primers on DNA or RNA templates, HIV-1 RT had markedly reduced efficiency with 6 nt primers in RT reactions, was incapable of reverse transcribing a 5 nt primer, and did not extend primers 5–10 nt long on DNA templates (Extended Data Figs. 4a,b and 5a,b). ORF2p was highly processive and unaffected by a heparin competitor, whereas HIV-1 RT was significantly less processive at baseline and did not produce full-length template with a heparin competitor in any condition (Extended Data Fig. 5c).

ORF2p also consistently produced larger products of two types, which increased with both longer reaction times and higher concentrations of reaction components: (1) non-templated addition (NTA, or 3′ tailing), in which single bases are added beyond the 5′ end of the template; and (2) template jumping or template switching products, in which polymerization of the same cDNA strand (copy of template_1_) continues on a new incoming template molecule (template_2_) that is accepted and copied, making a concatemer (copy of template_1_ + copy of template_2_ (Supplementary Fig. 4). No NTA or template jumping activities of ORF2p were detectable with HIV-1 RT (Extended Data Fig. 5b). These activities have been well characterized in other non-LTR transposons and are thought to be important for completion of an insertion (‘Discussion’) but have not previously been shown for ORF2p. NTA activity mechanistically explains previously reported ‘5′ extra nucleotides’ or ‘microhomologies’ observed in naturally occurring^25^ and engineered L1 insertions^41,42^.

ORF2p is known to tolerate some terminal mismatches in priming in crude RNP complex preparations^15,16^. In assays with an RNA template terminating in A, ORF2p showed little discrimination against terminal mismatches, with the exception of A:G, which retained some detectable activity. These results are similar to those of previous studies using RNP preparations^16^, in which the predominant template was presumed to be the poly(A) tail, and the similarity between the two results is evidence that most ORF2p in L1 RNP preparations rests on the poly(A) tail^15–17^. C:U and T:U internal mismatches at the second-to-last position are also tolerated, along with a UA:TC double mismatch, to a lesser extent. Overall, ORF2p is similarly active to HIV-1 RT but tolerates more mismatches (A:A and A:G mismatches are not tolerated by HIV-1 RT; Extended Data Fig. 4d). This reduced specificity may facilitate priming against diverse cellular sequences.

## Requirements for ORF2p priming

ORF2p efficiently extends DNA primers as short as 5 nt on RNA or DNA templates, with slightly lower efficiency at 5 and 6 nt than at 7–20 nt (Fig. 3c and Extended Data Figs. 4b and 5b). This is consistent with requirements of 4–6 bp annealing seen in RNP preparation assays, in which the predominant template is assumed to be the poly(A) tail^16^, and with the five primer bases that contact ORF2p (Fig. 1d). These priming results led us to investigate whether L1 ORF2p might directly accept and extend short RNA hairpin substrates. ORF2p efficiently extended a previously published 29 nt RNA hairpin containing a 7 nt duplex (Fig. 3d) and a similar hairpin derived from the substrates tested above (Supplementary Fig. 5), even at the lowest dNTP concentration tested (0.1 µM), which was at least ten-fold lower than the physiologic dNTP concentration^43^. This activity was barely detectable with HIV-1 RT at 100 µM, a difference in activity of at least four orders of magnitude; by contrast, the two enzyme preparations were similarly active in RT reactions (Fig. 3d and Extended Data Figs. 4d and 5b). As recent studies report cytosolic synthesis of *Alu* cDNA and indicate possible priming against the oligo(A) tail by the pol-III terminal U-tract^26^, we tested an *Alu*-derived sequence and found that this hairpin was also efficiently extended by ORF2p (Fig. 3e and Supplementary Fig. 5). In all cases, RNA synthesis was strongly selected against, although more activity was consistently seen at 1 mM NTPs; this concentration is likely to be supraphysiologic for all but ATP^43^. Together, these results demonstrate that ORF2p can synthesize cDNA primed only by short RNA sequences and hairpins at physiologic concentrations of dNTPs, providing a potential mechanistic basis for its cytosolic RT activity^6,7,26^.

## ORF2p synthesizes cDNA in the cytosol

**Extended Data Fig. 6:**
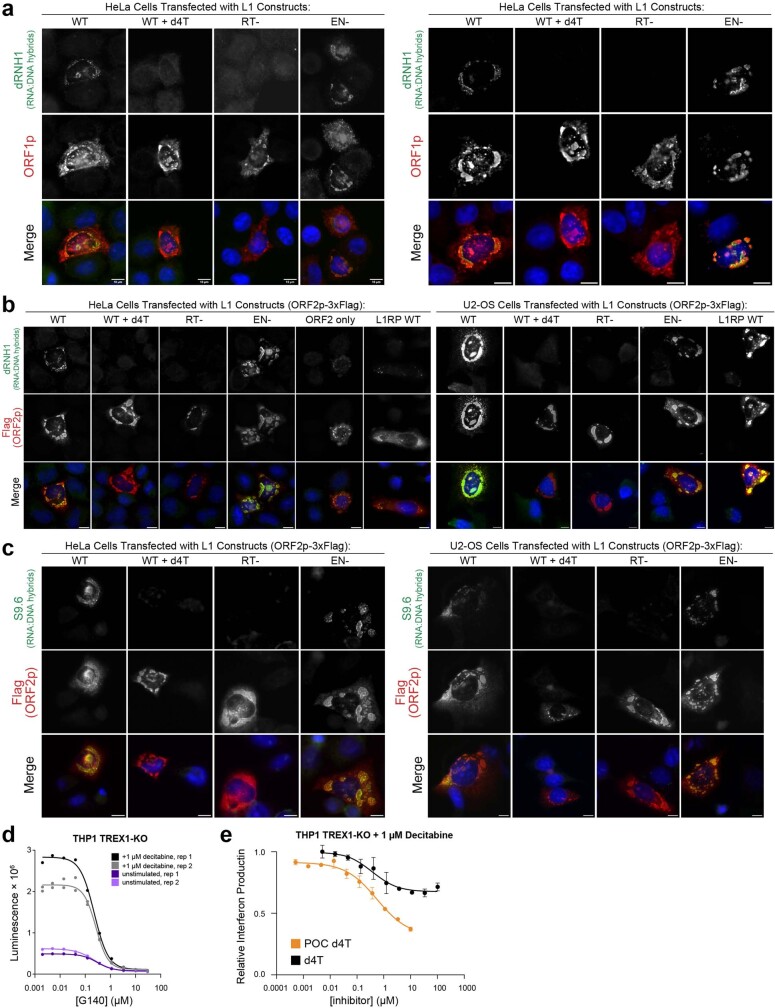
Cytoplasmic RT activity of ORF2p and activation of interferon. **a**-**c** Indirect immunofluorescence of cells transiently transfected with plasmids expressing the indicated L1 constructs and stained for RNA-DNA hybrids and ORF2p or ORF1p using two different hybrid detection reagents demonstrates cytosolic synthesis. Constructs all include C-terminal 3C-3xFlag tag on ORF2p and are synthetic *ORFeus*-Hs^63^ sequence except where L1RP is indicated (L1 retinitis pigmentosa locus, AF148856, pLD564^17^). Cells were fixed in methanol and stained 24 h post transfection with the indicated constructs. Images are representative from 4 independent experiments. D4T RTI treatment is 50 µM, added at the time of transfection. RT- is L1 with D702Y mutant ORF2, EN- is L1 with double E43S + D145N mutant ORF2. **a**, ORF1p co-stain in HeLa cells with dRNH1 (catalytically inactive human Rnase H1 fused to GFP^64^). **b**, ORF2p (Flag) with dRNH1 co-stain in HeLa and U2-OS cells. **c**, ORF2p (Flag) with S9.6 co-stain in HeLa and U2-OS cells. **d**, Inhibition of interferon signaling in THP1 cells with cGAS inhibitor G140, with and without decitabine treatment; raw luciferase data are shown, n = 4 biologically independent samples from two independent experiments; all points shown. IC50s for G140 are 0.23-0.30 µM. **e**, Relative interferon production from titrations of d4T vs POC d4T prodrug [d4T bis(isopropoxycarbonyloxymethyl)phosphate] in TREX1 knockout THP1 cells treated for 5 days with 1 µM decitabine plus the indicated concentration of drug; normalized luciferase data from n = 4 biologically independent samples representative of two independent experiments; error bars are mean ± SD.

Various cytosolic single-stranded DNAs (ssDNAs), double-stranded nucleic acids and *Alu* cDNAs have been identified in senescent cells^6,7^, retinal cells^26^ and neural progenitors^27^, along with L1 ORF1 protein. Although RT inhibitors often reduce or ablate cDNA levels, their origin has remained uncertain. We transfected HeLa and U2-OS cells with plasmids expressing L1 and found robust cytosolic RNA:DNA hybrids in transfected cells that colocalized with both L1 proteins, depended on RT activity, and were unaffected by loss of EN activity. Their formation was inhibited by 50 µM d4T treatment (Fig. 3f and Extended Data Fig. 6a–c). Hybrids were seen using synthetic *ORFeus*-Hs L1 and native L1RP sequences and with two different detection reagents: S9.6, a well-established monoclonal antibody known also to bind dsRNA under some conditions, and purified catalytically inactive human RNase H1 (dRNH1), which has recently been reported to be more specific for hybrids in imaging experiments. Hybrids were also detectable in some cells in smaller punctae when ORF2p was expressed in the absence of ORF1 (Fig. 3f and Extended Data Fig. 6a–c). As EN-independent retrotransposition occurs at levels at least 100-fold lower than wild type^44^, these results rule out a nuclear origin for these cytosolic hybrids and demonstrate that L1 can directly synthesize RNA:DNA hybrids in the cytosol.

## Synthesized cDNAs activate cGAS/STING

To investigate the consequences of cytosolic L1 RT activity, we used a secreted luciferase interferon reporter in THP1 cells, a leukaemia cell line with monocytic differentiation. Treating THP1 cells with 1 µM decitabine derepresses L1 expression by preventing DNA methylation during replication and results in interferon production^28,45,46^ (Fig. 3g). Knockout of TREX1 (three-prime repair exonuclease 1), a nuclease that is mutated in Aicardi–Goutières syndrome and systemic lupus erythematous and that has been shown to degrade cytosolic L1 DNA^4,27,29^, increased both baseline and decitabine-induced interferon levels (Fig. 3g). Both baseline and decitabine-induced interferon levels were reduced by treatment with a cGAS inhibitor (10 µM G140) or RT inhibitor (RTI; 50 µM d4T) (Fig. 3g and Extended Data Fig. 6d,e). As d4T potency was modest in this assay, we tested whether triphosphorylation of d4T was limiting inhibition by synthesizing a POC prodrug of d4T (POC d4T (d4T bis(isopropoxycarbonyloxymethyl)phosphate; Supplementary Fig. 6b)). POC d4T was approximately 30-fold more potent than d4T in suppressing interferon secretion, which provides compelling evidence that d4T triphosphate is the active form that inhibits ORF2p (Fig. 3g and Extended Data Fig. 6e). Together, these results demonstrate  that cytosolic cDNA synthesis by L1 results in interferon production through the cGAS/STING pathway.

## In vitro inhibition of ORF2p

**Fig. 4:**
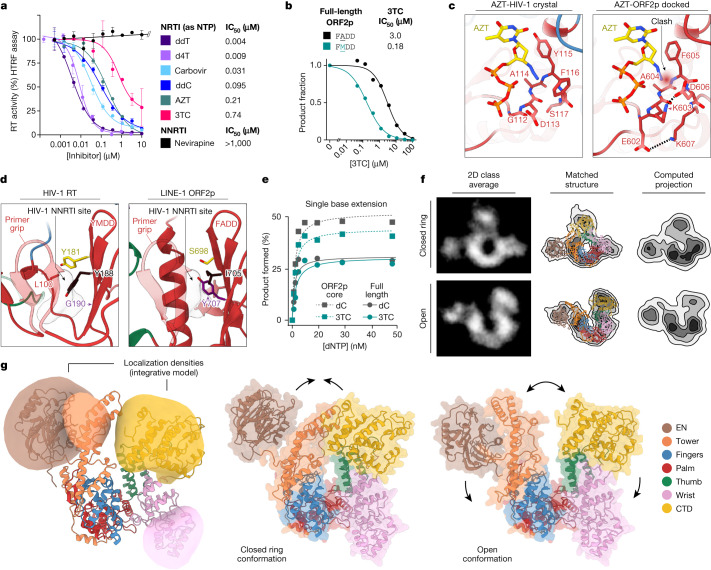
Inhibition and structure of full-length ORF2p. **a**, The ORF2p core was inhibited by NRTIs but not allosteric NNRTI HIV inhibitors in vitro according to homogeneous time-resolved fluorescence assay (*n* = 3 wells). **b**, 3TC inhibition in gel-based RT assay of full-length ORF2p WT (FADD) or HIV-like (FMDD). Although both were efficient RTs, 3TC more potently inhibited HIV-like FMDD than WT ORF2p. **c**, Structural basis for poor L1 inhibition by AZT. Crystal structure of AZT triphosphate bound to HIV-1 RT (PDB 5I42) versus model of AZT triphosphate bound to L1 ORF2p. A clash between the 3′-azido and ORF2p F605 backbone NH is highlighted. Dashed lines indicate salt bridges rigidifying the ORF2p pocket. **d**, Comparison of the HIV-1 RT NNRTI-binding region with ORF2p. Left, HIV-1 RT in the NNRTI-unbound conformation (PDB 7LRI). Residues involved in NNRTI-resistance are highlighted; space occupied by HIV-1-bound nevirapine is shadowed (PDB 4PUO). Right, equivalent region in L1 ORF2p. The long α-helix corresponds to residues 572–588 in ORF2p. Residues analogous to those in HIV-1 RT are labelled. **e**, Quantification of single-nucleotide incorporation RT assay showing that purified ORF2p core and full-length ORF2p are similarly active in incorporation of dC or 3TC nucleotides. **f**,**g**, Integrative modelling of the full-length ORF2p using Integrative Modeling Platform software, combining data from AlphaFold, molecular dynamics simulations, cryo-EM and cross-linking mass spectrometry generated an ensemble of conformational states. **f**, Negative stain transmission electron microscopy validation: class averages were postprocessed and matched to projection images of ORF2p models. **g**, Localization densities represent the structural flexibility of EN, tower, wrist and CTD domains in the ensemble of full-length ORF2p models. Representative full-length ORF2p models from the validated ensemble highlight concerted movements of EN, tower and CTD relative to fingers, palm and thumb, together allowing ORF2p to adopt open and closed states. Data in **a**, **b** and **e** are representative of two independent experiments and shown as mean ± s.d.

**Extended Data Fig. 7:**
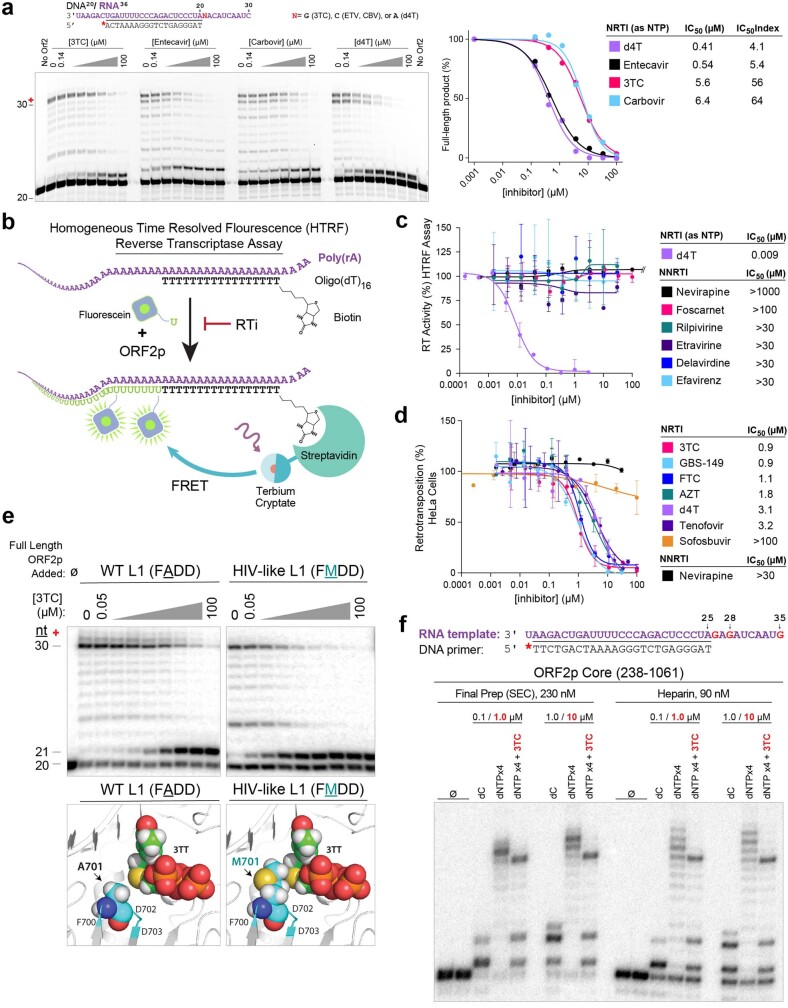
Inhibition of ORF2p core by NRTI and NNRTI reverse transcriptase inhibitors. **a**, NRTIs are inhibitors of the RT activity of ORF2p core. Denaturing PAGE migration pattern (left) of RT reactions inhibited by NRTIs and their quantification (right) indicate NRTIs are potent (low µM IC_50_) inhibitors of ORF2p core. ORF2p core was preincubated with a template:primer containing a single site for the incorporation of a given nucleotide analogue. The primer/template sequence shown in the panel **a** illustrates the case of single incorporation of 3TC, with a single G for incorporation; the incoming template sequence for entecavir and carbovir has a single C, and for d4T a single A, each at position labeled “N”. Reactions were incubated for one minute at 37 °C with a 100 nM dNTP mixture and increasing concentrations of listed inhibitors. IC_50_ index, fold = IC_50_ (drug, µM) ÷ [dNTP] (natural counterpart, µM, here 0.1 µM) and reflects the fold-excess of a required NRTI over its natural counterpart to give a 50% inhibition in DNA synthesis. **b**, Schematic of homogenous time-resolved FRET RT (HTRF) assay. Fluorescein-labeled dNTPs (here, uracil-TP) are incorporated by ORF2p into a biotinylated primer, here shown against a poly(A) template. Detection is then achieved using FRET with a terbium cryptate labeled streptavidin, and the time-resolved technique and time-delayed emission from terbium cryptate reduces background from other fluorescent chemicals in the mix. In the presence of ORF2p RT inhibitors (RTIs), base incorporation is stopped and FRET signal is lost. For NNRTIs the indicated poly(A)-oligo(dT) template:primer is used; for NRTIs, a template:primer pair of RNA_36_:biotin-DNA_25_ is used. **c**, Quantification of HTRF screen shows HIV NNRTIs do not inhibit ORF2p, even at concentrations up to 1 mM for nevirapine. Upon binding of NNRTIs, such as nevirapine, the primer grip and the 94–102 segment shift which, together with movement of Y181 and Y188, open the NNRTI pocket. Accordingly, mutations of the 94–102 segment, Y181 and Y188 have all been implicated in resistance to multiple NNRTIs^65–72^; n = 3 independent wells representative of two independent experiments. **d**, Inhibition assay in HeLa cells stably expressing a dual luciferase L1 retrotransposition reporter^73^, normalized to cell viability using Cell Titer Glow reagent; n = 3 biologically independent wells representative of two experiments. **e**, 3TC analog in the context of the native FADD active site loop (left) and 3TC analog in the context of the mutant FMDD active site loop (right). Note the lack of van der Waals contacts between the Ala 701 side chain and the oxathiolane ring, including the sulfur atom, in the nucleotide in the native active site, contrasted with the favorable contact between the Met 701 and the oxathiolane ring in the nucleotide. Similar effects were shown previously in the YADD mutant of HIV (WT YMDD)^74^. **f**, Full length ORF2p and ORF2p core are compared in single nucleotide incorporation and inhibition experiments with the indicated nucleoside triphosphates and 3TC triphosphate; ‘dNTPx4’ is a mix of all four standard dNTPs. Full length ORF2p (purity insufficient to accurately determine concentration) produces similar reaction products and shows similar activity and inhibition to both partially-purified (Heparin) and fully-purified (after SEC) ORF2p core. This assay qualitatively reveals both incorporation and tolerance for some misincorporations of the polymerase. For example, in the ‘dC’ lane, containing only dCTP, the 26 nt band represents one C-G incorporation, and the 28 nt band is from subsequent C-A misincorporation followed by a C-G incorporation. Adding 3TC chain-terminates products and the strong bands at at 26, 28, and 35 nt highlight the G-base positions in the template.

A critical path towards treating diseases associated with RT activity, such as HIV and HBV infections, is the use of RTIs^40^. Given the emerging role of L1 in disease, we sought to determine whether current RTIs had activity against ORF2p. Titrating nucleoside triphosphate (NTP) forms of nucleoside RTIs (NRTIs) into gel-based L1 RT assays showed that 3TC (lamivudine, Extended Data Fig. 7a) and carbovir (the active metabolite of abacavir) were modest ORF2p inhibitors (half-maximal inhibitory concentration (IC_50_) 5–7 µM), whereas d4T (stavudine) and entecavir were more potent (IC_50_ 0.4–0.6 µM, Extended Data Fig. 7a). To enable robust high-throughput inhibition analysis, we developed homogeneous time-resolved fluorescence assays for ORF2p RT. NRTI NTPs all inhibited ORF2p to varying extents, with thymidine analogues dideoxythymidine (ddT) and d4T the most potent (IC_50_ < 10 nM), followed by AZT and 3TC as modest inhibitors under these conditions (IC_50_ 200–750 nM)^33^ (Fig. 4a and Extended Data Fig. 7b,c). By contrast, none of the six tested allosteric HIV-1 non-nucleoside RTIs (NNRTIs) inhibited ORF2p; notably, even 1 mM nevirapine showed no inhibition (Fig. 4a, Extended Data Fig. 7c and Supplementary Fig. 6a,b). Using a stable dual luciferase retrotransposition reporter system in HeLa cells, we confirmed previously published modest inhibition of L1 by d4T, 3TC, FTC (emtricitabine), AZT, tenofovir and GBS-149 (IC_50_ 1–5 µM)^33^ (Extended Data Fig. 7d). GBS-149 potency was not significantly different from that of related 3TC and FTC; the HCV inhibitor sofosbuvir did not inhibit L1 at up to 30 µM (Extended Data Fig. 7d). Differences between the in vitro and cell-based assays may be attributable to differential triphosphorylation of NRTIs.

## Structural basis of inhibition of ORF2p

Potency against ORF2p varied almost 200-fold between NRTIs tested, and AZT and 3TC were not potent inhibitors (Fig. 4a). In HIV-1, resistance to 3TC can come from M184 mutations in RT (YMDD to YVDD/YIDD), which cause a steric clash with the oxathiolane ring^47^. HIV-1 mutants to Ala (YADD, like FADD in ORF2p) have been studied with respect to 3TC potency, demonstrating that van der Waals interactions between M184 and the 3TC oxathiolane ring are stabilizing; these interactions are not present with the smaller A701 (FADD) in ORF2p, and this difference may explain the relatively lower potency of 3TC against L1 ORF2p RT. Modelling the related 3TT-TP analogue into the active site of L1 using the cocrystal structure of dTTP confirmed the proximity of M701 to the oxathiolane ring, whereas the A701 in wild-type L1 was further away. Further supporting this mode of inhibition, 3TC was approximately 15-fold more potent in inhibiting A701M mutant full-length ORF2p (FMDD) than wild type (FADD, Fig. 4b and Extended Data Fig. 7e). On the basis of these results, HIV-1 inhibition^40^ and analyses of HERV-K^34^, we conclude that 3TC and related FTC and GBS-149 are unlikely to be selective for L1 ORF2p.

To understand the structural basis underlying differences between AZT and more potent thymidine analogues, we modelled the triphosphates of thymidine-based NRTIs into the ORF2p ternary crystal structure containing dTTP in the N-site. As expected, ddTTP and d4T-TP did not show any clashes with the protein, as they closely resemble the shape of dTTP. However, the AZT-TP model showed a clash of the middle nitrogen of the 3′-azido group with amide hydrogen of F605 (distance 2.03 Å, Fig. 4c), which was not relieved by energy minimization. This clash was not observed in the crystal structure of AZT-TP bound to HIV-1 RT (respective distance 2.28 Å, Fig. 4c). The inability to remove the clash in ORF2p may be explained by a difference in conformational flexibility of the region around the 3′-azido group (residues 602–607 in ORF2p and 112–117 in HIV-1 RT). In ORF2p, this segment contains two internal salt bridges that are absent from HIV-1 RT and has lower average backbone *B* factors than HIV-1 with respect to the complete dNTP site (defined as all residues within 6 Å of dTTP; site versus region in ORF2p, 43.4 versus 48.1; HIV-1 RT, 114.3 versus 110.7). Calculations on the basis of free energy perturbation simulations of the relative ORF2p binding of these nucleotides showed an insignificant difference in relative binding free energy (ΔG) between ddTTP and d4T, but a large positive difference between these and AZT (Supplementary Fig. 6c), consistent with the greater than 20-fold change in ORF2p inhibitory activity of AZT compared with ddTTP and d4T (Fig. 4a).

As inhibition of telomerase RT (TERT) would be a potential source of toxicity in a therapy, we investigated the relative selectivity of NRTI triphosphates for L1 versus TERT, testing the panel of NRTI triphosphates in a biochemical TERT assay. The tested compounds were generally around 1,000-fold less potent inhibitors of TERT than L1 RT, with IC_50_ in the mid-micromolar range (for example, the IC_50_ of d4T-TP was 9 nM versus ORF2p and 15 µM versus TERT; Supplementary Fig. 7a); this result was in line with expectations, because these drugs are all tolerated therapeutically in patients. The structures of the active sites of the two enzymes explain these stark differences, with a more hydrophobic environment in the ORF2p active site (Supplementary Fig. 7b,c). NRTIs designed for HCV RdRp are also unlikely to inhibit L1 as drugs of this class, like sofosbuvir, contain 2′ modifications mimicking the 2′-OH of an incoming ribonucleoside triphosphate. This was first confirmed by modelling of sofosbuvir into the ORF2p active site, which revealed a clash between the sofosbuvir 2′ F and the gatekeeping residue F605; this was further confirmed in cell-based L1 assays, which showed no inhibition by sofosbuvir (Extended Data Fig. 7d and Supplementary Fig. 7d). Together, these results demonstrate that the ORF2p crystal structure provides a useful starting point for structure-based design of new ORF2p-specific NRTIs.

NRTIs act at the RT active site and are known to inhibit ORF2p with varying potency, whereas HIV-1 NNRTIs^33^ bind to an induced allosteric site in the palm between the primer grip, the β-sheet containing the YMDD loop and the 94–102 segment^40^; this pocket is absent from HBV, HIV-2 and HERV-K^34^. HIV-1 NNRTIs do not inhibit ORF2p (Fig. 4a and Extended Data Fig. 7c,d), and structural and sequence differences between the HIV-1 NNRTI pocket and the equivalent region in ORF2p explain this lack of inhibition (Fig. 4d). As HIV-1 RT undergoes a conformational change when NNRTIs bind, the HIV-1 RT structure in the absence of NNRTI was compared with the ORF2p crystal structure. The most striking difference was replacement of the 94–102 segment of HIV-1 RT with a longer α-helix formed by residues 572–588 in ORF2p, making none of these positions structurally equivalent. In addition, residues Y181 and Y188, which have been implicated in aromatic ring stacking with nevirapine and other NNRTIs^40^, were replaced with S698 and I705, respectively, and the small residue G190 in HIV-1 RT was replaced with bulky Y707 in ORF2p. These differences, taken together, explain why ORF2p does not form a pocket that binds HIV-1 NNRTIs.

## Structure of full-length ORF2p

Purified full-length ORF2p was similarly active to the ORF2p core in single-nucleotide-resolution RT assays and was similarly inhibited by 3TC (Fig. 4e, Extended Data Fig. 7f and Supplementary Fig. 8a–c), indicating that EN and CTD may not directly modulate RT activity. Monodisperse full-length ORF2p, bound to the same short RNA_17_–DNA_14_ hybrid used above for cryo-EM of the ORF2p core, was analysed by negative stain transmission electron microscopy and found to be monomeric and probably flexible, with two-dimensional classes indicating multiple conformations (Fig. 4f, raw contour, and Supplementary Figs. 9–10). To elucidate the conformational landscape of ORF2p, we used cryo-EM maps, cross-linking mass spectrometry, AlphaFold2 and molecular dynamics simulations to generate an ensemble of conformational states using the Integrative Modeling Platform (Supplementary Figs. 8d,e, 9 and 10 and Supplementary Tables 1 and 2). Informed by AlphaFold2 and molecular dynamics simulations, we first segmented the EN, tower and CTD into 15 rigid bodies connected by 14 flexible linkers and computed an ensemble of integrative models satisfying the input data (Fig. 4g; conformational heterogeneity and model uncertainty is represented as localization densities). The ensemble was then validated by matching computed two-dimensional model projections to negative stain two-dimensional class averages: each class average was assigned a best-matching model and each matched model fit the data better than the parental AlphaFold model (Fig. 4f and Supplementary Fig. 10). Structural clustering of these best-matching models indicated two distinct groups (Fig. 4g and Supplementary Fig. 10), which we named ORF2p open and closed-ring states, that were characterized by unique positions of the EN and tower. Closure of the ring entailed an approximately 48 Å movement of the tower domain (measured from the top of the tower), hinging at the baseplate and bringing it adjacent to the CTD. To test potential roles of these states, we repeated the negative stain EM with ORF2p bound instead to a 376 nt RNA derived from the 3′ end of L1RP with a 14 A tail. Many classes overlapped, but there was also a significantly increased number of closed-ring states and a reduction in open states (Supplementary Fig. 10b–d). We interpret these differences to mean that the closed state may represent a predominant conformation when ORF2p is bound to messenger RNA, whereas the open state may be involved in retrotransposition.

## Domain comparison of ORF2p and other RTs

**Extended Data Fig. 8:**
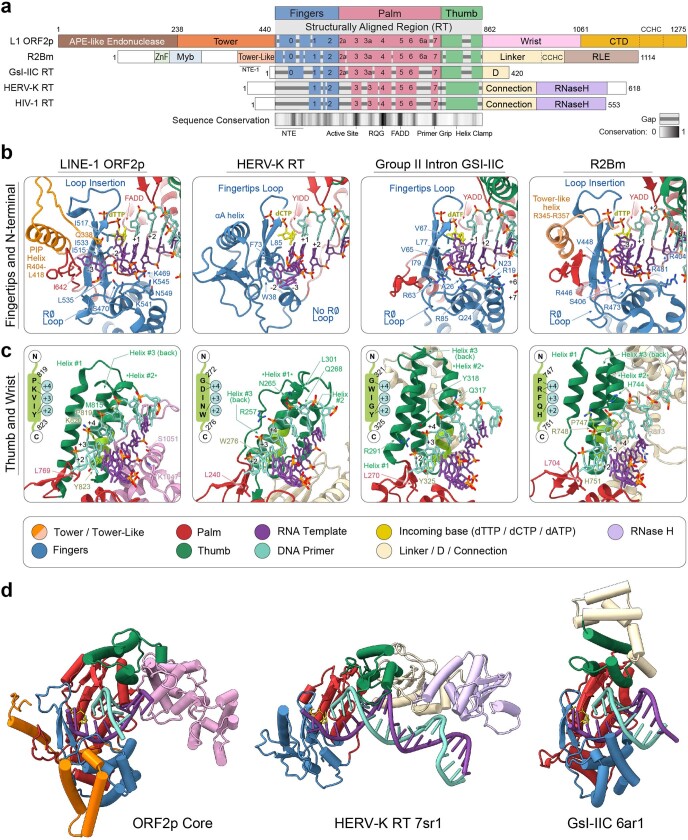
Comparison of ORF2p with other RTs. **a**, Domain organization and sequence alignment of LINE-1 ORF2 (L1RP locus, GenBank AF148856) with other reverse transcriptase (RT) containing proteins: Bombyx mori R2Bm RT (PDB 8gh6, GenBank AAB59214), group IIC intron (PDB 6ar1, Uniprot E2GM63), non-LTR element HERV-K (PDB 7sr6, clone 10.9, GenBank AF080231), and retrovirus HIV-1 RT (PDB 4pqu, UniProt: P03366.3). Sequences were aligned structurally using ChimeraX software and via the conserved RT sequence blocks (0–7)^75,76^, with degree of sequence conservation and common structural features noted below. **b**, Comparison of the N-terminal extension and 5’ template contacts between (from left to right) LINE-1 ORF2p, HERV-K RT (PDB 7sr6), group IIC intron (PDB 6ar1) and R2Bm (PDB 8gh6). The ORF2p PIP box helix occupies the space of the HIV αA helix and a tower-like helix in R2Bm that is not a PIP box. The template makes extensive contacts with ORF2p and takes a distinct 5’ path upstream of the active site than in the other RTs, guided by adaptations in fingers (L535), palm (I642), and tower (Q338). **c**, Comparison of downstream primer-binding surfaces across the four RTs; primer contacts with thumb helix clamps (lime green) shown inset. The thumb in ORF2, R2Bm, and GSI-IIC is permuted relative to HERV-K (and HIV), with the primer-contacting helix clamp on helix #2, whereas it is on helix #1 in HERV-K. ORF2p wrist also contacts the template, the R2 linker makes a smaller set of contacts, and these bases are exposed in HERV-K and GSI-IIC. **d**, Models of ORF2p Core, HERV-K RT, and GSI-IIC RT^77^ aligned by palm superposition. The RT domains of GSI-IIC and ORF2p Core are more similar to each other than to HERV-K. The HERV-K linker and RNase H domains occupy a similar position to the ORF2p wrist, and GSI-IIC D domain is in a similar position to ORF2p CTD and R2 CCHC (see Supplementary Fig. 11).

To better understand specific adaptations of ORF2p, we compared it with diverse structurally characterized RTs: the R2 LINE element from the silk moth *Bombyx mori* (R2Bm)^22^, the distantly related mobile group IIC intron RT from *Geobacillus stearothermophilus* (GsI-IIC)^30^, the RT from LTR element HERV-K^34^ and HIV-1 RT (Extended Data Fig. 8a). The structure of the group IIC intron was chosen over the evolutionarily closer group IIB intron^31^ because it represents the same active form with substrate in the active site and is higher resolution, although members of the IIB family were included in the wider evolutionary analysis (see below). ORF2p is larger than the other enzymes, with limited similarity outside the conserved right-hand fingers–palm–thumb subdomains in RTs. Structural alignment of all five enzymes by palm superposition highlighted conserved RT sequence blocks and showed that ORF2p had insertions in fingers (motifs 0, 2a) and palm (motif 3a, 6a) and permutation of the thumb helices compared with both HIV-1 and HERV-K.

**Extended Data Fig. 9:**
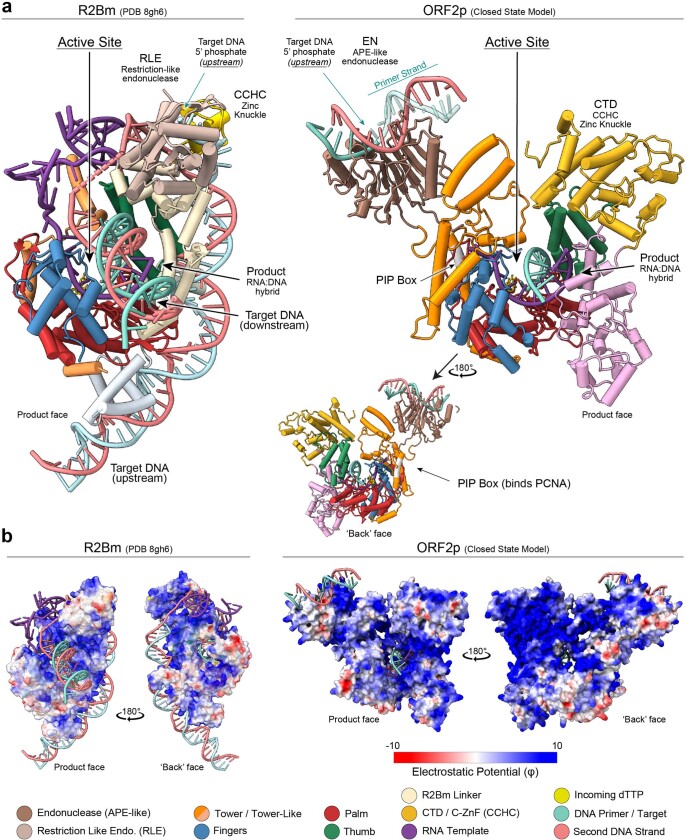
ORF2p and R2Bm structures show opposing topologies of target DNA relative to the active site. **a**, Comparison of ORF2p and R2BM structures, oriented identically following palm superposition; the closed state (Class 15) ORF2p model is shown. In both structures, the active site is in back center (incoming dTTP is visible) and generated product would be ratcheted out of the enzyme by sequential base additions, pulling template RNA through as the product emerges towards the viewer out of the plane of the printed page. In R2Bm, resolved initiating TPRT, the C-terminal restriction-like endonuclease (RLE) holds the 5’ phosphate from the upstream target DNA in the ‘top right’, as viewed here from the product face, and the adjacent CCHC zinc knuckle melts the target strand from second strand, allowing the upstream target DNA to wrap around the positively charged ‘back’ face. In contrast, ORF2p has an N-terminal APE-like endonuclease (EN, from PDB 7n8s^50^; primer strand with 3’-OH is transparent), located on the opposite wall of the polymerase groove relative to the position of RLE in R2. However, the CTD CCHC remains in the ‘top right’, positioning the CCHC zinc knuckle nearly identically in both enzymes. To summarize, R2 has both RLE and CCHC together, on the ‘top right’ of the active site, whereas in ORF2p, EN and CTD are on opposite sides of the active site, with EN on the ‘top left’ and CCHC on the ‘top right’, and in this configuration, the target DNA would traverse across the two domains. Indeed, because the primer (bound to downstream DNA) must similarly be passed into the active site, the apparent result of this is that the target DNA is reversed in ORF2 with respect to R2: the downstream DNA would most likely similarly bind the CTD CCHC zinc knuckle and wrap around the highly positively charged ‘back’ face of the enzyme, similar to the behavior of the upstream DNA in R2. A cartoon of this is drawn in Fig. 6b. However, other orientations are possible, and these models were resolved without EN-bound DNA. **b**, Calculated Coulombic potential mapped onto the model surfaces (ChimeraX) shows extensive positively charged surfaces on both R2Bm and ORF2p. In R2Bm, resolved starting TPRT, target DNA and the structured RNA bind to most of the positively charged (blue) surface, which includes specific domains that recognize the unique sequences and structures of the target ribosomal DNA and 3’ untranslated region (UTR) of the R2 RNA. On ORF2p, the ‘back’ face of the enzyme is extensively positively charged, and these surfaces are highly likely to be involved in binding both target DNA and template RNA. These charged residues are largely required for retrotransposition^38^ and may coordinate a similar path of the target DNA in ORF2p as in R2. The DNA clamp ring PCNA binds to the ORF2p PIP box during integration on the ‘back’ face (gray helix, arrows), and it appears that PCNA could be loaded on the target DNA if it were to wrap this positively charged ‘back’ surface (Fig. 6b). R2 does not have a PIP box and PCNA has no known role in R2 mobile element insertion.

Viral and LTR transposon RTs, represented by HIV-1 and HERV-K, are distinct from the non-LTR RTs in that they encode their own RNase H, located C-terminally, and GsI-IIC has a DNA-binding D domain in this position (Extended Data Fig. 8c,d and Supplementary Fig. 11). Other than GsI-IIC D, these CTDs all stabilize the polymerase complex by coordinating downstream nucleic acids but do so in distinct ways. The ORF2p wrist binds the template close to the active site; the connection and RNase H domains of viral/LTR elements bind distally; and, although the linker of R2Bm makes limited and distinct nucleic acid contacts, most of its function seems to be coordination of the activity of the C-terminal RLE domain^22,48^. In R2Bm, RLE cuts ssDNA, which in the context of initiation is melted from the dsDNA target by the adjacent C-terminal CCHC zinc finger (ZnF)^22,24,48^. The ORF2p CTD is required for retrotransposition^37,38^ and has a similarly positioned CCHC motif (Extended Data Fig. 9 and Supplementary Fig. 11) that may also melt target DNA and/or bind single-stranded nucleic acid^49^, but its function remains unclear.

In comparison with R2Bm, the ORF2p domain topology is reversed: ORF2p apurinic/apyrimidinic endonuclease (APE)-like EN is located N-terminally and cuts dsDNA rather than ssDNA^12,13,22,50^. Structurally, ORF2p EN sits on the opposite wall of the polymerase groove to R2Bm RLE, atop fingers rather than thumb (Extended Data Fig. 9 and Supplementary Fig. 11). This seems to position the target DNA in reverse orientation to the active site for the two enzymes, although other orientations are possible (Extended Data Fig. 9). The tower of ORF2p seems to play a part in dynamic positioning of the EN. A smaller domain that we term ‘tower-like’ is present in R2 (residues 305–374); this region was previously annotated as NTE-1 and contains the tower lock as well as helices analogous to ORF2p PIP that anchor the tower lock to fingers and palm. However, the PIP box, tower and tower baseplate are not present in R2. R2Bm also has two N-terminal domains, Myb and N-ZnF, that recognize specific ribosomal DNA sequences unique to the element, reflecting the extremely high sequence specificity of R2 for a single site in the ribosomal DNA.

## Structural adaptations of ORF2p RT

There are numerous contrasting features of the N-terminal regions of the four RT families (Extended Data Fig. 8b). Viral and LTR RTs have an α-helix posterior to the fingertips, which is absent from the group II intron RT but occupied by the tower-like helix of R2Bm and the PIP helix in ORF2p. The fingertips of all four representative RTs are similar in that they provide a hydrophobic surface for sliding the template bases (notably I515, I517 and I533 in ORF2p), but ORF2p and R2Bm both have a distinctive insertion in the fingertips loop. The upstream template path differs significantly in all four enzymes: in viral and LTR RTs, the 5′ template is pushed away from the fingertips by π-stacking with a characteristic tryptophan (W38 HERV-K, W24 HIV-1), whereas the non-LTR transposons and group II intron have a groove formed by the conserved R0 region with a loop that forms a lid for the template. Here, ORF2p is also distinct: the fingertips for group II intron and R2Bm have an arginine (R63 and R446, respectively) that forms a salt bridge with the *n*_−2_ phosphate, pushing the *n*_−3_ base away from the posterior side of the fingertips, whereas the analogous residue in ORF2p (T638) is significantly smaller and allows the *n*_−3_ base to fold into a hydrophobic pocket created by a loop from the palm anchored by I642. The result of this is an apparently different entry path of the template RNA. The R0 region also differs significantly between ORF2p and the group II intron and R2Bm: the R0 loop in ORF2p is the longest of the three and makes no primer contacts; by contrast, the group II intron and R2Bm both contact the *n*_+6_ primer backbone.

In these RT families, the proximal primer is anchored by a conserved primer grip in the palm, which contains a characteristic hydrophobic motif helix clamp (Extended Data Fig. 8c). C-terminal to the primer grip is the thumb domain, a parallel three-helix bundle that occupies the minor groove of the template–primer heteroduplex and makes extensive primer contacts. The thumb in LTR RTs is permuted relative to the other families: the second helix of ORF2p, R2Bm, and the group II intron is functionally analogous to the first α-helix in viral and LTR RTs and contains the helix clamp subdomain at its base^30^ (Extended Data Fig. 8c). The helix clamp proline in non-LTR RTs (P819 in ORF2p) assumes a similar function to the glycine in LTR RTs and the group II intron, allowing proximity to the minor groove, and the subsequent aromatic residue (Y823 in ORF2p) forms π-interactions with the primer *n*_+2_ or *n*_+3_ nucleotide backbone. The wrist of ORF2p makes more extensive contacts with the downstream template than either the group II intron D domain or the R2Bm linker.

## Structural insight into L1 evolution

**Fig. 5:**
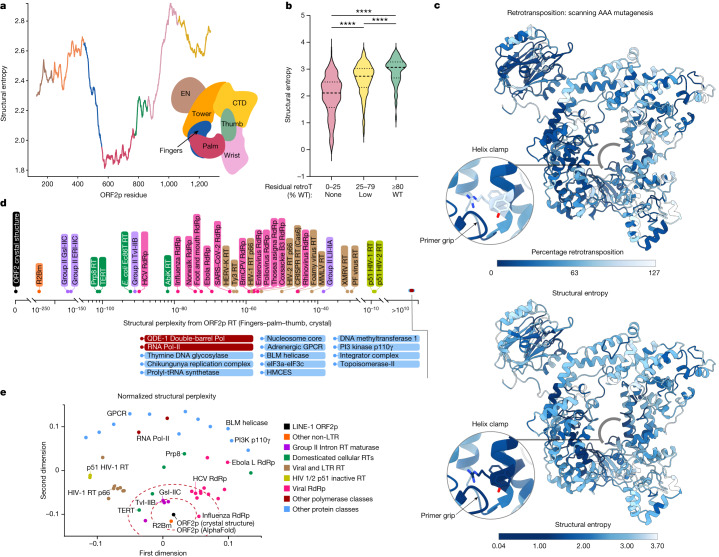
Structural evolutionary analysis of ORF2p. **a**, Structural Shannon entropy (‘structural entropy’) in ORF2p, measured from 57 L1 sequences from diverse vertebrates and plants and smoothed by averaging a 130-residue (approximately 10% of protein length) sliding window was lowest in the ancestral palm domain and highest in the C-terminal domain. **b**, Structural entropy correlates strongly with retrotransposition (retroT, ****P < 10^−15^, two-tailed *t*-test), comparing with retroT measurements from 417 consecutive scanning trialanine mutants of ORF2p^38^. **c**, Mapping retroT and structural entropy onto the structure of ORF2p highlighted the overall concordance, as well as a notable discordance in the helix clamp around residue Y823 (inset). **d**, Structural perplexity, an information-theoretic measurement of the structural distance between two proteins, relative to ORF2p RT of a curated set of 50 proteins calculated using Plexy (Supplementary Methods). **e**, Normalized structural perplexity between full-length ORF2p and all proteins in the curated set, represented using multidimensional scaling such that the relative pairwise Euclidean distances were preserved (Supplementary Methods). For RT and RT-like proteins, the polypeptide with polymerase activity is used; for other proteins, the entire biological assembly is used. Dashed red lines represent the first and second standard deviations of the two-dimensional distance from full-length ORF2p. 2D, two-dimensional.

L1 dates to at least the Precambrian era^51^; on the basis of limited sequence similarity, it is speculated to have a putative common ancestor with bacterial mobile group II introns^51^ and has no clear evolutionary ancestor among extant viruses. We therefore sought to use protein structure to shed light on the conserved features and evolutionary origin of ORF2p that cannot be identified by sequence alignment alone. We used multiple sequence/structural alignments and AlphaFold2 predictions to examine conservation of the human ORF2p structure relative to 57 other L1 ORF2p sequences from vertebrates and plants. By computing and plotting the residue-level diversity of the aligned ORF2ps as the Shannon entropy (Fig. 5a and Supplementary Methods), we found high concordance between the two multiple alignment strategies (sequence versus structural) in the RT domain (fingers–palm–thumb, Supplementary Fig. 12a). Despite relatively lower sequence conservation in regions of the tower, wrist and CTD domains, the structure was conserved, indicating that domain topology may be more important than the sequence of these domains for L1 function. Leveraging data from a published trialanine mutagenesis library of 417 consecutive AAA ORF2p mutants, in which residual function of mutants was compared with that of the wild type (100%)^38^, we found that structural entropy was significantly correlated with residues dispensable for retrotransposition activity (Fig. 5b,c and Supplementary Fig. 12a). As most mutations resulted in reduced function, these results together indicate that optimization of retrotransposition is a main evolutionary driving force.

We next compared ORF2p and other proteins with the intention of identifying shared structural features and inferring evolutionary relationships. First, we manually curated a set of 50 experimental protein structures that represented main families: RTs, RdRps, DdDps (DNA-dependent DNA polymerases) and DdDps/RdRps, as well as ‘negative controls’ that should have little resemblance to the other proteins (Supplementary Table 3). We then sought to represent structural similarity in a manner that would faithfully account for differences in protein length, account for inherent alignment quantity/quality trade-offs, and address a limitation of other methods, such as RMSD, in which different relative orientations of otherwise identical domains result in poor scores. We developed a new information-theoretic algorithm, named ‘Plexy’, which represents a high-quality alignment as one that reduces the structural perplexity between their coordinates (Supplementary Methods). The smaller this value, the more likely it is that one can ‘guess’ the coordinates of one structure knowing the coordinates of the other. Plotting structural perplexity from ORF2p RT for this set (Fig. 5d and Supplementary Figs. 12b,c and 13) showed that it recapitulates close relationships between ORF2p, R2Bm and group II introns, and that ‘negative control’ proteins have extremely high perplexities from ORF2p. To better understand relationships between full-length ORF2p and other proteins, we computed the pairwise structural distances across all pairs of proteins and normalized them with respect to the size of the two proteins and their alignment, anchoring the plot on the ORF2p crystal structure (Supplementary Methods, Fig. 5e). Across both datasets, proteins in the same functional class typically clustered together in an unsupervised manner, with R2Bm and group II introns again closest to ORF2p. Group IIB introns are thought to be evolutionarily closer to L1 than group IIC, but intriguingly both have similar perplexities from ORF2p with subtle differences in subdomains, highlighting structural conservation (Supplementary Fig. 13). Domesticated cellular RTs were next closest to ORF2p RT, but normalized distances between full-length ORF2p and Prp8 and TERT were larger owing to the incorporation of unrelated structural elements (Supplementary Fig. 12b). Viral RdRps such as HCV and influenza B have remarkable similarity to ORF2p RT^30^; non-LTR and viral RTs are more distant. Notably, the inactive p51 HIV-1/2 RT subunit was predicted to be far more distant to ORF2p than the active p66 HIV-1/2 RT, despite identical amino acid sequence (up to a deletion). Therefore, this analytical framework quantifies conformational similarity in a manner that is sensitive to function.

## Discussion

Our integrated analyses reveal the inner workings of the molecular machine that has written nearly half of the human genome. Understanding L1 structure and function is important both in evolution and, increasingly, in human disease. Accumulating evidence links L1 activity and the host response to common pathologies including cancer, ageing, neurodegeneration and autoimmunity^2–7,26,27^. Our biochemical, structural and evolutionary analyses show that ORF2p contains a highly active polymerase that is uniquely adapted for its parasitic replication cycle, with both conserved and new structural features that preserve optimal retrotransposition throughout evolution. Together, these data provide insights into two key underlying mechanisms through which L1 may cause disease: (1) nuclear insertional mutagenesis and resultant genomic havoc, and (2) cytosolic sensing of the products of ORF2p reverse transcription.

**Fig. 6:**
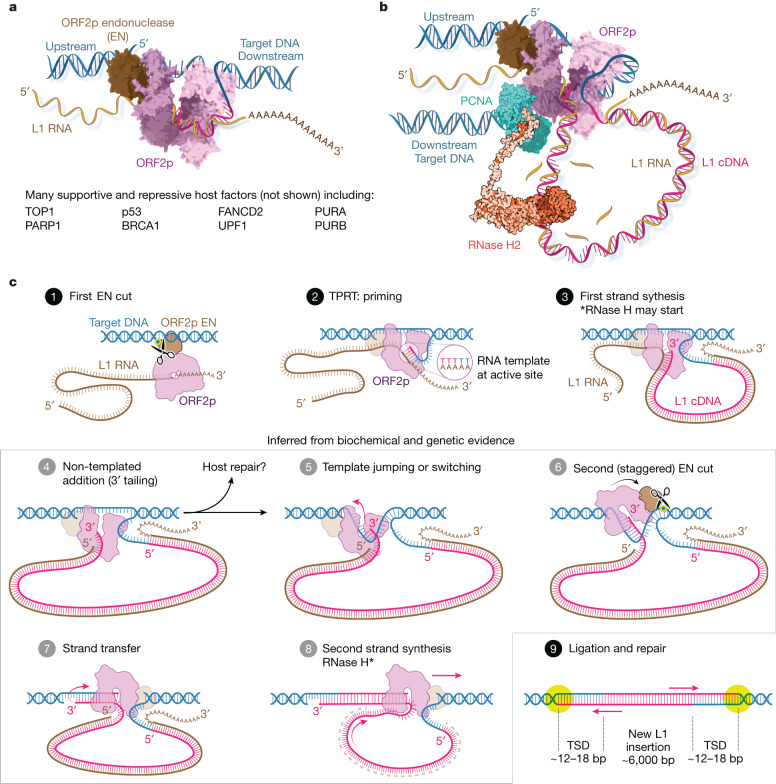
Revised L1 insertion model. **a**, ORF2p bound to target DNA as TPRT begins, drawn schematically with linear target DNA for clarity as in the models below. **b**, ORF2p in complex during first strand synthesis. It seems more likely that ORF2p bends the target DNA around the highly positively charged ‘back’ face of the polymerase (Extended Data Fig. 9); it can then pass through the PCNA ring clamp, which binds to the PIP box and recruits RNase H2 (ref. ^29^). **c**, Revised insertion model. Activities supporting steps 4, 5, 7 and 8 are demonstrated here. 1. ORF2p EN cuts target DNA, liberating a gDNA 3′-OH 2. TPRT: the T-rich gDNA primer is passed into the RT active site, where it base pairs with the poly(A) tail of the bound template, and the 3′-OH is extended. 3. First strand synthesis generates a large (6 kb) cDNA loop; RNase H2, recruited by ORF2p–PCNA, can begin. 4. NTA, in which extra bases are added to the 3′ cDNA end beyond the 5′ end of the RNA template, may occur. 5. Template jumping or switching to the exposed single-stranded gDNA may follow, potentially facilitated by microhomology from NTA nucleotides and the 5′ cap. This would also release 5′ phosphate-bound EN to ‘rock and roll’^20,24,48^ to carry out: 6. The second EN (staggered) cut, which liberates the 3′ OH used to prime second strand synthesis; a stagger from the first cut of approximately 12–18 bp results in characteristic target site duplications (TSDs)^20,21,24,44^. 7. Strand transfer and priming of second strand synthesis. 8. Second strand synthesis using the 6 kb L1 cDNA as template. RNase H2 activity may also occur here. 9. Ligation and end repair, resulting in a completed approximately 6 kb insertion flanked by TSDs. The second EN cleavage may sometimes occur in the absence of a template jump. **b**, © 2023 JHUAAM. Illustration: Jennifer E. Fairman.

Although nuclear L1 activity has been correlated with DNA damage and structural genomic rearrangements^2,41,42,52^, a mechanistic understanding of L1 insertion has been elusive. The insertion process can be understood as two half reactions: first and second strand synthesis. Second strand synthesis has been challenging to study, and it was unclear whether it is performed by L1 or the host. Our data demonstrate that ORF2p is competent to perform all enzymatic steps required to prime and execute both first and second strand syntheses: it effectively synthesizes DNA with short RNA or DNA primers on both RNA and DNA templates (Fig. 3, Extended Data Figs. 4 and 5 and Supplementary Figs. 3–5). Interpreting our results in the context of high-quality biochemical data from decades of studying the R2 LINEs in insects^21,24,36,48^ provides us with the opportunity to update the L1 insertion model (Fig. 6). The mechanism describes a canonical insertion that is intentionally simplified and omits numerous supportive and repressive host proteins, including topoisomerase TOP1, PARP1, purine-rich element binding proteins, the Fanconi pathway (including BRCA1) and p53 (refs. ^8,17–19^). Furthermore, alternative pathways as such host-catalysed second strand synthesis may occur in different contexts or following ORF2p failure, and the host may combat insertion by, for example, cleaving intermediates.

Our data also shed light on other areas of the canonical L1 replication cycle. ORF2p *cis* RNA binding is thought to occur at the ribosome^53,54^. Newly translated *apo* ORF2p is unstable until RNA is bound, and it assumes a ‘thumb up’ conformation competent to tightly bind RNA; we speculate that the initial RNA binding probably occurs cotranslationally, potentially before the CTD has even been translated. PCNA binding, which is required for retrotransposition^17^ and recruits RNase H2 to allow second strand cleavage^29^, does not seem to be occluded in any identified state; this, together with EN and RT dependence^17,18^, indicates that PCNA may be recruited to ORF2p by the developing genomic lesion. Most new LINE insertions are heavily 5′ truncated^1^; often they comprise only a few hundred base pairs, but the reasons are not well understood. ORF2p is efficient and highly processive, consistent with previous observations^16,32^, adding support to the idea that host cleavage of the L1 RNA or intermediates is more likely to cause 5′ truncation than inefficiency of the polymerase^55^. Nuclear ORF1p levels are limited^17,18^, and bound ORF1p chaperones would be displaced from L1 RNA during RT, potentially leaving the large single-stranded cDNA loop intermediate unprotected (steps 3–7, Fig. 6). This could represent both a unique vulnerability and a potential nidus for translocations^41,42,52^, given its homology to much of the genome.

Cytosolic double-stranded nucleic acids, viral mimicry and resultant interferon signalling are known to contribute to pathology in several contexts, and NRTIs have been shown to limit the production of interferon and of these nucleic acids^6,7^, but their origin has remained controversial. First, our data show that ORF2p can use RNA primers and short RNA hairpins to initiate RT reactions; an *Alu*-like sequence is readily extended, and uridylation of the L1 RNA^56^ might convert it into a similar substrate as well. RNA priming of ORF2p RT in the cytoplasm can parsimoniously explain the origin of these nucleic acids. We also show that DNA primers as short as 5 nt can prime L1; it is possible that shorter primers are also tolerated^16^. Second, we demonstrate that L1 can directly synthesize RNA:DNA hybrids in the cytosol; these are RT-dependent but EN-independent, ruling out a nuclear origin in this system. Third, we show that L1 synthesized cDNAs activate cGAS/STING, resulting in interferon production. Our observations further demonstrate the potentially critical role of L1 and its RT products in viral mimicry^57,58^, as inferred from genome and cancer evolution^59,60^. Moreover, our robust inhibitor data provide a framework for evaluating the involvement of L1 in these phenotypes and for targeting this in the future. In summary, our structural elucidation of ORF2p will facilitate rational design of new therapeutics and lays the groundwork for future studies needed to dissect and improve our understanding of the insertion mechanism of L1, its evolution and its roles in disease.

### Reporting summary

Further information on research design is available in the Nature Portfolio Reporting Summary linked to this article.

## Online content

Any methods, additional references, Nature Portfolio reporting summaries, source data, extended data, supplementary information, acknowledgements, peer review information; details of author contributions and competing interests; and statements of data and code availability are available at 10.1038/s41586-023-06947-z.

### Supplementary information

Supplementary Information Supplementary background and discussion: further L1 background, relation to disease and mechanistic information; Methods; Figs. 1–13: overview of cryo-EM and further biochemical, modelling, and structural information and comparisons; Tables 1–5: summary of evolutionary and modelling datasets, plasmids and affinity reagents; and references.

Reporting Summary

Supplementary Data Gel source data.

Supplementary Video The human genome contains around half a million copies of a virus-like element known as L1. In certain disease states, L1 escapes repression and generates RNA molecules that migrate to the cytosol, encoding proteins that can transfer L1 RNA back to the nucleus, where, through reverse transcription, they can create and insert a new L1 DNA copy into the genome, causing mutations and damage. Reverse transcription also happens in the cytoplasm, where it creates RNA:DNA hybrids, aberrant products that can trigger inflammation. The detailed structure of the L1 RT enables development of specific inhibitors that may become effective therapies for autoimmune diseases, cancer, neurodegeneration and other diseases.

## Figures and Tables

**Extended Data Table 1:**
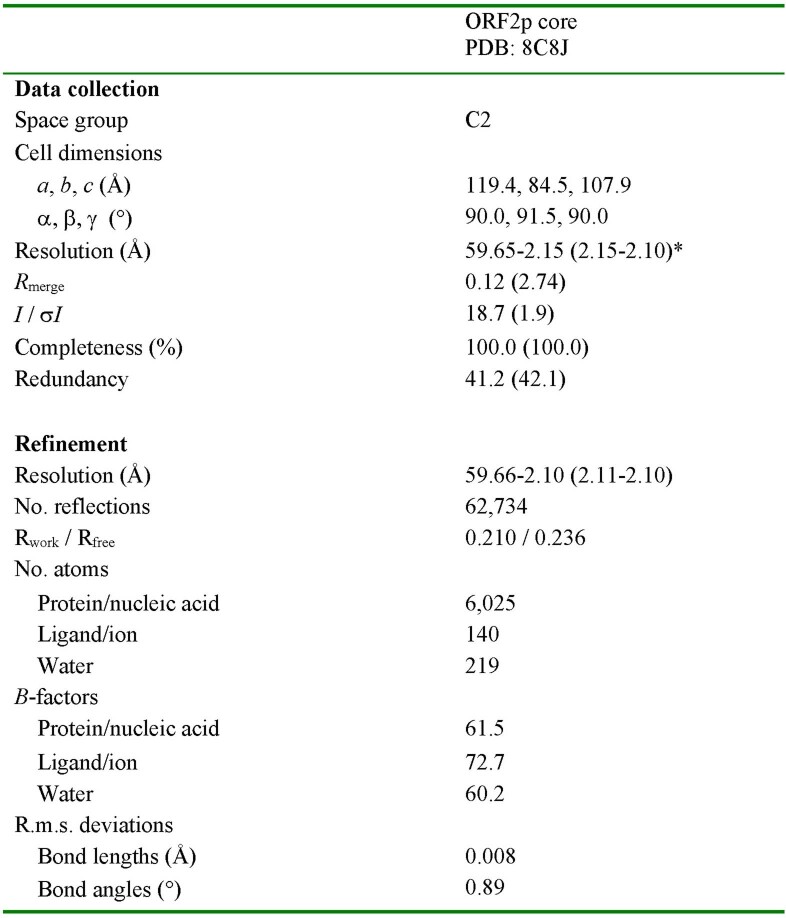
Data collection and refinement statistics (molecular replacement)

**Extended Data Table 2:**
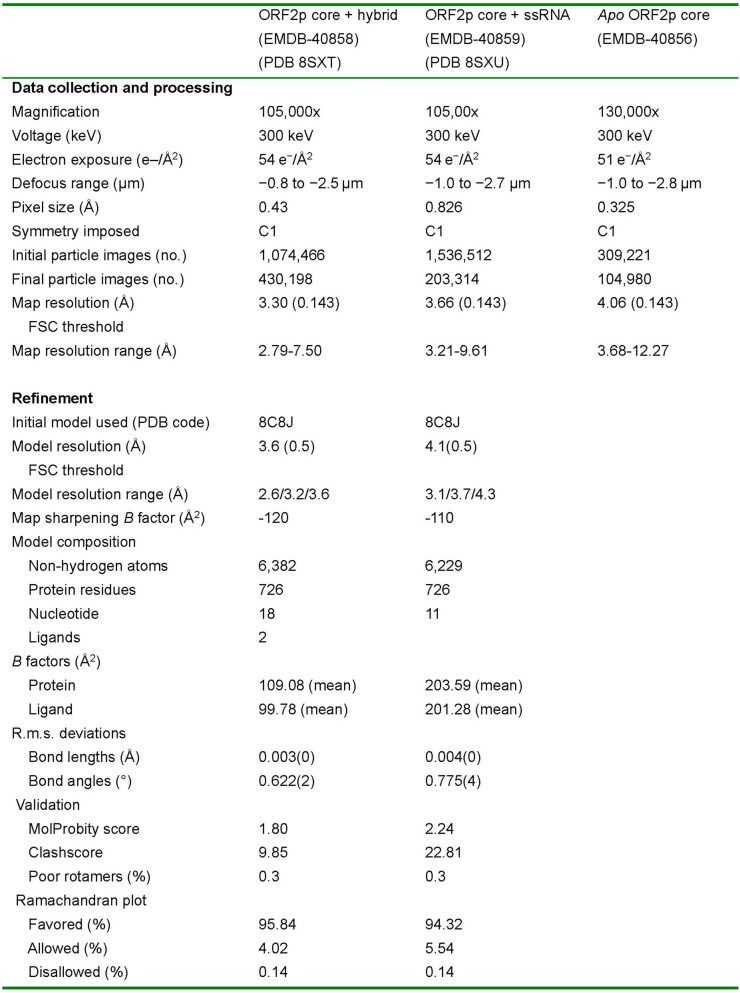
Cryo-EM Data collection, refinement, and validation statistics

## Data Availability

The coordinates for the ORF2p crystal structure have been deposited in the Protein Data Bank (PDB ID: 8C8J). Single-particle cryo-EM maps for the ORF2p core have been deposited in the Electron Microscopy Data Bank and their associated model coordinates in the Protein Data Bank under accession codes EMD-40858, PDB ID:8SXT (heteroduplex); EMD-40859, PDB ID:8SXU (oligo(A)); EMD-40856 (*apo*). Raw videos and motion-corrected micrographs for *apo* ORF2p have been deposited in the Electron Microscopy Public Image Archive under accession code EMPIAR-11556. The mass spectrometry proteomics data have been deposited at the ProteomeXchange Consortium (http://proteomecentral.proteomexchange.org) through the PRIDE partner repository with dataset identifier PXD038615. Files containing the input data, scripts and results of integrative modelling are available at https://github.com/integrativemodeling/ORF2p and the nascent integrative modelling section of the worldwide Protein Data Bank (wwPDB) PDB-Dev repository for integrative structures and corresponding data under accession code PDBDEV_00000211. AlphaFold2 predictions, molecular dynamics simulation results and full-atom versions of best-matching models are available from the ModelArchive repository (https://www.modelarchive.org/doi/10.5452/ma-fejd6, https://www.modelarchive.org/doi/10.5452/ma-joo4d, https://www.modelarchive.org/doi/10.5452/ma-lzyrq,https://www.modelarchive.org/doi/10.5452/ma-xlzzy, https://www.modelarchive.org/doi/10.5452/ma-9wovj). New plasmids have been deposited at Addgene.

## Code availability

Software for the evolutionary analysis is available at https://github.com/dfhoyosg/Plexy.
