# Supplementary material for: Structures, functions and adaptations of the human LINE-1 ORF2 protein
Source: Nature. 2023 Dec 14;626(7997):194–206. doi: 10.1038/s41586-023-06947-z (PMC10830420; doi:10.1038/s41586-023-06947-z)
Supplement: Supplementary file 1 — Supplementary background and discussion: further L1 background, relation to disease and mechanistic information; Methods; Figs. 1–13: overview of cryo-EM and further biochemical, modelling, and structural information and comparisons; Tables 1–5: summary of evolutionary and modelling datasets, plasmids and affinity reagents; and references. [file 41586_2023_6947_MOESM1_ESM.pdf]

nature portfolio

https://doi.org/10.1038/s41586-023-06947-z

Supplementary information

Structures, functions and adaptations of the human LINE-1 ORF2 protein

In the format provided by the authors and unedited

Supplementary data for:
Structures, Functions, and Adaptations of the Human LINE-1 ORF2 Protein

Eric T. Baldwin, Trevor van Eeuwen, David Hoyos, Arthur Zalevsky, et al.
Lead contact: Martin S. Taylor, mstaylor@mgh.harvard.edu

Table of Contents

Supplementary Background and Discussion 1
Supplementary Methods 2
- Protein expression and purification 2
- Crystallization and structure determination of the ORF2p-8His core 2
- ORF2p reverse transcriptase activity assays 3
- Compounds 3
- Cell lines, plasmids, and affinity reagents. 3
- LINE-1 and RNA:DNA hybrid immunoflourescence 4
- Interferon reporter assay in THP1 cells 4
- LINE-1 dual luciferase retrotransposition assay 4
- Telomerase activity assay 4
- Western blotting 4
- Differential scanning fluorimetry 5
- Crosslinking mass spectrometry 5
- Cryo-EM sample preparation and data collection 6
- Single particle analysis of cryo-EM data 6
- Negative stain TEM of full-length Orf2p 7
- Integrative structure modeling of the ORF2p 7
- Modeling of ddTTP, d4T, and AZT bound to L1 RT 8
- Relative free energy of binding Calculations 8
- Evolutionary analysis 8
- 1. Regions of conservation based on the sequence and structure of ORF2p. 8
- 2. Evolutionary distance from other proteins. 8

Supplementary Figures 10
- Supplementary Figure 1. Summary of single particle cryo-EM data analysis. 10
- Supplementary Figure 2. Cryo-EM map analysis and validation. 12
- Supplementary Figure 3. Biochemical characterization of ORF2p core protein. 14
- Supplementary Figure 4. Sanger sequencing-like reaction confirms high molecular weight reverse transcriptase products are template jumps/switches. 16
- Supplementary Figure 5. ORF2p priming and extension on hairpin RNA substrates. 18
- Supplementary Figure 6. NRTI and NNRTI reverse transcriptase inhibitors. 19
- Supplementary Figure 7. TERT inhibition and comparative modeling of the ORF2p active site 20

Supplementary Figure 8. Activity and inhibition of ORF2p full-length vs. core and crosslinking mass spectrometry (XL-MS) of ORF2p 22
Supplementary Figure 9. Full-length ORF2p analyzed by EM and simulations 24
Supplementary Figure 10. Summary of integrative modeling, validation, and clustering of structural classes. 26
Supplementary Figure 12. Structural evolutionary analysis of ORF2p and its domains. 30
Supplementary Figure 13. Structural perplexity of ORF2p domains relative to the other proteins in the curated set. The 32
Supplementary Tables 34
Supplementary Table 1. Summary of identified crosslinks from crosslinking mass spectrometry 34
Supplementary Table 2. Summary of integrative modeling data. 35
Supplementary Table 3. Evolutionary analysis curated protein set 36
Supplementary Table 4. Plasmids used 37
Supplementary Table 5. Affinity reagents used 37
Supplementary References 38

Supplementary Background and Discussion

A large fraction of eukaryotic genomes consists of mobile elements: sequences that either encode protein machinery to mediate their propagation or co-opt other mobile element proteins to copy themselves. DNA 'cut and paste' transposons, like the maize elements discovered by Barbara McClintock[78], are no longer active in primates. Instead, recent primate evolution is dominated by RNA 'copy and paste' retrotransposons, in which RNA intermediates are integrated into the genome by encoded reverse transcriptase (RT) activity[79]. These are divided into two classes: (1) long-terminal repeat (LTR) retrotransposons, also called endogenous retroviruses (ERVs), similar to HIV-1 but no longer thought active in humans, and (2) active Long INterspersed Element-1 (LINE-1, L1) non-LTR retrotransposons[80-82]. Previously considered 'junk DNA', L1 is the only active protein-coding human transposon and is an important endogenous mutagen[82].

L1 encodes two proteins, open reading frame 1 protein (ORF1p), a homotrimeric chaperone likely involved in nuclear entry[83-87], and ORF2p, which has endonuclease (EN) and RT activities[88-90] and three additional domains with previously unknown functions. Both L1 proteins, but especially ORF2p, bind back to the L1 RNA that encodes them, a property termed '*cis* preference'[83,91-96]. Indeed, ORF2p is thought to bind to its encoding RNA co-translationally[91,96] and most ORF2p is thought to be bound to the L1 RNA poly(A) tail[83,97-99]. Cis preference is not perfect, however, and ORF2p will copy and insert any bound RNA, including cellular mRNA sequences and RNAs transcribed from Short INterspersed Element (SINE) sequences *Alu* and SVA (SINE/variable number tandem repeat (VNTR)/*Alu*). *Alu* SINEs have specific mechanisms of hijacking ORF2p at the ribosome[91]. Together, the molecular 'fossil' record of these sequences comprises about a third of the genome[81,100,101].

L1s are conserved to plants and thus L1s and their hosts have been co-evolving for 1-2 billion years[102] in an arms race: the transposon attempts to copy itself in a process called retrotransposition (**Fig. 1a**), while the host defends against this mutagenic process. Multi-layered host defenses recognize the L1 DNA and RNA sequences, proteins, and retrotransposition intermediates, notably including p53[103], which may have evolved to suppress mobile elements[83,92,103-108].

Numerous additional studies have contributed to knowledge that de-repressed L1 elements can contribute to human pathology through at least three distinct mechanisms: (1) DNA damage from insertions, abortive insertions, and aberrant L1 EN activity[104,106,109-112], (2) perturbation of cellular homeostasis in response to L1 activation[104,105,111], and (3) sterile inflammation ('viral mimicry') mediated by sensing of RT products (**Fig. 1b**)[104,113-116]. Key additional studies have implicated L1 in autoimmunity including systemic lupus erythematosus (SLE), Sjögren's syndrome (SS), and psoriasis, neurodegeneration, and age-related macular degenartion[117-120]. In cancer, additional studies contributed to the concepts of viral mimicry and the p53-L1 relationship[121-126]. Accordingly, RT inhibitors have shown promising results in numerous model systems[113,114,117,118,127,128], and a number of studies have shown inhibition of L1 retrotransposition by NRTIs in cells[129-133]. In contrast with HIV-1 RT, where high resolution structural understanding has led to evidence-based therapy[134-137], limited understanding of L1 ORF2p structure and function has restricted rational inhibitor development and dissection of the underlying pathophysiology.

Biochemically, non-templated addition is also seen in retroviral RTs[138], and the 5' RNA cap may facilitate this activity as well as base pairing to facilitate template switching or jumping[139]. In R2 these activities are partially understood mechanistically and structurally[140-142]. These activities are likely involved in the transition from first to second strand synthesis (**Discussion**). The equivalent tower lock region in R2 as that in ORF2p was previously shown to contact RNA[143], although R2 does not have a tower and the baseplate does not have a PCNA-binding PIP box. PCNA recruits RNase H2 for efficient L1 retrotransposition[144]; RNase H2 is mutated in the Mendelian interferonopathy Aicardi Goutières Syndrome[144], and these patients respond clinically to RT inhibitors[145].

Supplementary Methods

Divergence times between species were obtained from TimeTree 5[102]. Web servers Dali[146] and Foldseek[147] were used for similarity searches.

Protein expression and purification

ORF2p core (residues 238-1061, tower-fingers-palm-thumb-wrist) was expressed in *E. coli* as an N-terminal His6-MBP fusion with a 3C protease cleavage site (pAMS823) as previously reported[132] with modification. Cells were lysed in a microfluidizer (Microfluidics) in 500 mM NaCl, 10% glycerol, 1 mM TCEP, 25 mM Imidazole, and 50 mM HEPES pH 8.0, purified by Ni-NTA and heparin affinity, tag cleaved using 3C protease, protease removed using heparin affinity, and polished using size exclusion on a Superdex 200 column (Cytiva) in SEC buffer (500 mM NaCl, 5% glycerol, 2 mM $MgCl_2$, 0.5 mM TCEP, and 20 mM HEPES pH 8.0) with monodisperse fractions corresponding to the theoretical mobility of a monomer at 97 kDa. Mutant and subsequent WT ORF2p core proteins were purified similarly but with C-terminal His8 and lacking the N-terminal MBP. For crystallography, ORF2p-His8 core was purified as above but the final size exclusion polishing step used low-salt SEC buffer (150 mM NaCl instead of 500 mM) and the pooled fractions were concentrated to 5-6mg/ml, aliquoted and flash frozen in liquid nitrogen. Full-length ORF2p (1-1275) using a codon-optimized ORFeus-Hs sequence[148] and a C-terminal 3C-3xFlag tag[92] was cloned into a customized insect vector pDARMO-PolH2.1 (pMT692)[149], expressed in SF9 insect cells using the MultiBac EMBacY system[150](Geneva Biotech), purified by Flag and Heparin affinity, and polished on size exclusion on a Superdex 200 column (Cytiva) in SEC buffer, with monodisperse fractions corresponding to the theoretical mobility of a monomer at ~150 kDa used for further structural experiments. For single nucleotide gel-based assays, HIV and HERV-K RTs were expressed and purified from SF9 insect cells using the MultiBac system, as previously reported[151]; full-length ORF2p with C-terminal His8 tag was expressed and purified analogously, as a fusion polyprotein containing N-terminal HERV-K and TEV proteases followed by TEV cleavage site (ENLYFQG) to facilitate post-translational processing, which results in a single glycine residue at the N terminus.

Crystallization and structure determination of the ORF2p-8His core

Chain-terminated hybrid duplex was prepared by incubating RNA-template and DNA-primer oligos at 95°C for 3 mins and cooling to 4°C over 1 hour (oligos supplied by IDT: DNA-5'GCGCTTTC[ddC]-3' / RNA-5'-UUAGGAAAGCGC-3'). Aliquots of ORF2p-His8 core were thawed, allowed to equilibrate to room temperature, diluted to 3 mg/mL with 50 mM NaCl, mixed with 2 mM $MgCl_2$, 2 mM dTTP and a 1.3:1 molar ratio of hybrid duplex. The resulting complex was incubated at room temperature for 30 minutes and used to set up a range of commercial sparse matrix crystallization screens. The initial hit was obtained in Proplex screen (Molecular Dimensions), condition D7 (0.1 M sodium citrate pH 5.5 and 15% PEG6000). These crystals were small, soft, difficult to handle and only diffracted to ~3.7 Å resolution, and data were also highly anisotropic. Sequential grid-screen optimizations were conducted to optimize pH, PEG molecular weight, PEG concentration and protein:well solution mixing ratio. Different combinations of organic solvents and salts were also extensively screened both as crystallization additives and in combination with additional PEG as post-growth order enhancement systems. The final crystals used to generate the data presented here were grown from 18% PEG8000, 0.1 M sodium citrate pH 5.6, 0.2 M NaCl, 10% DMSO and 5% 1,4-dioxane. For data collection, crystallization drops were layered with stabilizing solution (27.5% PEG8000, 20% DMSO, 0.05 M sodium citrate pH5.6) and incubated for 1 hour prior to harvesting by immersion in liquid nitrogen. Optimized crystals diffracted to ~2.1 Å but still exhibited up to 1.0 Å difference in resolution between the best and worst reciprocal lattice directions. Merging multiple datasets was found to greatly reduce this axial resolution gap. Final data, derived from merging six crystals, have <0.4 Å variation between best and worst resolution limits. All data were collected at Diamond Synchrotron, Beamline I03 (λ=0.976 Å), using a Dectris Eiger2 XE 16M detector. Datasets were indexed and integrated with DIALS, scaled and merged with Aimless and phased by molecular replacement with Phaser using AlphaFold model[152] AF-000370-F1 truncated to residues 238-1061 and with the tower domain removed from the search model. The structural model was rebuilt using Coot[153] and refined with Buster[154]. The final structure has Ramachandran angles favored/allowed/outlier (%) of 96.39/3.61/0.00 and further refinement statistics are found in **Extended Data Table 1**. Contact analysis between ORF2p and ligands was performed by the PLIP

server[155] and manually checked with cutoff of 2.5-3.3 Å for polar interactions and 3.7 Å for van der Waals interactions; dTTP identified contacts contain both incoming nucleotide and bound magnesium[156].

ORF2p reverse transcriptase activity assays

Microwell assays were performed using the reverse transcriptase assay, colorimetric (Roche) according to the manufacturer's instructions, with the supplied poly(A) template and oligo(dT)$_{15}$ primer. ORF2p fractions were diluted for assay in lysis/binding buffer (50 mM Tris, 80 mM potassium chloride, 2.5 mM DTT, 0.75 mM EDTA, and 0.5% Triton X-100; pH 7.8) and incorporation of digoxigenin- and biotin-labeled dUTP into DNA was measured by absorbance at 405 nm as compared to a 490 nm reference. Gel-based RT activity assays consisted of pre-incubating RTs with annealed DNA/RNA, DNA/DNA, or RNA/RNA 5'-end-radiolabeled or 5'-end-Cy5- or FAM-labeled template:primer duplex or hybrid duplex and, where indicated, inhibitor in the presence of 0.1-1 µM dNTP or NTP mixture, 0.25 mM EDTA, 50 mM NaCl, and 25 mM Tris (pH 8) for 10 min at 37°C. Labeled nucleic acids were purchased from Dharmacon or IDT. Unless otherwise indicated, 15 µL reactions were initiated by the addition of 1.3 mM $MgCl_2$, incubated for 10 min at 37 °C, and then stopped by the addition of 15 µL of formamide/EDTA (25 mM) mixture and incubated at 95 °C for 10 min. 3 µL reaction samples were subjected to denaturing 8 M urea 20% PAGE to resolve products followed by signal quantification (ImageQuant 5.2, GE Healthcare Bio-Sciences) through phosphorimaging (Amersham Typhoon 5, Cytivia). Scanned gel images are cropped and corrected for distortion artifacts with contrast uniformly increased to facilitate the visualization of minor products; original images are provided in an Extended Data file.

For HTRF RT assays[157], 25 nM ORF2p core and 12.5 nM template:primer was incubated at 25°C for 60 minutes with 10 nM of fluorescein-12-dUTP (Thermo), 1 µM each (dATP,dCTP,dGTP), and test compound in a 15 µL reaction with buffer containing 50 mM Tris-HCl, 50 mM KCl, 10 mM MgCl2, 10 mM DTT, pH 8.1, and 1% final DMSO in 384-well format in duplicate. 5 µL detection reagent was added (streptavidin-terbium cryptate, 20 mM EDTA in PPI buffer, Cisbio Bioassay), and the mixture was incubated at 25 °C for 30 minutes. Fluorescence was then read at ex/em=337/485 nm and ex/em=337/520 nm on an Envision 2104 plate reader (Perkin Elmer). The fluorescence ratio at 520/485 nm was used to calculate inhibition, with the DMSO sample as 0% inhibition and no enzyme as 100% inhibition. IC_{50} was calculated with a 4-parameter non-linear regression equation. Template:primer mixtures were pre-annealed for 60 min at room temperature and consisted of poly(rA$_{45}$) and biotin-oligo(dT)$_{16}$ (Generay Biotech) for NNRTIs. For NRTIs, the following template:primer pair was instead used:

```
3'        UAAGACUGAUUUUCCCAGACUCCCUAGAGAUCAAUG
5' Biotin-TTCTGACTAAAAGGGTCTGAGGGAT
```

and the nucleotides used in the assay were: for d4T-TP and AZT-TP, 1 µM each (dATP,dCTP,dGTP) and 10 nM fluorescein-12-dUTP (PerkinElmer); for carbovir-TP, 1 µM each (dATP,dCTP,dTTP) and 10 nM fluorescein-12-dGTP (Perkin Elmer); for 3TC-TP and ddCTP, 1 µM each (dATP,dGTP,dTTP) and 10 nM fluorescein-12-dCTP (Perkin Elmer). All RT assays were performed under conditions of initial velocity.

Compounds

NRTIs lamivudine (3TC), stavudine (d4T), emtricitabine (FTC), zidovudine (ZDV or AZT), tenofovir (TFD) were purchased from SelleckChem; GBS-149[131] was custom synthesized at Pharmaron; POC d4T prodrug (d4T bis(isopropoxycarbonyloxymethyl)phosphate)[158] was custom synthesized at Pharmaron. NRTI triphosphates were obtained from the following sources: carbovir and entecavir triphosphates were custom synthesized at NuBlocks. Stavudine (d4T) and zidovudine (AZT) triphosphates and tenofovir diphosphate were purchased from Jena Bioscience; emtricitabine (FTC) triphosphate and lamivudine (3TC) triphosphate were purchased from Carbosynth; ddT and ddC triphosphates were purchased from Sigma Aldrich. For NNRTIs: foscarnet was purchased from Houzhuang Shan (EB2016263-030F1); nevirapine, rilpivirine, etravirine, delavirdine, and efavirenz were purchased from MedChemExpress. cGAS inhibitor G140[159] was purchased from InvivoGen.

Cell lines, plasmids, and affinity reagents.

HeLa and U2-OS cells were cultured in DMEM with 10% heat inactivated fetal bovine serum (IFS) and 4.5 g/L glucose containing 2 mM GlutaMAX (Thermo), 100 IU/mL penicillin, and 100 µg/mL streptomycin. THP1 cells were cultured in RPMI 1640, 10% heat-inactivated fetal bovine serum, 25 mM HEPES, 10 µg/mL blasticidin, and 100 µg/mL Zeocin. HeLa Tet-On 3G cell line was from Takara; MCF7, HeLa and U2-OS from American Type

Culture Collection (ATCC); THP1-Dual and THP1-Dual KO-TREX1 cells were from InvivoGen. All cell lines were maintained at 37 °C and 5% CO2 and validated and tested for mycoplasma. All plasmids and affinity reagents are described in **Supplementary Tables 4-5**, respectively and available from Addgene at https://www.addgene.org/browse/article/28243724/.

LINE-1 and RNA:DNA hybrid immunofluorescence

Catalytically inactive D210N human RNase H1 (dRNH1)[160] was expressed as a GFP fusion in E. Coli BL21(DE3), induced using 200 µM IPTG overnight at 16°C, purified by sequential Ni-NTA affinity, heparin affinity, and gel filtration, and the monodisperse fraction was concentrated to 7 mg/ml. 150,000 HeLa or U2OS cells were plated on 22 mm glass coverslips in 6-well dishes and transfected with 2 µg of plasmid DNA using Lipofectamine 3000 (Thermo) according to the manufacturer's instructions, with or without 50 µM d4T. 24 hours later, cells were fixed in ice cold methanol and incubated at -20°C for 10 minutes, washed twice with PBS containing 10 mM glycine and 0.2% sodium azide (PBS/gly). Staining with primary and secondary antibodies was done for 20 min at room temperature by inverting coverslips onto Parafilm containing 45 µL drops of PBS/gly supplemented with 1% BSA and appropriate antibodies or dRNH1 reagent. Affinity reagents used were GFP-dRNH1 (0.1 µg/mL), rabbit monoclonal S9.6 (1:1000), mouse anti-Flag M2 (1:500), mouse anti-ORF1 4H1[92] (1:4000), GFP-tag polyclonal (1:2000), Alexa Fluor 488 conjugated anti-rabbit IgG (1:1000), and Alexa Fluor 568 conjugated anti-mouse IgG (1:1000). For dRNA staining, coverslips were sequentially incubated with GFP-dRNH1, rabbit anti-GFP, and secondary anti-rabbit reagents. DNA was stained prior to imaging with Hoechst 33285 (0.1 mg/mL). Coverslips were mounted with Prolong Diamond (Thermo). Epifluorescent images were collected using a Leica DMi8 microscope and K8 camera using Leica Application Suite X (LAS X) software.

Interferon reporter assay in THP1 cells

The type I interferon response was evaluated using THP1-Dual and THP1-Dual KO-TREX1 cells (InvivoGen), which secrete a Lucia luciferase reporter gene under control of an interferon-responsive promoter. THP1-Dual KO-TREX1 cell were generated by stable biallelic knock-out of the TREX1 gene. Cell were treated with a dose titration of test compound in the presence of 1 µM 5-aza-2'-deoxycytidine (decitabine, Sigma, #189825), which de-represses LINE-1[121]. Type 1 Interferon and cell viability were assessed after five days of treatment. QUANTI-LUC solution containing stabilizer was added to the cell supernatant and luminescence was measured on a plate reader, and cells were assessed for cell viability using CellTiter-Glo (Promega, #G9683) according to the manufacturer's instructions.

LINE-1 dual luciferase retrotransposition assay

To assess the potency of inhibiting LINE-1 retrotransposon, a stable clonal dual luciferase L1 reporter cell line was generated and reported as described[130,133] in the HeLa Tet-On 3G cell line (Takara,). SB100x[161] was used to integrate pRT006.2, a vector similar to pYX056[130], which contains a bi-directional Tet-On promoter expressing both control Renilla luciferase and LINE-1 ORFeus-Hs Firefly luciferase antisense intron (AI) reporter[79,162]. A single cell clone was selected with the highest doxycycline-induced luciferase signal vs baseline. Cells were mixed with compounds and induced for reporter expression with 500 ng/mL doxycycline (Sigma, #D9891) for 72 hours. Luminescence was measured using the Dual-Glo Luciferase Assay System (Promega, #E2940) following the manufacturer's instructions, and the ratio of Firefly to Renilla Luciferase activity was used to measure retrotransposition.

Telomerase activity assay

The human telomerase assay was performed with telomerase in MCF-7 cell lysates using the Telo TAGGG Telomerase PCR ELISAplus kit (Roche). Test compounds (NTPs) were serially diluted in water, mixed with 0.2 µg of MCF-7 lysate, and pre-incubated at room temperature for 15 minutes. Then the reaction was carried out for 30 minutes, amplified using PCR, and visualized colorimetrically per the manufacturer's instructions.

Western blotting

Cells were lysed in ice cold RIPA buffer containing 1x protease inhibitor tablet (Thermo), centrifuged for 10 minutes, and clarified lysates were quantified by BCA assay. 25 µg of protein per lane was loaded, transferred to PVDF membranes (Cytivia), blocked in 5% (w/v) nonfat dry milk in TBST, incubated with primary antibody at the 5% BSA in TBST at 4°C overnight, and developed by chemiluminescence using HRP-conjugated secondary

antibodies (CST). ORF1p was blotted with clone 4H1 (Sigma MABC1152, 1:1,000); β-actin (CST 4970, 1:10,000).

Differential scanning fluorimetry

Lyophilized oligos for differential scanning fluorimetry (DSF) were reconstituted in RNase-free TE to 500 µM. To form a hybrid, an equimolar ratio of DNA primer (oligos supplied by IDT, 5’- GCGAAAAATTTCG[ddC]-3’) and RNA template (5’-GGAGCGAAAUUUUUCGC-3’) was mixed and diluted in DSF buffer (20mM HEPES-KOH pH 7.6, 100 mM sodium chloride, 1mM DTT, 2mM magnesium acetate) to a final concentration of 25 µM. Oligos were then annealed by heating them to 95°C and cooling them in a step gradient of 10°C every five minutes until 5°C in a thermocycler. Purified L1 ORF2p core protein was diluted in DSF buffer to a final concentration of 1 µM in the presence or absence of 5 µM RNA or DNA/RNA hybrid. Nineteen microliters per well of buffer only, protein or protein-nucleic acid mixture were transferred to a 384-well plate to which 1 µL of fivefold SYPRO Orange (Thermo Fisher S6650) was added. Fluorescence measurements were obtained using a TAQMAN 7900 QPCR (Life Technologies) machine monitoring the fluorescent signal at 570 nm over a temperature ramping from 20°C to 95°C. Melting temperatures (T_m) were calculated using DSF World[163] using sigmoid fitting and the normalized curves were plotted using Prism (GraphPad).

Crosslinking mass spectrometry

DNA-RNA hybrid was produced by resuspending the individual DNA and RNA oligos (sequences as in the cryo-EM duplex) in 500 mM NaCl to a final concentration of 500 µM. These solutions were mixed 1:1 (final concentration 250 µM) and annealed in a thermocycler as follows: 5 min at 95 °C, 45 min ramp to 25 °C and then 10 min ramp to 4 °C.

Purified full-length ORF2p and ORF2p core in SEC Buffer were crosslinked using BS3 (bis(sulfosuccinimidyl)suberate; ThermoFisher Scientific, #21580), with and without the addition of DNA:RNA hybrid, using a final protein concentration of 1 µg/µL in 500 mM NaCl (and 2.7 mM HEPES pH 8, 0.7% glycerol (v/v), 0.07 mM TCEP, 0.27 mM $MgCl_2$). To the samples containing DNA:RNA, the hybrid was at 1.5:1 molar ratio to ORF2p, with 2 mM dTTP. The mixtures were incubated for 1 hour on ice, prior to initiating crosslinking. BS3 solutions were prepared at different concentrations and added to the reaction mixtures accordingly, which were agitated in a thermal mixer at 750 RPM, 23 °C for 3 min. Crosslinking reactions were quenched by adding Tris to a final concentration of 100 mM from a stock solution of 500 mM NaCl, 500 mM Tris pH 8.0, and incubated at room temperature for 15 minutes.

For tryptic digestion and sample cleanup prior to LC-MS/MS analysis, the quenched crosslinking reactions were first dried down using a centrifugal vacuum concentrator. The dried reaction products were resuspended in 25 µL of S-trap ‘high recovery’ solution (5% SDS, 8 M urea, 100 mM glycine pH 7.55), reduced (TCEP 5 mM, 55ºC, 15 minutes), alkylated (20 mM MMTS at room temperature for 10 minutes) and Lys-C/trypsin (Promega, #V5071) digested on S-trap micro columns (Protifi) following the manufacturer’s instructions. Eluted, digested peptides were dried using a centrifugal vacuum concentrator and resuspended in 25 µL of 0.1% (v/v) formic acid in water (MS grade, ThermoFisher Scientific).

Mass spectrometry of the digested reaction products was conducted on a Thermo Scientific Orbitrap Exploris 480. The mobile phase consisted of 0.1% (v/v) formic acid in water (A) and 0.1% (v/v) formic acid in acetonitrile (B). Samples were loaded using a Dionex Ultimate 3000 HPLC system onto a 75 µm x 50 cm Acclaim PepMapTM RSLC nanoViper column filled with 2 µm C18 particles (ThermoFisher Scientific, #164540) using a 60 min LC-MS method at a flow rate of 0.3 µL/min as follows: 3% B over 3 min; 3 to 50% B over 45 min; 50 to 80% B over 2 min; then wash at 80% B over 5 min, 80 to 3% B over 2 min and then the column was equilibrated with 3% B for 3 minutes (MS data were acquired over the entire program, including the wash). For precursor peptides and fragmentation detection on the mass spectrometer, MS1 survey scans (m/z 375 to 1500) were performed at a resolution of 120,000 with a 300% normalized AGC target. Peptide precursors from charge states 2-6 were sampled for MS2 using DDA. For MS2 scan properties, HCD was used, and the fragments were analyzed in the Orbitrap with a collisional energy of 30%, resolution of 15,000, standard AGC target, and a maximum injection time of 50 ms.

RAW data was searched using pLink 2.3.9[164], MaxLynx (MaxQuant 2.1.4.0)[165], and Proteome Discoverer 2.4 with the XlinkX plugin[166]. Among the search parameters, a maximum of three missed cleavages were allowed, and a static modification on cysteines corresponding to thiomethylation by MMTS. The max false discovery rate was set to 1%. Crosslinks found in automated searches were manually validated by inspecting MS2 spectra signal-to-noise and percentage of b and y fragments detected (**Supplementary Table 1**). Concentrations of BS3 crosslinker were 10 and 30 μM for ORF2p core and 30 and 100 μM for full-length ORF2p. A raw list of crosslinks, initially identified with pLink, was filtered with the following conditions: (i) crosslink had to be identified by at least one other engine (Proteome Discoverer or MaxLynx), (ii) crosslinked residues had to be observed directly, or fragments must cover more than 50% of the crosslinked peptide. Duplicate residue pairs (which sometimes corresponded to different peptides) were removed and filtered crosslinks were then divided into 3 lists: (1) present only in the core, (2) present only in full-length, (3) present in both.

Cryo-EM sample preparation and data collection

Samples for cryo-TEM studies were prepared by mixing purified ORF2p with 1.5x molar excess of annealed heteroduplex (oligos supplied by IDT: DNA-5'GCGAAAATTTCG[ddC]-3' / RNA-5'-GGAGCGAAAUUUUCGC-3') or single stranded poly$(A)_{25}$ and diluted to a final concentration of 0.15 mg/mL with EM buffer (20 mM HEPES pH 7.6, 150 mM sodium chloride, 2 mM magnesium acetate, 2 mM DTT) and 2.5 mM dTTP. ORF2p core and mixed nucleic acids were incubated on ice for 15 minutes to allow for equilibration prior to preparation of grids. A combination of R1.2/1.3 Quanitfoil 300 mesh and R0.6/1 200 mesh holey carbon grids were glow discharged for 60 seconds using an a Pelco easiGlow glow discharger. Vitrified grids were prepared by applying 2μL of ORF2p core with or without bound nucleic acid to grids, blotting manually for 2 seconds (200 mesh) or 3 seconds (300 mesh) from behind grids with Whatman 41 grade filter paper and plunging into liquid ethane using LeicaEM CPC manual plunger. Grids were prepared in batches and screened with Talos Artica at the Rockefeller University Evelyn Gruss Lipper Cryo-electron Microscopy Resource Center.

An initial dataset of 9442 micrographs of ORF2p core-template:primer was collected using a spherical aberration corrected 300 kV Titan Krios (Thermo Fisher Scientific) equipped with a GIF BioQuantum and K3 camera (Gatan). Micrographs were taken using with SerialEM[167] at a nominal magnification of 105,000x in super-resolution mode at a nominal pixel size of 0.43 Å/pixel over a defocus range of −0.8 to −2.5 μm with a step size of 0.1 μm and using a 20 eV energy filter slit. Movies were recorded with a dose per frame of 1.08 $e^{-}/Å^2$ in dose-fractionation mode with 50 subframes over a 2 second exposure to give a total electron flux of approximately 54 $e^{-}/Å^2$. After processing these data (described in detail below) a slightly anisotropic reconstruction was obtained, with cryoEF[168] detecting a minor gap in Fourier space and calculating a tilt angle of 30 degrees to fill in. A second dataset of 1828 micrographs using the same data collection parameters and 30-degree tilt was collected and combined with untilted data. A similar approach was taken for single stranded oligo$(A)_{25}$ sample, where an initial untitled dataset of 5815 micrographs and then a 30-degree tilted dataset of 6809 micrographs were collected. ORF2p core-oligo$(A)_{25}$ data were collected using a 300 kV Titan Krios (Thermo Fisher Scientific) equipped with a GIF BioQuantum and K3 camera (Gatan). Micrographs were taken with Leginon[169] in counted mode at a nominal pixel size of 0.826 Å/pixel over a defocus range of −1.0 to −2.75 μm with a step size of 0.25 μm and using a 20 eV energy filter slit. 200 mesh grids were primarily used for tilted data collection due to larger mesh areas. Movies were recorded with a dose per frame of 1.16 $e^{-}/Å^2$ in dose-fractionation mode with 48 subframes over a 2.2 second exposure to give a total electron flux of approximately 54 e^{-}/Å2. A single untitled dataset for *apo* ORF2p core was collected using a 300 kV Titan Krios (Thermo Fisher Scientific) equipped with a GIF BioQuantum and K3 camera (Gatan). Micrographs were taken using with SerialEM[167] at a nominal magnification of 130,000x in super-resolution mode at a nominal pixel size of 0.325 Å/pixel over a defocus range of −1.0 to −2.8 μm with a step size of 0.2 μm and using a 20 eV energy filter slit. Movies were recorded with a dose per frame of 1.32 $e^{-}/Å^2$ in dose-fractionation mode with 38 subframes over a 2 second exposure to give a total electron flux of approximately 51 $e^{-}/Å^2$.

Single particle analysis of cryo-EM data

The untilted ORF2p core-template:primer processed independently initially as follows. Dose-fractionated movies were gain-normalized, motion-corrected and dose-weighted using MotionCor2[170] and then imported into cryoSPARC v.3.1.0[171] for downstream processing starting with contrast transfer function (CTF) correction with patch CTF estimation. A particles from subset of 2000 micrographs were picked using cryoSPARC blob picker

and subjected to reference free 2D classification. The consistent classes from 2D classification were used as templates for template-based picking on all micrographs, with picked particles subjected to reference free 2D classification. Particles from self-consistent classes were selected and subjected to *ab initio* model generation and then three rounds of heterogenous refinement. The highest quality reconstruction, comprising 255,612 particles, was subset and refined using non-homogenous refinement[172], resulting in a reconstruction at 3.49 Å resolution. Fourier coverage appeared incomplete and cryoEF[168] was used to determine an optimal tilt angle for additional data collection.

The final datasets for the three samples were processed in a similar fashion. Movies were motion- and CTF-corrected as described above. 2D classes from the untilted ORF2p core-template:primer were used to template pick each dataset and particles were subjected to 2D classification and *ab initio* model generation independently. Particles from tilted and untilted datasets were combined at this point for heterogenous refinement. The particles from the highest quality reconstruction in each combined dataset was transferred to Relion v3.1[173] using pyem[174]. Combined particle sets were extracted in in Relion from micrographs that were CTF corrected with CTFFIND 4.1[175] and subjected to 3D classification with or without alignment. Selected classes were then processed using iterative rounds of 3D auto-refinement, Bayesian polishing and CTF refinement. Particle orientations and CTF parameters were imported back into cryoSPARC and a final refinement was generated using non-uniform refinement. Maps for ORF2p core-template:primer and -poly$(A)_{25}$ were postprocessed with both global B factor sharpening and locally sharpened with deepEMhancer[176] with both postprocessed maps and unfiltered half-maps deposited in EMDB. *Apo* ORF2p core was low pass filtered using the Volume Utility in cryoSPARC. Data processing steps and map validation are presented in detail in **Supplementary Figs. 1-2.** The ORF2p crystal structure was from this study was used as the starting model for model building and refinement using Coot[153] and Phenix[177], respectively. Structural models were generated for ORF2p core bound to RNA:DNA hybrid and ssRNA and summary statistics for maps and models are found in **Extended Data Table 2**.

Negative stain TEM of full-length Orf2p

Full-length ORF2p for negative stain TEM was prepared by adding 1.5x molar excess of RNA template:DNA primer hybrid or L376 RNA to full-length ORF2p at a final protein concentration of 0.10 mg/mL. After equilibration, 2 µL full-length ORF2p was applied to glow-discharged carbon-coated copper grids and stained with 1% uranyl acetate. Grids were imaged with a FEI Tecnai GA Spirit BioTwin TEM with AMT BioSprint 29 camera. Particles were picked and 2D classes generated using the sphire software suite[178]. Class averages were postprocessed in EMAN2[179] prior to being passed to IMP. L376 RNA was produced by run-off transcription using T7 RNA polymerase from pBS27 digested with BsaI, which produces a 376 nt RNA corresponding to the last 362 residues of L1RP (His1224 through the end of the 3' UTR) with a 14 A tail.

Integrative structure modeling of the ORF2p

Integrative structure determination proceeded through the standard four stages[180-182]: (1) gathering data, (2) representing subunits and translating data into spatial restraints, (3) configurational sampling to produce an ensemble of structures that satisfies the restraints, and (4) analyzing and validating the ensemble structures and data. The data should be understood in a broad sense and can include results of other modeling experiments following the same four-step approach, forming a hierarchical structure. The integrative structure modeling protocol (i.e., stages 2, 3, and 4) (**Supplementary Table 2)** was scripted using the Python Modeling Interface (PMI) package, a library for modeling macromolecular complexes based on our open-source Integrative Modeling Platform (IMP) package[183] and executed in IMP 2.18.

For some analyses and visualization, we computed an atomic model from a coarse-grained integrative structure model by expanding the bead positions into the full-backbone structure[184], adding sidechains[185] and optimizing stereochemistry[186]. Structural analyses were performed with GROMACS[187] built-in tools and Python scripts using the MDanalysis v2.4.3[188] and ProDy v2.4[189] libraries. Particle radius was measured as the largest distance between the center of mass of an image and all non-zero pixels.

Modeling of ddTTP, d4T, and AZT bound to L1 RT

The L1 RT crystal structure in complex with dTTP was prepared with the Protein Preparation Workflow in Maestro (Schrödinger Suite version 2023-1) using default parameters to fill in missing side chains, optimize hydrogen bond assignments, and minimize the structure (convergence to 0.3 Å RMSD for heavy atoms). The structures for ddTTP, d4T and AZT were built by modifying the dTTP structure present in the L1 RT crystal structure. AZT bound to ORF2p was compared to the structure of HIV-1 RT bound to AZT[190]. The OPLS4 force field was customized for the ligands of interest using the Force Field Builder in Maestro with S-ANSI theory level (neutral structures) for geometry optimization. The newly built ligands were minimized in the context of L1 RT structure using the dTTP crystallographic binding mode as a starting pose. Appearances of clashes, which were only observed for AZT, were followed by minimization of the protein residues around the clash to attempt to relax the structure.

Relative free energy of binding Calculations

FEP+ (Schrödinger Suite version 2023-1) was used to construct a perturbation map including dTTP, ddTTP, d4T, and AZT in the context of the L1 RT crystal structure. The default perturbation protocol was used for the following pairs: dTTP/ddTTP, ddTTP/d4T, and d4T/dTTP; with 12 λ-windows and 10 ns of simulation per window. Perturbations including AZT (dTTP/AZT and ddTTP/AZT) used the Charge-hopping protocol with 24 λ-windows and 10 ns of simulation per window. The previously customized OPLS4 forcefield was used to carry out the FEP+ calculation of relative binding free energy and values were reported as $\Delta\Delta G$ changes with respect to ddTTP.

Evolutionary analysis

Our principal aim is to infer evolutionary similarity via protein structure, as has been done utilizing sequence. There is a fundamental issue with alignments, in that there is a trade-off between the coverage of an alignment and the quality of an alignment. We address this issue using information theory, building upon previous efforts[191] to derive distance metrics which can inform evolutionary similarity in groups of proteins.

1. Regions of conservation based on the sequence and structure of ORF2p.

We measured conservation against a curated set of 55 ORF2p sequences from vertebrates, including human ORF2p[192], to which we added LINE-1 sequences from 3 plants (corn, rice, and Arabidopsis thaliana, GenBank Y00086.1, AAG13524.1, and PIR: S65812, respectively). We computed a per-residue Shannon entropy of the aligned residues by both a multiple sequence alignment and a multiple structure alignment. The higher the entropy, the less conserved the residue. We conducted the multiple sequence alignment using Clustal Omega version 1.2.4[193] using default settings. We conducted the multiple structure alignment utilizing the MUSTANG algorithm version 3.2.4[194] using default settings. The Shannon entropy was computed for each aligned ORF2p residue index i in multiple sequence/structure alignment F as:

$$S_i = - \sum_{i \in F[r]} p_r \cdot \log_2 p_r \ .$$

For correlation to the scanning tri-alanine mutagenesis assay data[195], we utilized the mean value of the %WT retrotransposition efficiency across replicates.

2. Evolutionary distance from other proteins.

We manually curated a set of 50 experimental protein structures which contained RTs, RdRps, a DdRp, a dual DdRp/RdRp, and a number of "controls" which should have little resemblance to the other proteins. For RT and RT-like proteins, the polypeptide with polymerase activity is used; for other proteins, the entire biological assembly is used. The curated list is available in **Supplementary Table 3**. We utilized the MMLigner software version 1.0.2[191] to compute the alignments, enforcing the Maximum-Fragment Pair (MFP) library to have a maximum value of 5000 MFPs as we observed that for large structures, such as ORF2p (1275 amino acids in length). The default pruning was insufficient and additional pruning was required for significant alignments to be obtained. Additionally, we enforced that two proteins with no residue alignments should each contribute their null contributions.

The efficiency of an alignment can be determined via the compression for a given alignment, $\mathcal{C}(\mathcal{A})$:

$$C(\mathcal{A}) = I_{\text{null}} - I(\mathcal{A} \& \langle \mathcal{P}_1, \mathcal{P}_2 \rangle) \ .$$

Here, positive values indicate that the message length with the alignment, $I(\mathcal{A}\&\langle\mathcal{P}_1, \mathcal{P}_2\rangle)$, is shorter than the message length without the alignment , for two sets of protein coordinates ($\mathcal{P}_1$and $\mathcal{P}_2$). Negative values indicate the alignment is inefficient, which could result from an alignment with large coverage but poor quality. A value of zero indicates that there is no difference with respect to the message length without the alignment.

The null model is encoded in two parts -- a radial part and a directional part:

$$I_{\text{null}}(\vec{c}_i) = I_{\text{null}}^{\text{radius}}(r_i) + I_{\text{null}}^{\text{direction}}(\hat{x}_i) \ .$$

The radius is encoded with a normal distribution around 3.8 angstroms with a standard deviation of 0.4 angstroms.

$$I_{\text{null}}^{\text{radius}}(r_i) \sim -\log_2\left[\mathcal{N}(\mu = 3.8, \sigma = 0.4)\right] \ .$$

The direction is encoded with a 23-component Kent distribution which parameterizes the angular coordinates of an alpha carbon.

$$I_{\text{null}}^{\text{direction}} \sim -\log_2\left[\sum_{k=1}^{|M|=23} w_k f_k(\hat{x}; \vec{\theta}_k)\right] \ .$$

If there is a significant alignment, the information content required to encode two proteins is smaller as the entropy of the second protein's coordinates is smaller than that of the null distribution. The updated radial and angular probability distributions will depend on the alignment:

$$I_{\text{align}}(\vec{c}_i) = I_{\text{align}}^{\text{radius}}(r_i) + I_{\text{align}}^{\text{direction}}(\hat{x}_i) \ .$$

The radial component is transmitted over a χ^2distribution with three degrees of freedom (χ_3^2). This constrains the coordinates of protein 2 ($\mathcal{P}_2$) to the coordinates of protein 1 ($\mathcal{P}_2$). The directional component is transmitted using Bayesian updating of the components of the Kent distribution.

We can define an evolutionary distance based on the compression derived from an alignment, using an algorithm we term 'Plexy'. We compute the perplexity of the compressed information as follows:

$$\mathcal{D} = 2^{-\mathcal{C}(\mathcal{A})} \ .$$

Therefore, increased compression results in a smaller distance, and negative compression results in larger distances. This offers advantages over other metrics such as RMSD as this term inherently takes into account the quality and the length of an alignment.

We conducted pairwise structural alignments against all proteins within the curated list. We next desired to illustrate these pairwise distances on a two-dimensional plane. To do so, we computed the perplexity from the Normalized Compression Distance[196]. The Normalized Compression Distance is computed as:

$$\text{NCD} = \frac{I(\mathcal{A}\&\langle P_1, P_2\rangle) - \min[I_{\text{null}}(P_1),\ I_{\text{null}}(P_2)]}{\max[I_{\text{null}}(P_1),\ I_{\text{null}}(P_2)]} \ .$$

This is an approximation of Kolmogrov complexity. The "normalized perplexity" is then:

$$\text{D}_{\text{norm}} = 2^{\text{NCD}} \ .$$

We projected these distances using multi-dimensional scaling using the Python scikit-learn package[197].

Data and structural analysis and visualization

Data were plotted using combinations of Matplotlib v3.7.0, Seaborn, and pyCircos v0.3.0 packages and Prism (GraphPad). Structures were visualized with ChimeraX v1.5131[192].

Statistics and Reproducibility

All experiments were repeated at least two or three times with similar results. All gel-based experiments were repeated at least twice (Fig. 2d; Fig. 3a-e,g; Fig. 4 b,e; Extended Data Figs. 3-5, 7; Supplementary Fig. 3-5, 8). Microscopy experiments were repeated on four independent days and each condition was repeated in each experiment over at least two independent coverslips. The purification in Fig. 1c is representative of >15 experiments in four laboratories; the purification in Supplementary Fig. 9 is representative of 3 experiments. Negative stain experiments were performed at least twice with each bound nucleic acid species. For electrophoresis, original scans of cropped gels and blots are provided in a Source Data File.

Supplementary Figures

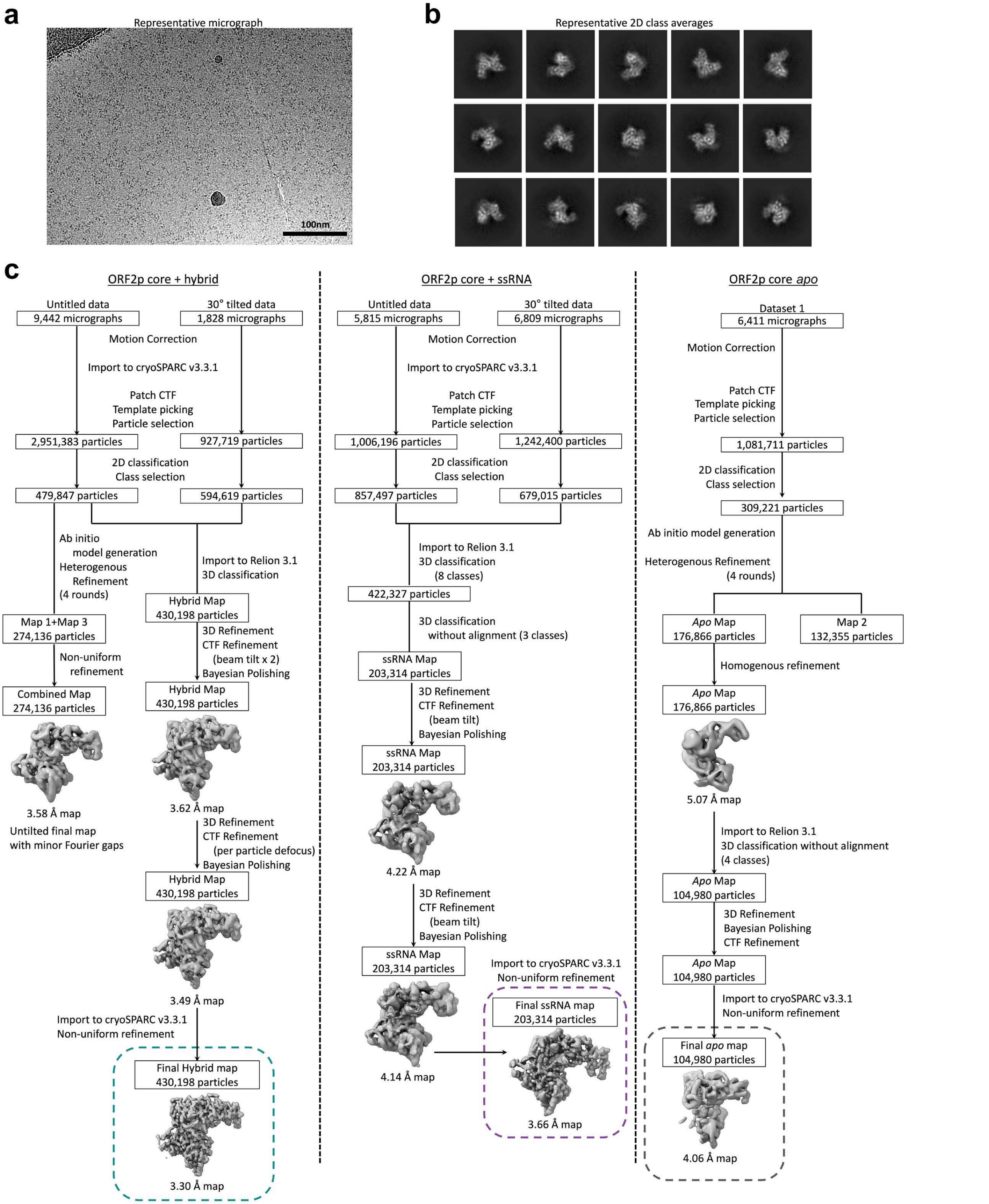

Supplementary Figure 1. Summary of single particle cryo-EM data analysis. **a**, Representative cryo-EM micrograph of ORF2p core with RNA template:DNA primer hybrid shows monodisperse and uniform particles. **b**,

cryoSPARC derived reference-free 2D classification of ORF2p core with clear secondary structure visible in class averages. **c**, Summary of single particle analysis for reconstructions of ORF2p core in different nucleotide ligand states. From an initial untilted data set of ORF2p core bound to the template:primer hybrid, a 3.58 Å resolution reconstruction was obtained with clear density for the bound hybrid though a Fourier gap was identified. To fill in Fourier gaps, additional datasets were collected at 30° tilt for ORF2p core bound to template:primer hybrid and ssRNA. Cryo-EM data were processed by motion correcting movies in MotionCorr2 followed by import into cryoSPARC where micrographs were CTF corrected. An initial set of 2D class averages from a subset of the data were used for template-based particle picking. Picked particles were sorted by 2D classification and the tilted and untilted datasets were combined in Relion 3.1 for 3D classification. The most complete 3D classes were selected and refined with iterative rounds of 3D auto refinement, CTF refinement and Bayesian polishing. Final maps were obtained by importing particles and refined CTF values into cryoSPARC for non-uniform refinement. Tilted data for *apo* ORF2p was not necessary because a larger range of views were obtained from untilted data.

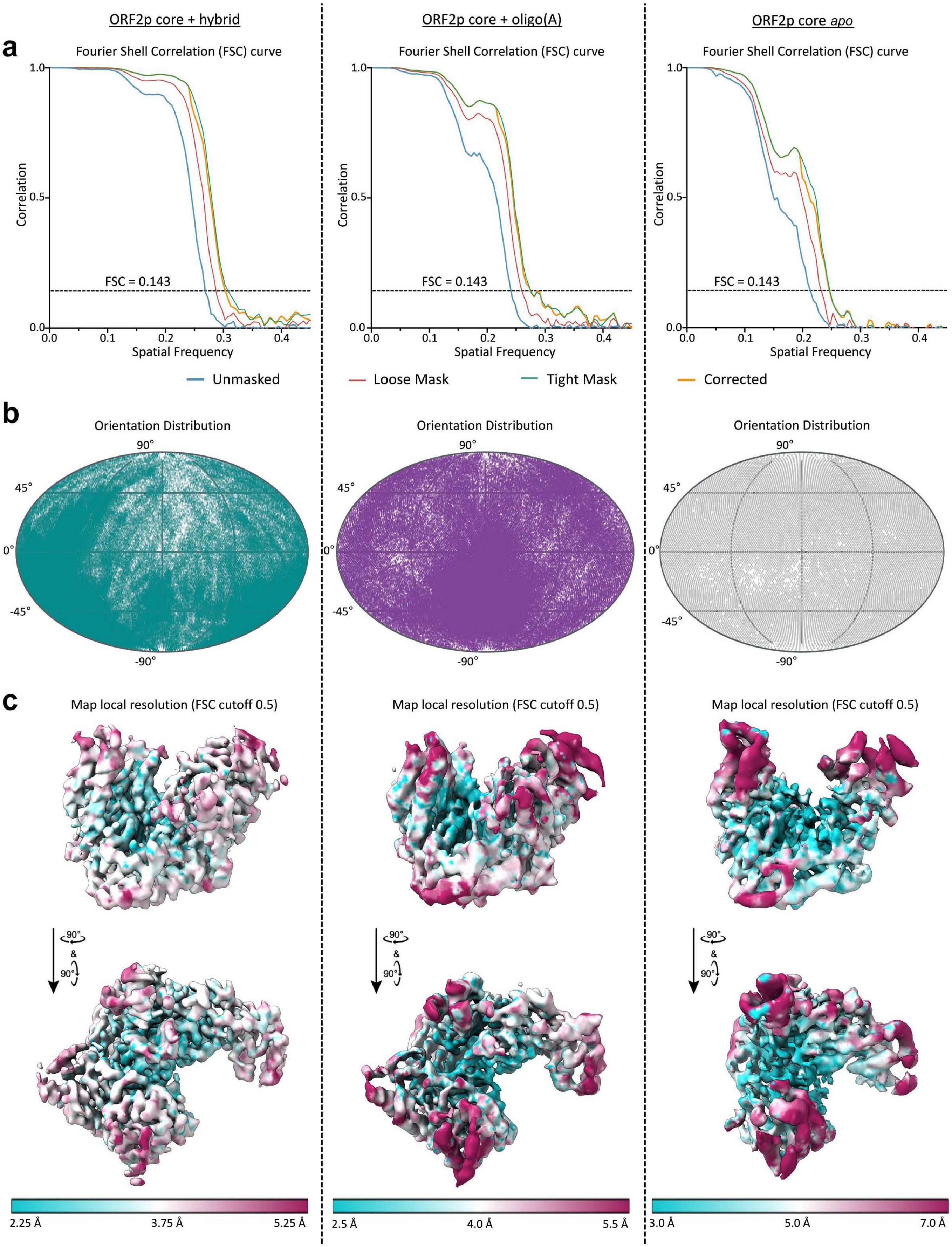

Supplementary Figure 2. Cryo-EM map analysis and validation. **a**, Fourier shell correlation (FSC) curves show resolutions of 3.30 Å (hybrid, left), 3.66 Å (ssRNA, middle) and 4.02 Å (*apo,* right*)* for final reconstructions

of ORF2p bound to respective substrates at FSC threshold of 0.143 (dotted line). **b**, Orientation distribution plots for ORF2p core cryo-EM reconstructions show complete orientation coverage. **c**, Single particle reconstructions of ORF2p core colored by local resolution as calculated by MonoRes. For all maps, the palm and flanking fingers and thumb are the highest resolution portions of the reconstruction with more distal elements (wrist or tower) being more flexible relative to palm and more poorly resolved.

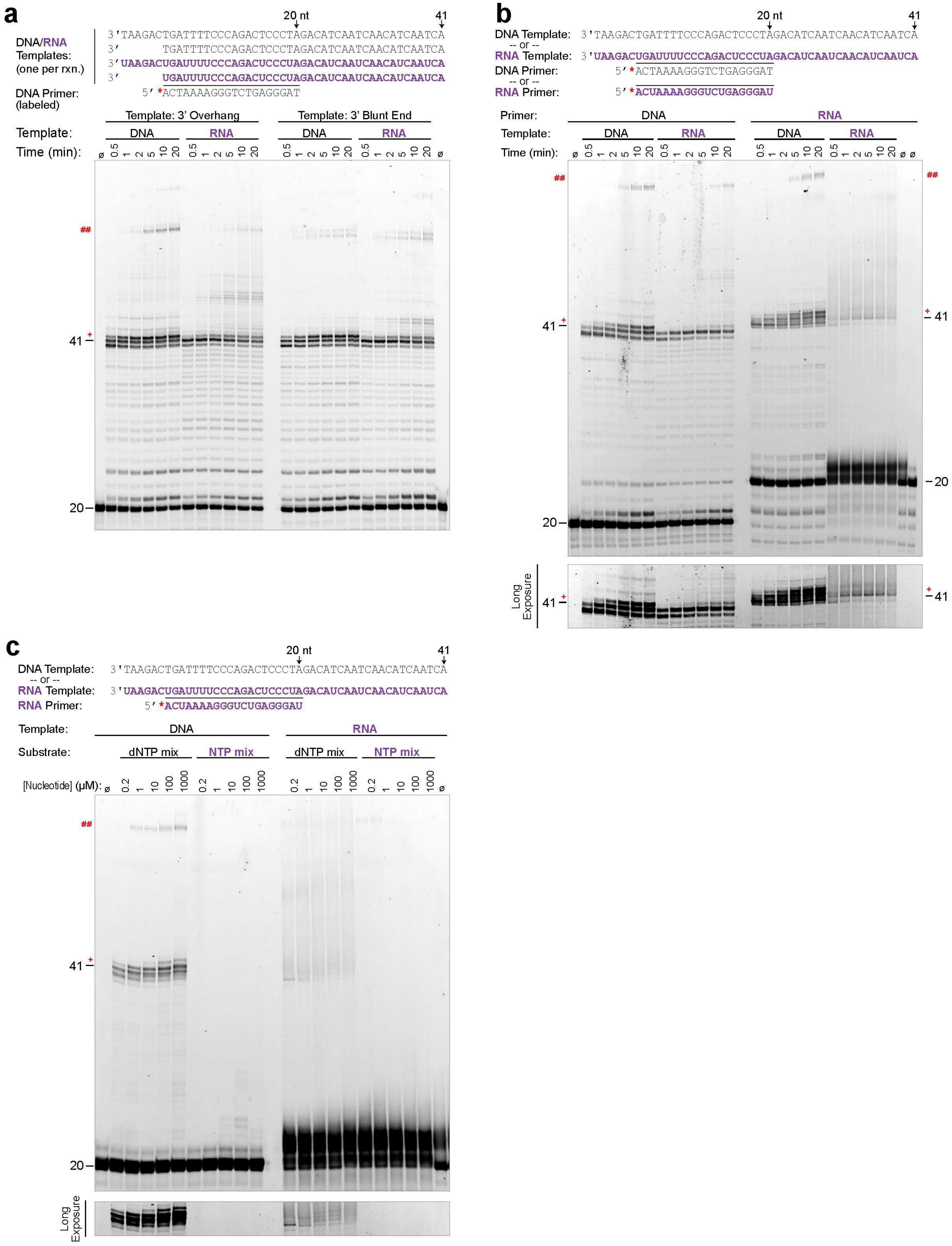

Supplementary Figure 3. Biochemical characterization of ORF2p core protein. **a**, ORFp core utilizes both RNA:DNA and DNA:DNA template:primer system for DNA synthesis at similar levels regardless of DNA or RNA nature of the template. While patterns of DNA synthesis differ slightly depending on the nature of the template,

ORF2p is agnostic about the 3' end of the template:primer pair, showing no difference between a blunt template or a 3' overhang. **b**, Denaturing PAGE migration pattern of the reaction products of the time course of dNTP incorporation along DNA and RNA templates using 20-nucleotide (nt) DNA or RNA primers. ORF2p core functions as an efficient DNA polymerase on all template:primer combinations. RNA priming on an RNA template is reduced but remains significant, with shows time-dependent formation of the full template-length (FTL) reaction products, more evident on the long exposure (below). For sequencing gel polymerase assays, ORF2p core was pre-incubated with pre-annealed template and labeled primer in EDTA-containing buffer and DNA synthesis was initiated by the addition of $MgCl_2$. Zero reaction lanes (left and right most) illustrates the migration pattern of template:primer pairs in the absence of reaction. FTL (41 nt) and primer (20 nt) are indicated. Non-templated addition of nucleotides (NTA) is marked by plus (+) and template jumping/switching products are labeled with hashes (##). RNA primers migrate slower than DNA primers on the denaturing PAGE due to differences in charge-to-mass ratio, and the denaturing conditions (95°C, formamide, 8 M urea) do not fully denature the RNA primer/RNA template. Scanned gel images are cropped and corrected for distortion artifacts with contrast uniformly increased to facilitate the visualization of minor products. **c**, RNA synthesis is strongly selected against, as indicated by nucleotide (dNTP or NTP) incorporation activity of LINE-1 RT on DNA or RNA using a RNA primer. Denaturing PAGE migration pattern of the reaction products generated after 5 minutes of dNTP or NTP incorporation along DNA and RNA templates using 20-nt primers. Gels straightened for clarity. Original scans are provided in a Source Data File.

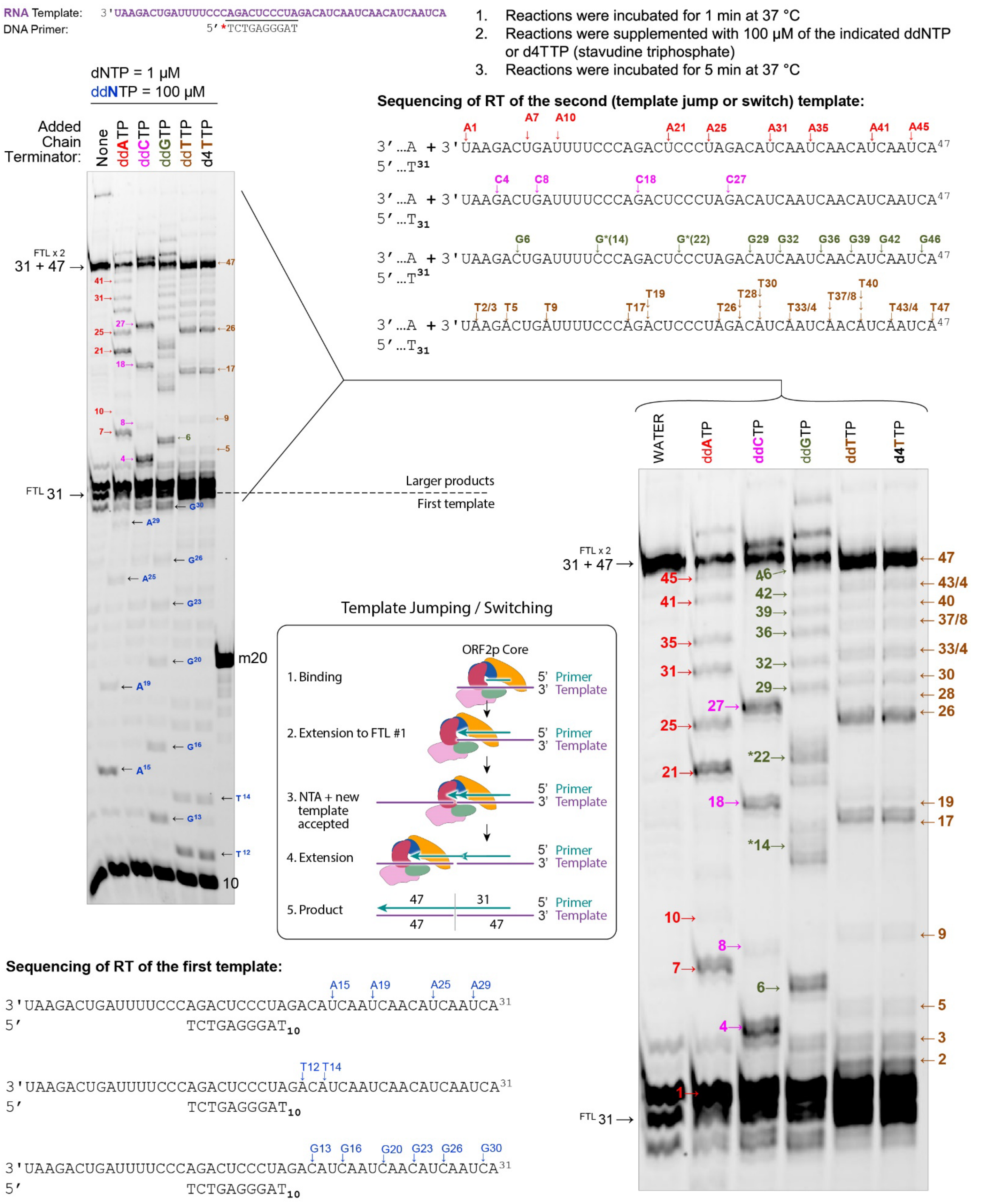

Supplementary Figure 4. Sanger sequencing-like reaction confirms high molecular weight reverse transcriptase products are template jumps/switches. Template jumping/switching activity (schematic inset) entails continued cDNA synthesis after the end of a first template has been reached by incorporation of a second

acceptor template, resulting in a product that is a concatemer of two templates (or more, with repeated events)[142,193]. Template jumping and switching are similar but differ in that template jumps are facilitated by short (1-3 nt) microhomology that may be created by NTA, whereas template switches are blunt[139-141,194]. This activity for ORF2p is confirmed by Sanger sequencing-like reactions, where *in vitro* polymerase reactions were conducted on DNA:DNA template:primers for 1 min and then continued for 5 min in 100-fold excess chain terminating dideoxy nucleotides (ddATP, ddTTP, ddCTP, ddGTP, d4T) as indicated. Complete Sanger sequencing of previously observed high molecular weight products confirms these do represent *bona fide* template jumps. Expected incorporation positions for ddATP, ddTTP, ddCTP, ddGTP and subsequent terminations for the first template (bottom) and second template (top) after template jumping are annotated and enlarged in inset. ORF2p core was preincubated with a template:primer for one minute at 37 °C with a dNTP mixture 1 uM supplemented with 100 uM ddNTP as labelled in 25 mM Tris-HCl (pH8) buffer, 50 mM NaCl, and 0.25 mM EDTA. Addition of d4T-TP, which is incorporated similarly to ddTTP, confirms the specificity of incorporation. Scanned gel images are cropped and corrected for distortion artifacts with contrast uniformly increased to facilitate the visualization of minor products. (*, Cy5 label). Original scans are provided in a Source Data File.

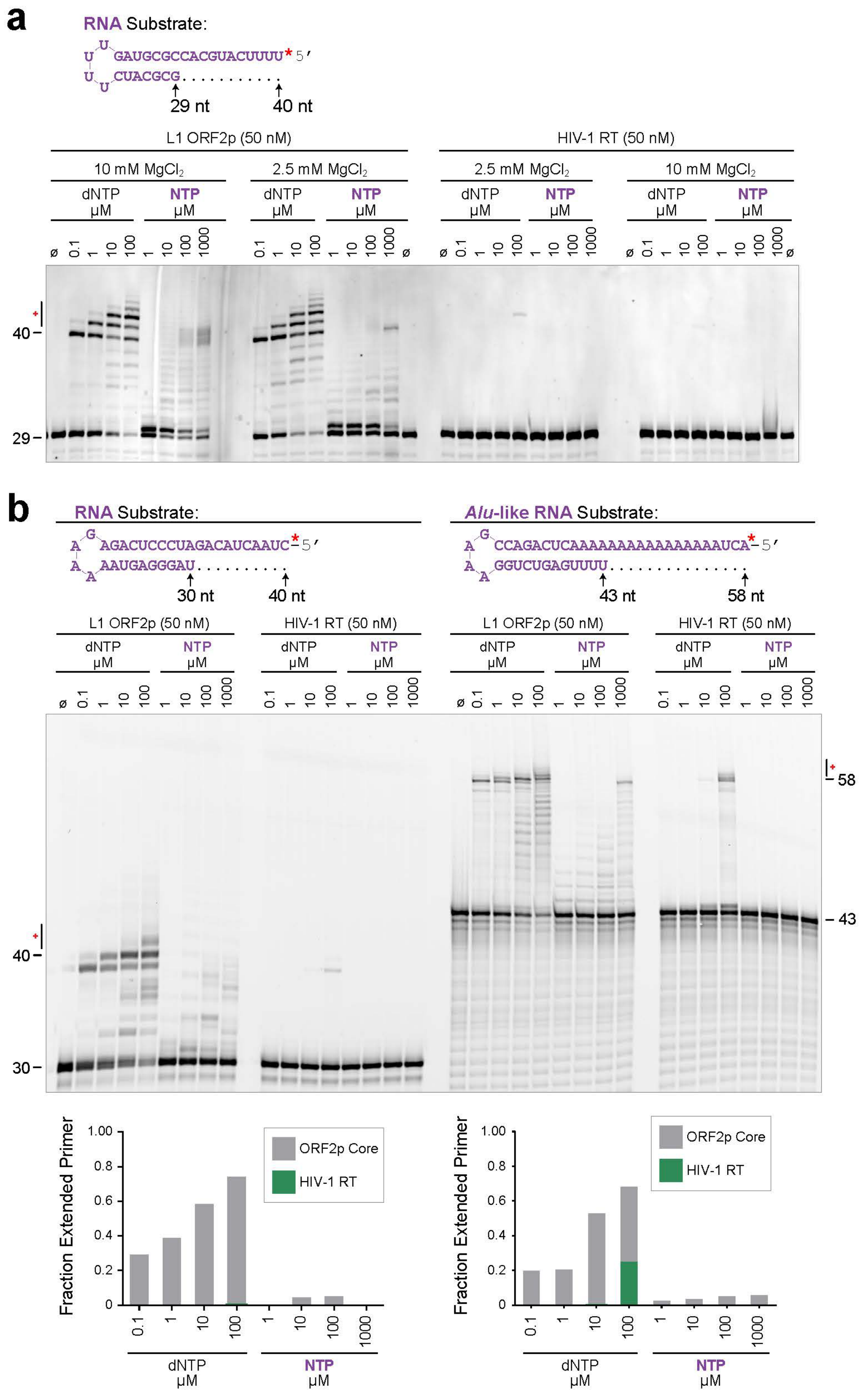

Supplementary Figure 5. ORF2p priming and extension on hairpin RNA substrates. L1 ORF2p Core RT vs HIV-1 RT. **a**, Hairpin substrate previously published in a SARS-CoV-2 study[195] and is 5' labeled with FAM. Two concentrations of initiating $MgCl_2$ were tested, and the nearly identical results with both establish that 2.5 mM $MgCl_2$ is not limiting for either enzyme. 50 nM hairpin substrate, incubated for 10 min at 37°C. Gel straightened for clarity. **b**, RNA substrate derived from previous biochemical experiments (left) and an Alu-like uridylated substrate (right). Initiated with 2.5 mM $MgCl_2$ and incubated 10 min at 37°C. Quantitation of each substrate (bar graphs n=1) are below the corresponding section on the gel, revealing extension by L1 that is ~100,000-fold (left) and ~1000-fold (right) more efficient than HIV-1. Gel straightened for clarity. Original scans are provided in a Source Data File.

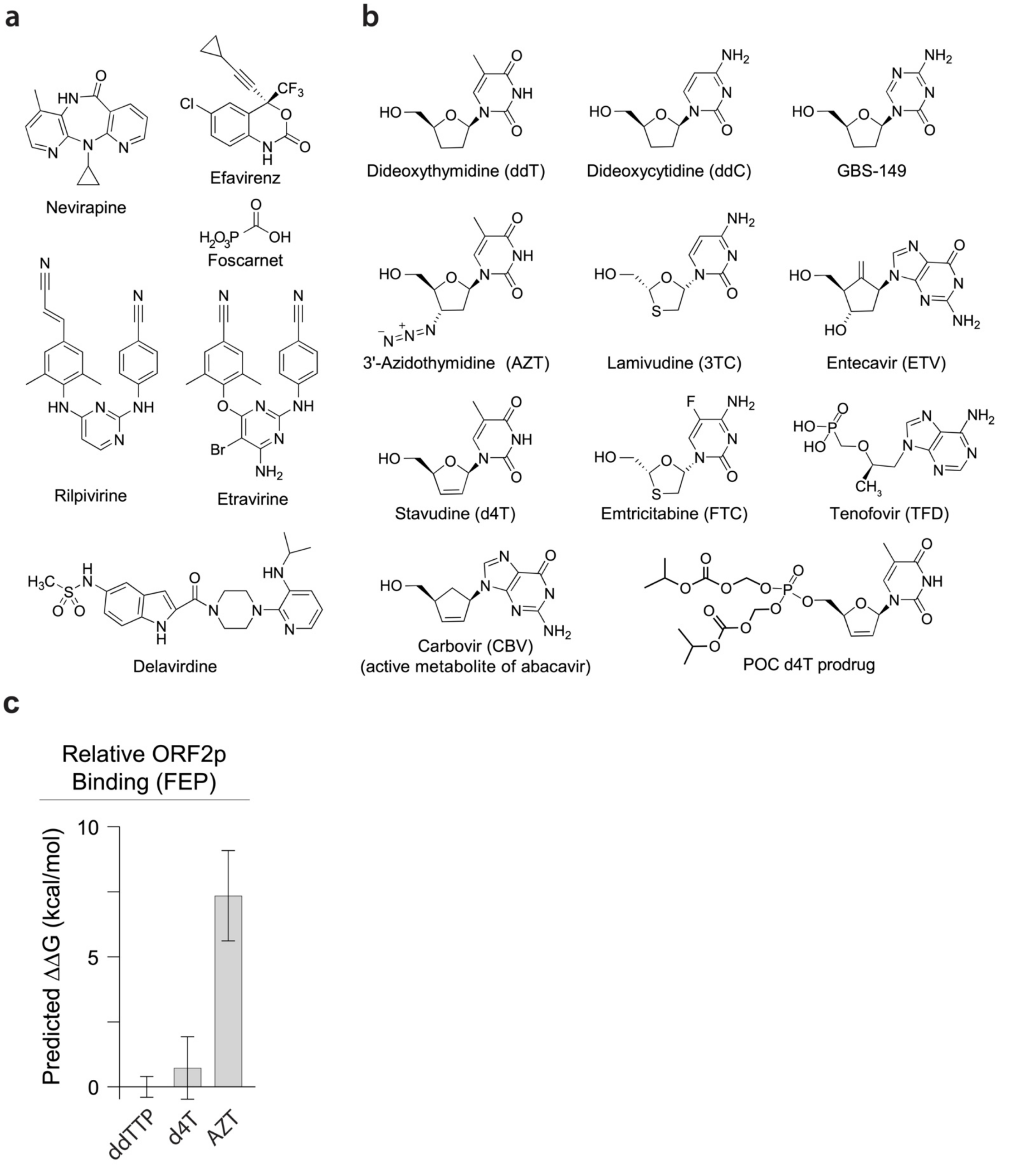

Supplementary Figure 6. NRTI and NNRTI reverse transcriptase inhibitors. **a-b**, Chemical structures of NNRTI (b) and NRTI (c) compounds used HTRF inhibition assays of ORF2p. **c**, Results of relative binding free energy calculations by free energy perturbation (FEP) for ddTTP, d4T, and AZT, based on the dTTP structure. Predicted binding $\Delta\Delta G$ values are relative to ddTTP, and error bars are ± cycle closure error; this error is dependent on the map of transformations that contains 4 ligands and 5 edges (10 simulations total) to reach the baseline state (e.g., different paths to go from ddTTP to d4T, for example through dTTP in this case).

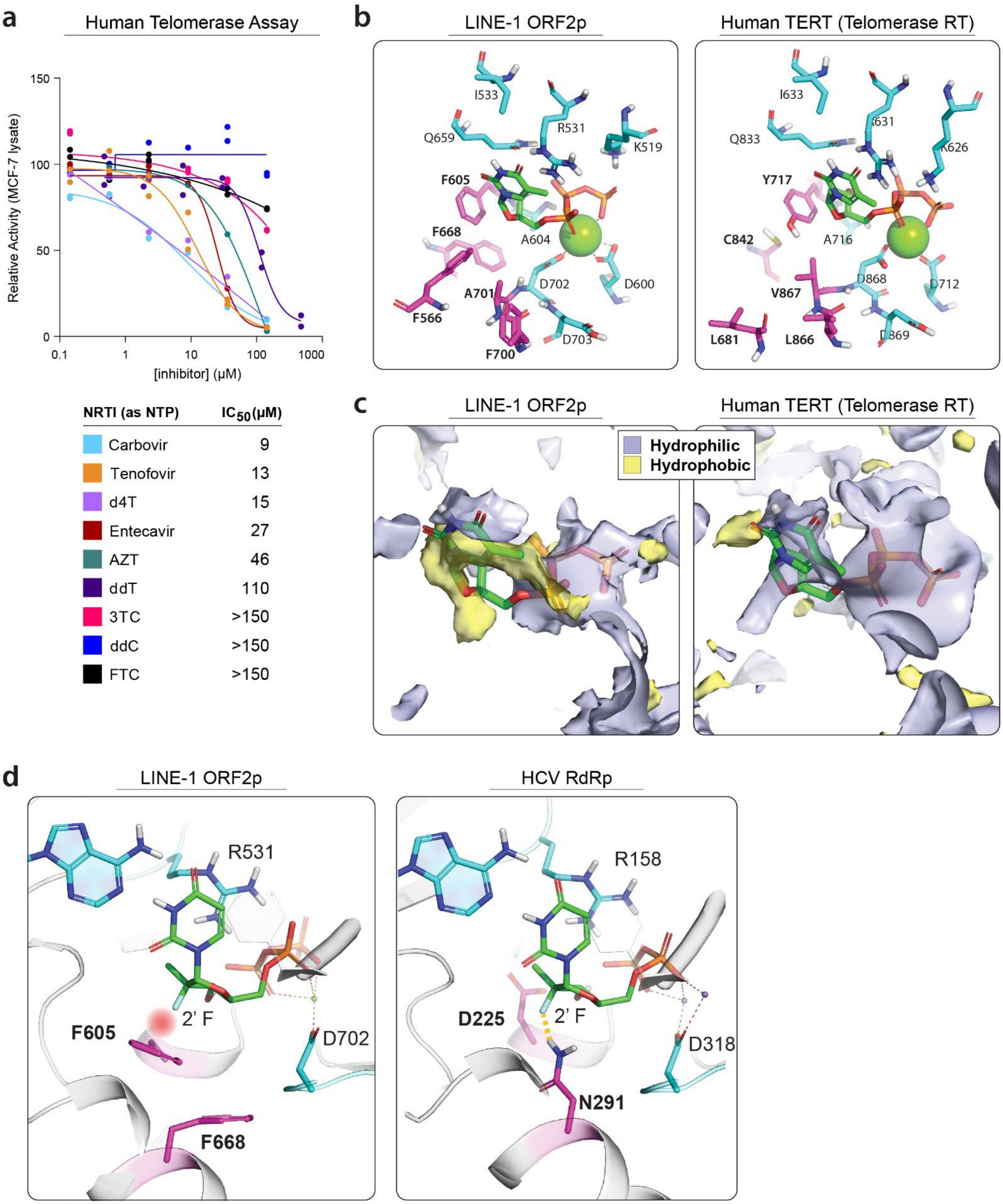

Supplementary Figure 7. TERT inhibition and comparative modeling of the ORF2p active site. **a**, Inhibition of human telomerase RT (TERT) by NRTI triphosphates in a biochemical assay in MCF-7 cell lysate (n=1-2 biologically independent samples as indicated, representative of two independent experiments). **b**, Active site of L1 RT bound to dTTP (left) and model of human TERT active site bound to dTTP (right), based on cryo-EM TERT structure (PDB: 7QXA). Identical residues are colored in cyan and residues that differ are colored in magenta.

c, SiteMap analysis of the L1 RT (left) and TERT (right) active sites showing the hydrophilic (teal) and hydrophobic (yellow) environments of the active sites. **d**, Model of sofosbuvir bound to L1 RT active site (left) and crystal structure of HCV RdRP bound to sofosbuvir (PDB: 4WTG, right). Note the clash between F605 in L1 and the 2'-F of the ligand. The equivalent position in HCV RdRP is D225, which provides sufficient space for the 2'-group. Additionally, N291 in HCV RdRP is within hydrogen-bonding distance of the of the 2' group while equivalent residue in L1 RT is F668, which precludes hydrogen bond formation.

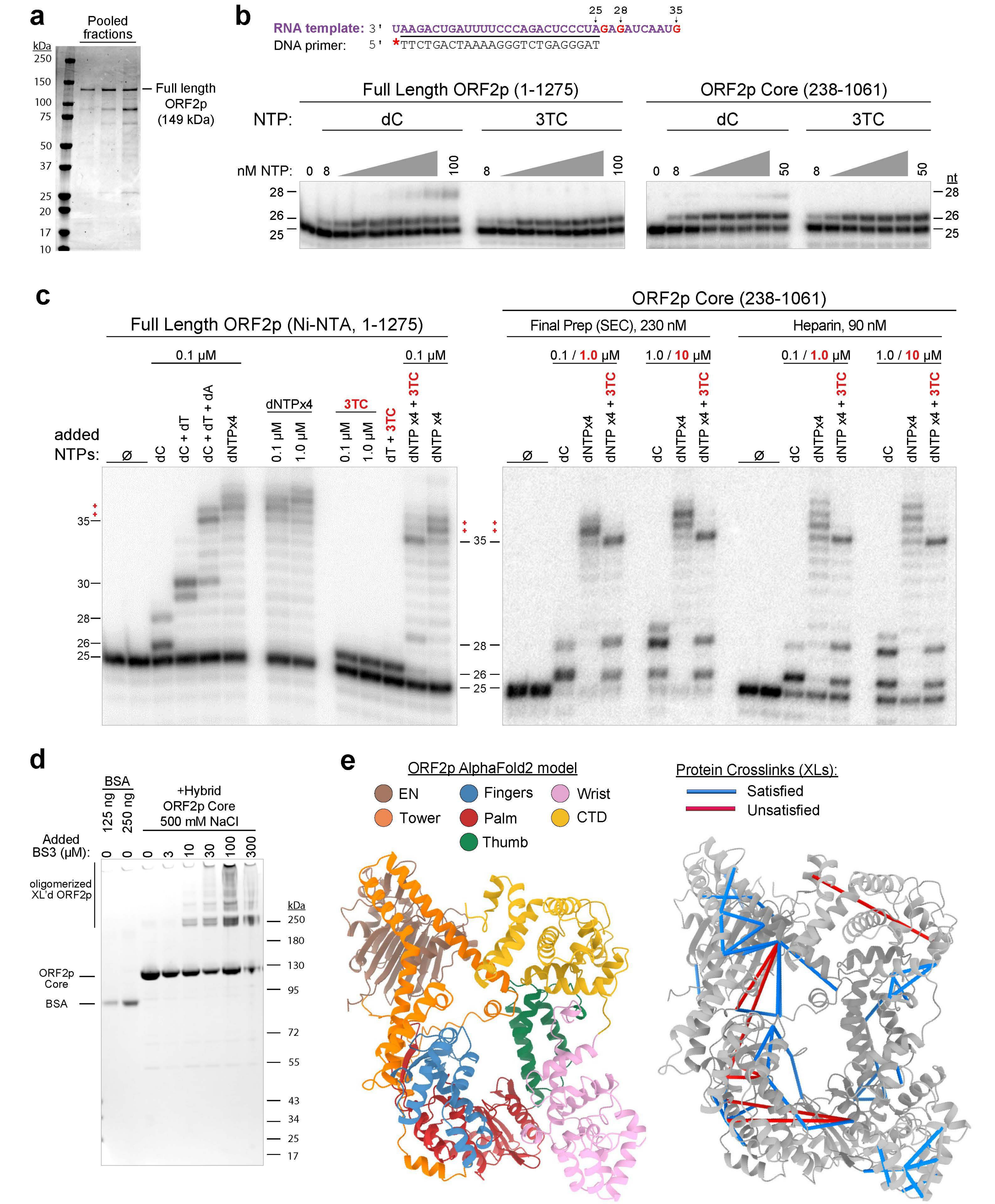

Supplementary Figure 8. Activity and inhibition of ORF2p full-length vs. core and crosslinking mass spectrometry (XL-MS) of ORF2p. **a**, Representative Coomassie stained SDS-PAGE of full-length ORF2p-C-His8 purified by Ni-NTA affinity and used for polymerase assays; this is expressed as a fusion polyprotein

containing N-terminal HERV-K and TEV proteases followed by TEV cleavage site, resulting in a single N-terminal glycine scar. **b**, Gels and template:primer system corresponding to single nucleotide incorporation data in **Fig. 3b**. Asterisk (*) ^{32}P-labeled 5'-end of the primer**. c**, Full length ORF2p and ORF2p core are compared in single nucleotide incorporation and inhibition experiments with the indicated nucleoside triphosphates and 3TC triphosphate; 'dNTPx4' is a mix of all four standard dNTPs. Full length ORF2p (purity insufficient to accurately determine concentration) produces similar reaction products and shows similar activity and inhibition to both partially-purified (Heparin) and fully-purified (after SEC) ORF2p core. **d**, Representative Coomassie stained SDS-Page of BS3-crosslinked ORF2p core protein, following reaction with various concentrations of BS3 in the presence of an annealed RNA template:DNA primer duplex. While electrophoretic mobility of crosslinked monomers may be challenging to predict, higher molecular weight species not present in the starting material (0 µM BS3) are likely enriched in intermolecular XLs, rather than desired intramolecular XLs. Based on this criteria, 10 and 30 µM BS3 products analyzed by MS. **e**, 56 unique crosslinks from ORF2p core and full-length ORF2p mapped onto the AlphaFold2 model of ORF2p (used as a starting point for integrative modeling); 91% of experimental crosslinks are satisfied. Original scans are provided in a Source Data File.

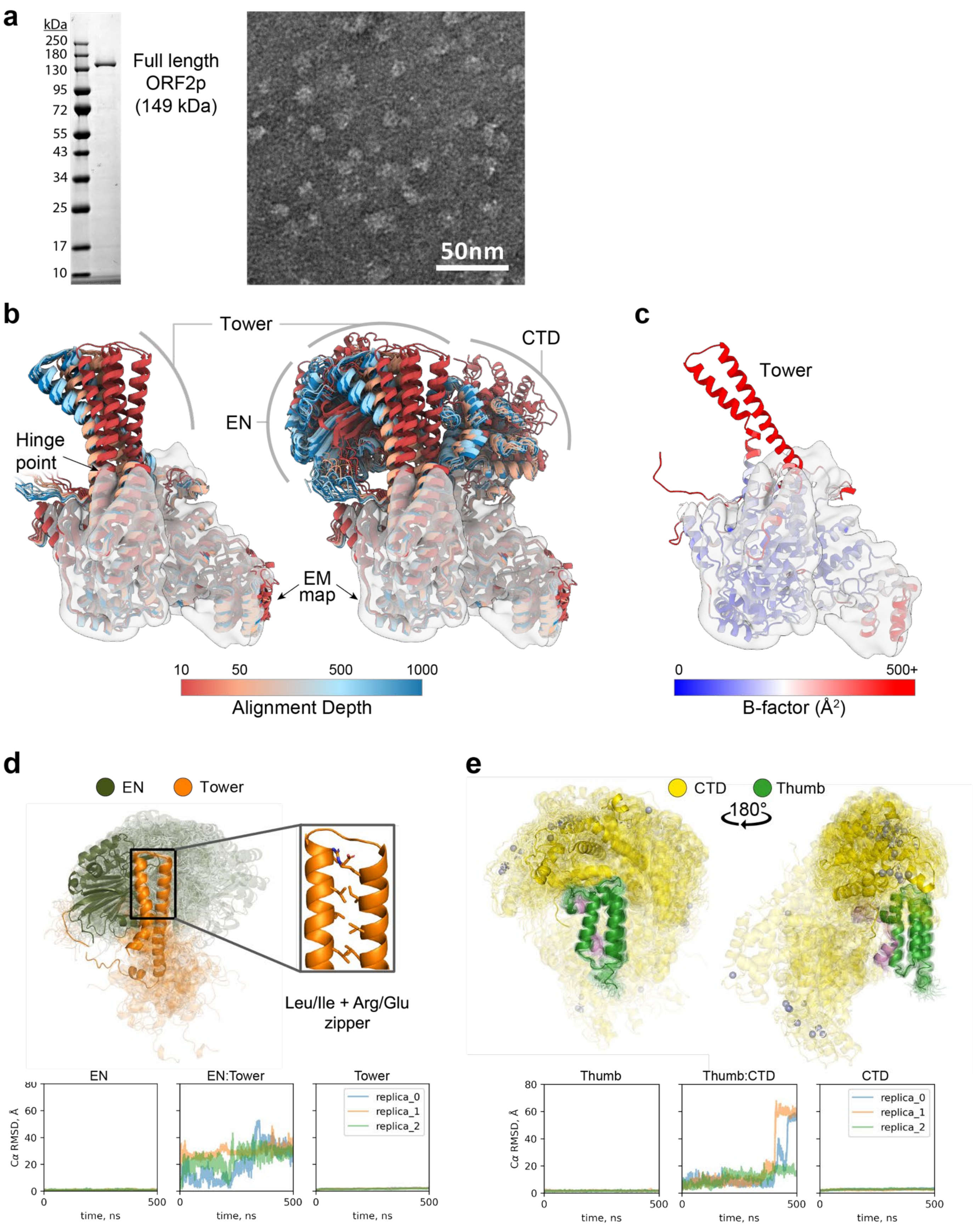

Supplementary Figure 9. Full-length ORF2p analyzed by EM and simulations. **a**, Representative Coomassie stained SDS-PAGE (left) and negative stain TEM (right) of full length, monodisperse C-3xFlag ORF2p used for structural analysis. **b,** AlphaFold2 predicts flexibility (or larger uncertainty) of EN, Tower, and CTD positions. **c,**

MD simulations of the *apo* protein show large flexibility of regions not resolved in the *apo* cryo-EM map; Wrist flexibility is also in agreement with a lower resolution of that region of the map and differences between maps from different techniques. **d,** Snapshots of MD simulations and RMSD plots of EN:Tower system show the stability of isolated EN and Tower in contrast with a large variability of pairwise orientations. **e**, Snapshots of MD simulations and RMSD plots of Thumb:CTD system similarly show the stability of isolated Thumb and CTD. The Thumb:CTD complexes are more stable, with trajectories showing late dissociation.

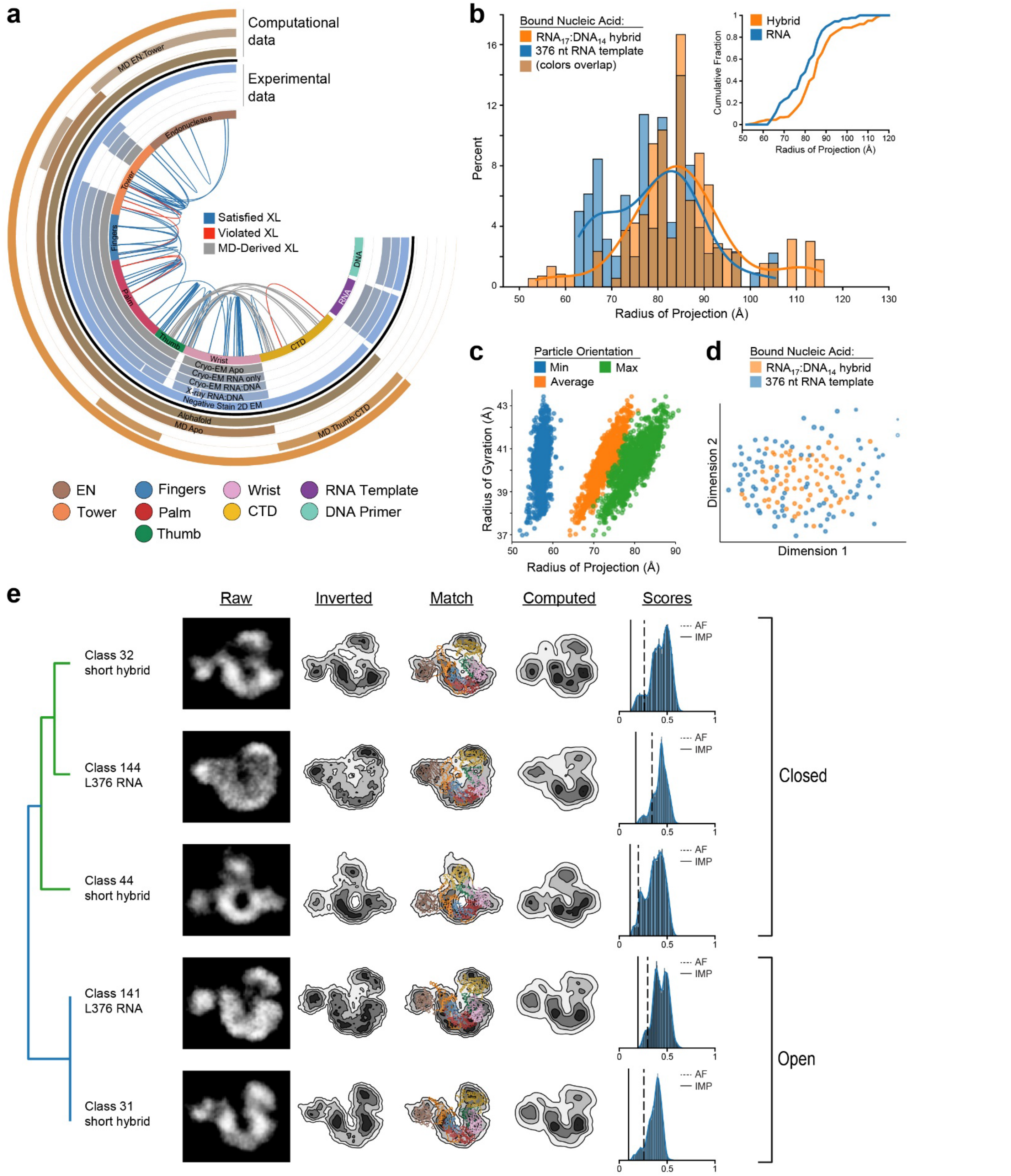

Supplementary Figure 10. Summary of integrative modeling, validation, and clustering of structural classes. a, Mapping of all experimental and computational data used for integrative modeling. **b**, Comparison of particle radii between the two negative stain EM analyses show a larger proportion of classes with smaller radii when ORF2p is bound to a long template RNA (376 nt) than seen when bound to a short hybrid (RNA_{17}:DNA_{14}), and a two-sample Kolmogorov-Smirnov test of 1000 random samples from each distribution shows the two

distributions are significantly different ($p=10^{-28}$), with the RNA-only particles smaller, highlighted by the inset cumulative distribution function (CDF) plot. **c**, Validation of radius of projection in 2D class averages as a metric of particle radius (radius of gyration), as the relation between the radius of projection and the radius of gyration of a model is non-linear. For some specific orientations of particles (Min) the radius of projection can be small and almost independent of the radius of gyration, however the average and maximum (Max) radii of projection show a strong linear correlation with the radius of gyration of a model ($r=0.82$ (average radius) and $r=0.88$ (maximum radius), $p<10^{-38}$ for both, two-tailed Pearson correlation). **d**, Multi-dimensional scaling comparison of 2D classes from negative stain EM of ORF2p bound to a short RNA_{17}:DNA_{14} hybrid or long (376 nt) L1 template RNA shows overlap in many classes from both but key differences. **e**, Hierarchical clustering of structures from RNA template- and RNA:DNA hybrid-bound class averages representing closed and open states. Raw 2D class averages, determined by k-means clustering, their inverted contour plots, superpositions with best-matching structure, contour plots of generated projections, and distribution of scores (lower is closer match) for all orientations of 101-best-matching models.

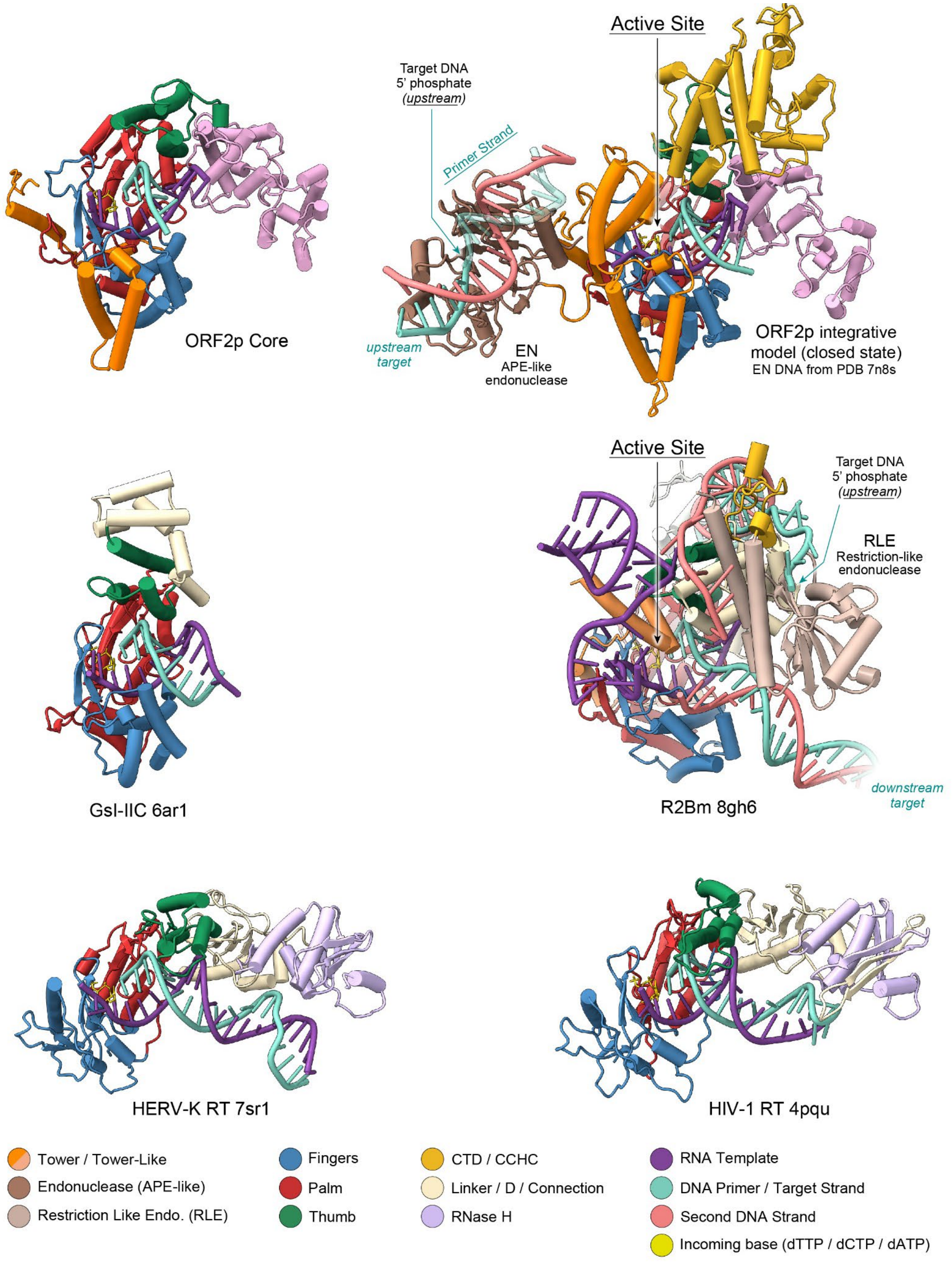

Supplementary Figure 11. Structures of RT-containing enzymes compared in this study. Structural

comparison of reverse transcriptase enzymes used for sequence alignment, aligned by palm superposition and viewed from the identical angle, colored by domain/subdomain, with bound RNA:DNA hybrids and incoming dNTP aligned. ORF2p is the largest enzyme and is shown in the closed state integrative model (Class 15) with bound DNA in EN colored with the primer strand, which would be passed into the active site during TPRT, shown transparent starting at the scissile bond. The endonuclease-cut target DNA is on opposite sides of the active site for ORF2p EN and R2 RLE, shown in more detail in Extended Data Figure 4.2. The overall arrangement of the fingers-palm-thumb is most similar between ORF2, R2, and GSI-IIC[196]; HIV-1 RT[197] and HERV-K RT[151] are more distinct from this group but are highly similar to each other. All five enzymes contain C-terminal domains that contact the downstream template:primer; The GSI-IIC D domain makes limited proximal contacts, ORF2p and R2Bm have distinct contacts from wrist and linker, and in HERV-K RT and HIV-1 RT, RNase H and the connection make distal contacts.

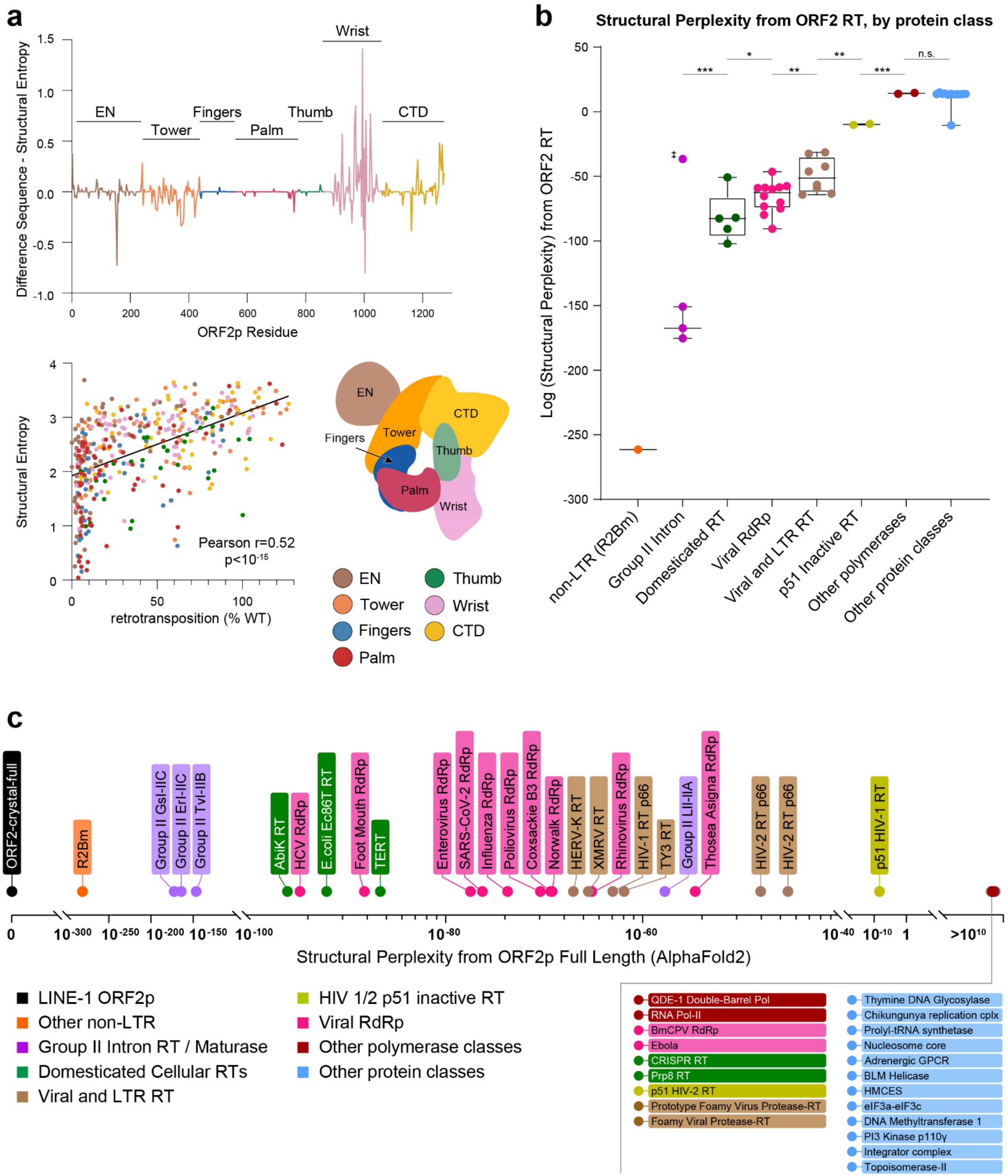

Supplementary Figure 12. Structural evolutionary analysis of ORF2p and its domains. a, (top panel) The difference between sequence and structural entropy based on a multiple sequence/structure alignment is plotted per residue over the length of ORF2p. There is very little difference within the RT domain (fingers-palm-

thumb), which is also the region with the lowest entropy and thus highest conservation. The largest differences are seen in wrist, tower, and CTD. (bottom panel) Plotting structural entropy vs retrotransposition in a scanning trialanine mutagenesis screen shows strong correlation between the two metrics; two-tailed Pearson correlation. **b**, Comparison of structural perplexity from ORF2p to the different classes of proteins; each data point represents the perplexity from ORF2p RT crystal of one protein (see **Supplementary Table 3** for the complete list); bounds of boxes are 25th and 75th percentiles, line represents the mean, and whiskers encompass all points used in comparisons. * $p<0.05$; ** $p<0.01$; *** $p<0.001$, two-tailed t-tests; ‡ the outlying group II intron from *Lactobacillus lactis* was not included in the comparison (n=3 group II Intron, n=5 domesticated RT, n=12 RdRP, n=8 viral/LTR RT, n=2 p51 inactive RT, n=2 other polymerase, n=12 other protein classes; sequential p values are as follows, starting from comparison between group II introns and domesticated RTs, and culminating between other polymerases and other protein classes: 0.0006, 0.04, 0.009, 0.004, 0.0005, 0.6). **c**, Structural perplexities of all proteins in the set from full-length ORF2p (AlphaFold2 model), as shown for the RT domain in **Fig. 5d**.

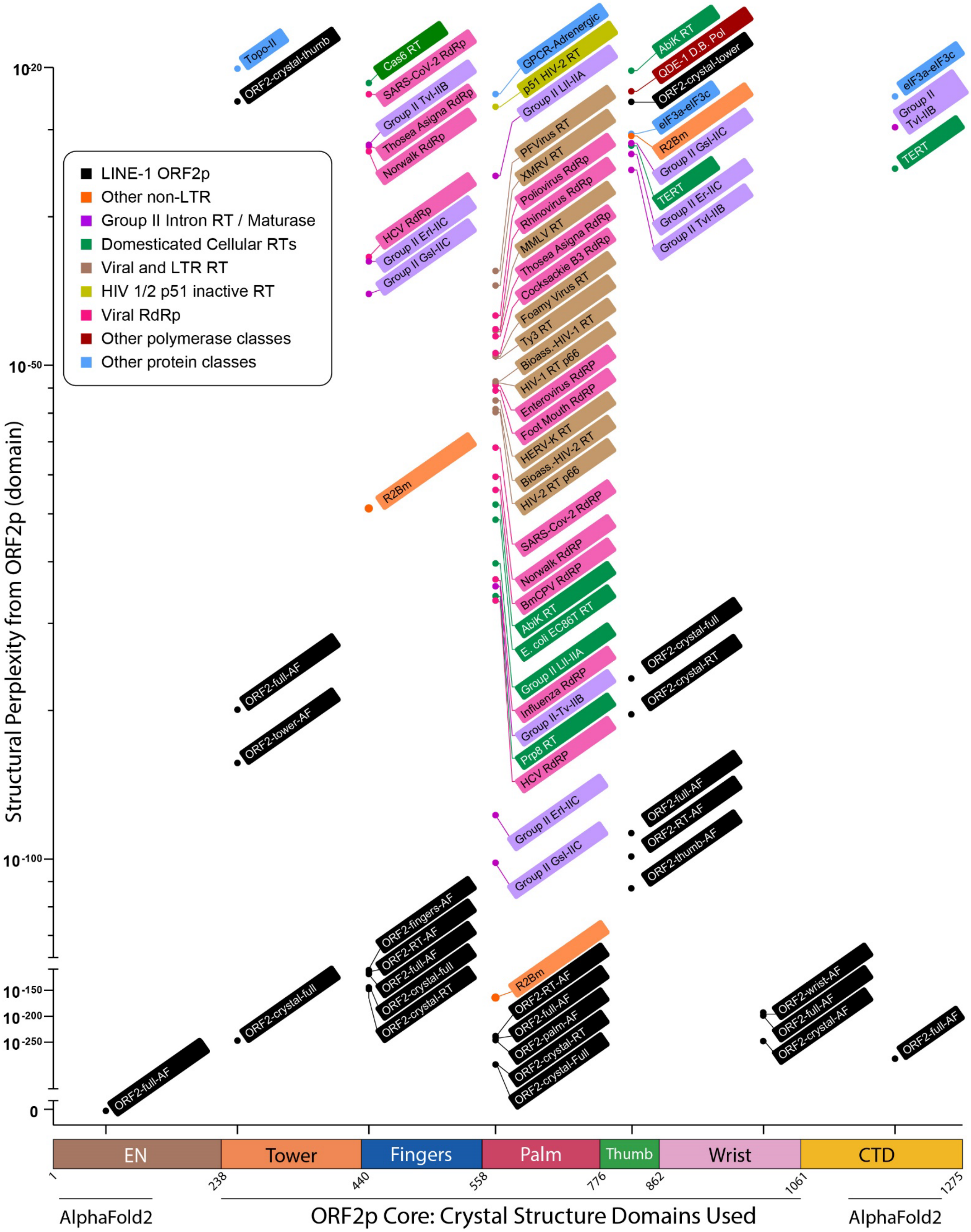

Supplementary Figure 13. Structural perplexity of ORF2p domains relative to the other proteins in the curated set. The seven domains and subdomains of ORF2p are plotted relative to the set of 50 proteins and

to each other. Where available, both the crystal coordinates and those from AlphaFold2 were compared (ORF2-crystal-full is 238-1061; ORF2-full-AF is 1-1275, etc.). Proteins with perplexity < 10^{-20} are shown; above this value, in most groups the “other protein classes”, which may generally be viewed as ‘decoys’ start to score. Outside of this to ORF2p itself, the EN, tower, and wrist domains all have no significant hits in this set; CTD has very weak similarity to TERT and the Group IIB intron from *Thermosynechococcus vestitus*. The ancestral palm subdomain has very low perplexity with many polymerases in the set and recapitulates many of the relationships seen with the full crystal structure: ORF2p palm is predicted to be most similar to the other non-LTR transposon, R2Bm, followed by Group II mobile introns, HCV and influenza RdRPs, and domesticated cellular RTs, including PRP8 and TERT, followed more distantly by retroviral RTs. Again, the inactive p51 conformations of HIV-1/2 RT are predicted to be much more distant from ORF2p than the active p66 conformations, which are identical in sequence up to a deletion. The fingers and then thumb subdomains are each predicted to be less similar than palm to smaller numbers of these proteins, but in roughly similar orders, although interestingly in the thumb R2Bm is predicted to be slightly less similar to ORF2p than some of the evolutionarily more distant proteins such as TERT and Group II introns.

Supplementary Tables

Supplementary Table 1. Summary of identified crosslinks from crosslinking mass spectrometry

Absolute Position 1	Absolute Position 2	Detected Peptide Peptide1(XL position)-Peptide2(XL position)	Detected In ORF2p
469	541	KENFRPISLMNIDAKILNK(15)-KSPGPDGFTAEFYQR(1)	Both full length & core
306	358	FIALNAYKR(8)-TLQKINESR(4)	Both full length & core
636	654	AIYDKPTANIILNGQK(5)-LEAFPLKTGTR(7)	Both full length & core
306	313	SKIDTLTSQLK(2)-FIALNAYKR(8)	Both full length & core
354	306	EIETQKTLQK(6)-FIALNAYKR(8)	Both full length & core
636	628	AIYDKPTANIILNGQK(5)-LGIDGTYFKIIR(9)	Both full length & core
786	793	ENYKPLLK(4)-EIKEETNK(3)	Both full length & core
372	358	INKIDRPLAR(3)-TLQKINESR(4)	Both full length & core
469	545	KSPGPDGFTAEFYQR(1)-ILNKILANR(4)	Both full length & core
322	348	IDTLTSQLKELEK(9)-IRAELKEIETQK(6)	Both full length & core
1010	1017	DKIDKWDLIK(5)-LKSFCTAK(2)	Both full length & core
313	342	SKIDTLTSQLK(2)-RQEITKIR(6)	Both full length & core
126	163	VNKDTQELNSALHQADLIDIYR(3)-FIKQVLSDLQR(3)	Full length
692	965	WIKDLNVKPK(3)-GIQLGKEEVK(6)	Full length
1005	1010	IDKWDLIK(3)-AMATKDK(5)	Full length
764	786	QTESQIMGELPFTIASKR(17)-ENYKPLLK(4)	Full length
793	846	TTLKFIWNQK(4)-EIKEETNK(3)	Full length
970	1015	WDLIKLK(5)-DLNVKPK(5)	Full length
23	348	TGSNSHITILTLNINGLNSAIKR(22)-AELKEIETQK(4)	Full length
1000	1007	DKIDKWDLIK(2)-TPKAMATK(3)	Full length
519	737	EGILPNSFYEASIILIPKPGR(18)-INVQKSQAFLYTNNR(5)	Full length
866	1089	NKAGGITLPDFK(2)-EDIYAAKK(7)	Full length
764	782	QTESQIMGELPFTIASKR(17)-ENYKPLLK(4)	Full length
251	358	STTWKLNNLLLNDYWVHNEMK(5)-TLQKINESR(4)	Full length
416	654	LEAFPLKTGTR(7)-EYYKHLYANK(4)	Full length
1010	1017	IDKWDLIK(3)-LKSFCTAK(2)	Full length
469	519	EGILPNSFYEASIILIPKPGR(18)-KSPGPDGFTAEFYQR(1)	Full length
778	786	ENYKPLLK(4)-DVKDLFK(3)	Full length
924	965	WIKDLNVKPK(3)-NKQWGK(2)	Full length
1123	1263	LSQEQKTK(6)-MAIIKK(5)	Full length
866	1070	NKAGGITLPDFK(2)-TNNPIKK(6)	Full length
313	354	SKIDTLTSQLK(2)-EIETQKTLQK(6)	Full length
992	1005	TLEENLGITIQDIGVGKDFMSK(17)-AMATKDK(5)	Full length
786	793	DLFKENYKPLLK(8)-EIKEETNK(3)	Full length
1070	1089	KTNNPIKK(7)-EDIYAAKK(7)	Full length
358	468	LNQEEVESLNRPITGSEIVAIINSLPTKK(28)-TLQKINESR(4)	Full length
992	1017	TLEENLGITIQDIGVGKDFMSK(17)-LKSFCTAK(2)	Full length
422	654	HLYANKLENLEEMDTFLDTYTLPR(6)-LEAFPLKTGTR(7)	Full length
764	778	QTESQIMGELPFTIASKR(17)-DVKDLFK(3)	Full length
1005	1017	LKSFCTAK(2)-AMATKDK(5)	Full length
965	975	TIKTLEENLGITIQDIGVGK(3)-WIKDLNVKPK(3)	Full length
32	358	LASWIKSQDPSVCCIQETHLTCR(6)-TLQKINESR(4)	Full length
831	880	FNAIPIKLPMTFFTELEK(7)-LYYKATVTK(4)	Full length
216	240	IKNLTQSR(2)-ALLSKCK(5)	Full length
251	387	STTWKLNNLLLNDYWVHNEMK(5)-EKNQIDTIK(2)	Full length
578	628	LGIDGTYFKIIR(9)-KSINVIQHINR(1)	Core
852	858	IAKSILSQK(3)-FIWNQKR(6)	Core
556	578	KSINVIQHINR(1)-IQQHIKK(6)	Core
628	654	LGIDGTYFKIIR(9)-LEAFPLKTGTR(7)	Core
607	628	AFDKIQQPFMLK(4)-LGIDGTYFKIIR(9)	Core
545	556	ILNKILANR(4)-IQQHIKK(6)	Core
858	866	NKAGGITLPDFK(2)-IAKSILSQK(3)	Core
397	545	NDKGDITTDPTEIQTTIR(3)-ILNKILANR(4)	Core
578	654	KSINVIQHINR(1)-LEAFPLKTGTR(7)	Core
416	545	EYYKHLYANK(4)-ILNKILANR(4)	Core
322	348	IDTLTSQLKELEKQEQTHSK(9)-AELKEIETQK(4)	Core
358	387	REKNQIDTIK(3)-TLQKINESR(4)	Core
358	416	EYYKHLYANK(4)-TLQKINESR(4)	Core
469	519	EGILPNSFYEASIILIPKPGRDTTK(18)-KSPGPDGFTAEFYQR(1)	Core
615	628	IQQPFMLKTLNK(8)-LGIDGTYFKIIR(9)	Core

Supplementary Table 2. Summary of integrative modeling data.

1. Model Composition	
PDBDEV ID	PDBDEV_00000211
Entry Composition	LINE-1 ORF2p: Chain A (1275 residues)
Datasets used for modeling	- De Novo model, AlphaFold DB: O00370<br>- Mass Spectrometry data, PRIDE: PXD038615<br>- CX-MS data, Linker name and number of cross-links: BS3, 15 cross-links<br>- CX-MS data, Linker name and number of cross-links: BS3, 11 cross-links<br>- CX-MS data, Linker name and number of cross-links: BS3, 30 cross-links<br>- EM raw micrographs, EMPIAR: 11556<br>- 3DEM volume, EMDB: 40856<br>- 2DEM class average, File<br>- De Novo model, ModelArchive: ma-xlzzy
2. Representation	
Resolution	Coarse-grained: 1 residue(s) per bead
Number of rigid bodies, flexible units	15, 14
Rigid bodies	A: 8-237, 250-258, 260-277, 284-310, 313-352, 353-359, 362-370, 375-381, 393-849, 857-862, 864-868, 873-955, 960-1030, 1033-1061, 1068-1275
Flexible units	A: 1-7, 238-249, 259-259, 278-283, 311-312, 360-361, 371-374, 382-392, 850-856, 863-863, 869-872, 956-959, 1031-1032, 1062-1067
Structural coverage (rigid bodies)	95%
3. Restraints	
Physical principles	- Sequence connectivity<br>- Excluded volume
Experimental data	- 1 unique CrossLinkRestraint: BS3, 15 crosslinks<br>- 1 unique CrossLinkRestraint: BS3, 11 crosslinks<br>- 1 unique CrossLinkRestraint: BS3, 30 crosslinks<br>- 15 unique PredictedContactRestraint: Distance: 27.0<br>- 1 unique EM3DRestraint: Gaussian mixture models
4. Validation	
Number of ensembles	1
Number of models in ensembles	1383
Number of deposited models	159
Model precision (uncertainty of models)	12.871Å
Data quality	Data quality has not been assessed
Model quality: assessment of excluded volume	Satisfaction: 99.60-99.61%
Fit to data used for modeling	Satisfaction of crosslinks: 85.71-92.86%
Fit to data used for validation	- Per-model EM2D scores (0-1, lower-better): 0.04-0.33
5. Methodology and Software	
Method	Sampling
Name	Replica Exchange Gibbs sampling, based on Metropolis Monte Carlo
Description	20 replicas; 3 runs; 10000 models per run
Number of computed models	30000
Software	- IMP PMI module (version 2.18.0)<br>- Integrative Modeling Platform (IMP) (version 2.18.0)

Supplementary Table 3. Evolutionary analysis curated protein set

PDB ID	Description	Protein Type	RT/RT-like Chain
8C8J	ORF2p-Crystal (homo sapiens)	Non-LTR	A
(AF) O00370	ORF2p-AlphaFold2 (homo sapiens)	Non-LTR	A
8gh6	R2Bm (R2 from *Bombyx mori*)	Non-LTR	A
5g2x	Group IIA intron from *Lactococcus lactis* (LlI-IIA)	Group-II Intron	C
6ar1	Group IIC intron from *Geobac. stearothermophilus* (GsI-IIC)	Group-II Intron	A
6me0	Group IIB intron from *Thermo. vestitus* (TvI-IIB)	Group-II Intron	C
7uin	Group IIC intron maturase from *Eubacterium rectale* (ErI-IIC)	Group-II Intron	D
7kqn	TERT	Domesticated RT	A
7r06	AbiK RT	Domesticated RT	A
7v9u	E.coli Ec86T RT	Domesticated RT	B
4i43	Prp8 RT	Domesticated RT	B
6tz2	BmCPV RdRp	RdRp-dsRNA	A
6qct	Influenza RdRp	RdRp-minus	B
3ol7	Poliovirus RdRp	RdRp-plus	A
4k4y	Coxsackie B3 RdRp	RdRp-plus	A
4k50	Rhinovirus RdRp	RdRp-plus	A
4wta	Hepatitis C Virus (HCV) RdRp	RdRp-plus	A
5tsn	Norwalk virus RdRp	RdRp-plus	A
6kwr	Enterovirus RdRp	RdRp-plus	A
7aap	SARS-CoV-2 RdRp	RdRp-plus	A
7om7	Thosea Asigna RdRp	RdRp-plus	A
7yer	Ebola Protein L RdRp	RdRp-plus	A
2e9r	Foot-and-Mouth RdRp	RdRp-plus	X
7kfu	CRISPR RT	RT-Other	C
4ol8	Ty3 RT	RT-LTR	A
7sr6	HERV-K RT	RT-LTR	A
1mu2	HIV-2 RT p66	RT-Retrovirus	A
4hkq	XMRV RT	RT-Retrovirus	A
4mh8	MMLV RT	RT-Retrovirus	A
4pqu	HIV-1 RT p66	RT-Retrovirus	A
7ksf	Prototype Foamy Virus Protease-RT	RT-Retrovirus	A
7o0g	Foamy Viral Protease-RT	RT-Retrovirus	A
1mu2	p51 HIV-2 RT	Inactive Retrovirus	B
4pqu	p51 HIV-1 RT	Inactive Retrovirus	B
1mu2	HIV-2 RT Bioassembly	Retrovirus-Bioassembly	A,B
4pqu	HIV-1 Bioassembly	Retrovirus-Bioassembly	A,B
7y7q	QDE-1 Double-Barrel Pol	DdRp-RdRP	A
2r92	RNA Pol-II	DdRp	None
1aoi	Nucleosome core	'Negative' Control	None
1bgw	Topoisomerase-II	'Negative' Control	None
1h4s	Prolyl-tRNA synthetase	'Negative' Control	None
2rh1	GPCR-Adrenergic	'Negative' Control	None
3uo7	Thymine DNA Glycosylase	'Negative' Control	None
4u1c	eIF3a-eIF3c complex	'Negative' Control	None
6oe7	HMCES	'Negative' Control	None
7mez	PI3 Kinase p110γ	'Negative' Control	None
7pks	Integrator complex	'Negative' Control	None
7xi9	DNMT1 DNA Methyltransferase	'Negative' Control	None
7y38	Chikungunya replication complex	'Negative' Control	None
4cgz	BLM Helicase	'Negative' Control	None

Supplementary Table 4. Plasmids used

Plasmid	Description	Source
pAMS823	His6-MBP-3C-ORF2p (238-1061, ORFeus-Hs) in pET41	This study
pMT692	ORF2p-3C-3xF (1-1275, ORFeus-Hs) in pDARMO-PolH2.1 for insect cell expression	This study
pMT646	ORF2p-3xFlag (ORFeus-Hs, CMV promoter) in pCEP4 Puro	This study
pMT870	RT- (ORF2p D702Y) derivative of pMT646	This study
pMT1093	EN- (ORF2p double E43S D145N) derivative of pMT646	This study
pMT647	ORF2-only (no-ORF1) version of pMT646	This study
pLD564	L1RP ORF2p-3xFlag (CMV promoter) in pCEP4 Puro	Taylor et al. Cell 2013
pRT006.3	Bi-directional luciferase antisense intron (firefly fluc AI) retrotransposition reporter (ORFeus-Hs sequence) for sleeping beauty integration	This study
pCMV(CAT)T7-SB100	SB100X transposase expression	Dr. Zsuzsanna Izsvak
1GFP/RNase H1 D210N	Expresses GFP-tagged RNase H1 D210N in E. coli (Addgene #174448, a gift from Dr. Cimprich)	Dr. Karlene Cimprich

All plasmids are available from Addgene.

Supplementary Table 5. Affinity reagents used

**Affinity reagent**	**Type**	**Source**
Anti-Flag M2	Mouse monoclonal antibody	Sigma #F1804
Anti-ORF1 4H1	Mouse monoclonal antibody	Burns lab stock; available as Millipore MABC1152
Anti-ORF1 JH73	Rabbit monoclonal antibody	Gift from Dr. Jeff Han
dRNH1 (GFP-human RNase H1 27-286; d210N-His6)	RNA:DNA hybrid imaging reagent	Purified from *E. coli* expressing 1GFP/RNase H1 D210
Recombinant S9.6	Rabbit monoclonal antibody	Kerafast Kf-Ab01137-23.0
GFP tag Polyclonal Antibody	Rabbit polyclonal antibody	Life Technologies # 50430-2-AP

Supplementary References

78 McClintock, B. The origin and behavior of mutable loci in maize. *Proc Natl Acad Sci U S A* **36**, 344-355 (1950). https://doi.org/10.1073/pnas.36.6.344

79 Boeke, J. D., Garfinkel, D. J., Styles, C. A. & Fink, G. R. Ty elements transpose through an RNA intermediate. *Cell* **40**, 491-500 (1985). https://doi.org/10.1016/0092-8674(85)90197-7

80 Burns, K. H. & Boeke, J. D. Human transposon tectonics. *Cell* **149**, 740-752 (2012). https://doi.org/10.1016/j.cell.2012.04.019

81 Lander, E. S. *et al.* Initial sequencing and analysis of the human genome. *Nature* **409**, 860-921 (2001). https://doi.org/10.1038/35057062

82 Kazazian, H. H., Jr. & Moran, J. V. Mobile DNA in Health and Disease. *N Engl J Med* **377**, 361-370 (2017). https://doi.org/10.1056/NEJMra1510092

83 Taylor, M. S. *et al.* Dissection of affinity captured LINE-1 macromolecular complexes. *Elife* **7** (2018). https://doi.org/10.7554/eLife.30094

84 Khazina, E. *et al.* Trimeric structure and flexibility of the L1ORF1 protein in human L1 retrotransposition. *Nat Struct Mol Biol* **18**, 1006-1014 (2011). https://doi.org/10.1038/nsmb.2097

85 Martin, S. L. *et al.* LINE-1 retrotransposition requires the nucleic acid chaperone activity of the ORF1 protein. *J Mol Biol* **348**, 549-561 (2005). https://doi.org/10.1016/j.jmb.2005.03.003

86 Hohjoh, H. & Singer, M. F. Cytoplasmic ribonucleoprotein complexes containing human LINE-1 protein and RNA. *EMBO J* **15**, 630-639 (1996).

87 Mita, P. *et al.* LINE-1 protein localization and functional dynamics during the cell cycle. *Elife* **7** (2018). https://doi.org/10.7554/eLife.30058

88 Mathias, S. L., Scott, A. F., Kazazian, H. H., Jr., Boeke, J. D. & Gabriel, A. Reverse transcriptase encoded by a human transposable element. *Science* **254**, 1808-1810 (1991).

89 Feng, Q., Moran, J. V., Kazazian, H. H., Jr. & Boeke, J. D. Human L1 retrotransposon encodes a conserved endonuclease required for retrotransposition. *Cell* **87**, 905-916 (1996).

90 Cost, G. J. & Boeke, J. D. Targeting of human retrotransposon integration is directed by the specificity of the L1 endonuclease for regions of unusual DNA structure. *Biochemistry* **37**, 18081-18093 (1998). https://doi.org/10.1021/bi981858s

91 Ahl, V., Keller, H., Schmidt, S. & Weichenrieder, O. Retrotransposition and Crystal Structure of an Alu RNP in the Ribosome-Stalling Conformation. *Mol Cell* **60**, 715-727 (2015). https://doi.org/10.1016/j.molcel.2015.10.003

92 Taylor, M. S. *et al.* Affinity proteomics reveals human host factors implicated in discrete stages of LINE-1 retrotransposition. *Cell* **155**, 1034-1048 (2013). https://doi.org/10.1016/j.cell.2013.10.021

93 Kulpa, D. A. & Moran, J. V. Cis-preferential LINE-1 reverse transcriptase activity in ribonucleoprotein particles. *Nat Struct Mol Biol* **13**, 655-660 (2006). https://doi.org/10.1038/nsmb1107

94 Esnault, C., Maestre, J. & Heidmann, T. Human LINE retrotransposons generate processed pseudogenes. *Nat Genet* **24**, 363-367 (2000). https://doi.org/10.1038/74184

95 Wei, W. *et al.* Human L1 retrotransposition: cis preference versus trans complementation. *Mol Cell Biol* **21**, 1429-1439 (2001). https://doi.org/10.1128/MCB.21.4.1429-1439.2001

96 Doucet, A. J., Wilusz, J. E., Miyoshi, T., Liu, Y. & Moran, J. V. A 3' Poly(A) Tract Is Required for LINE-1 Retrotransposition. *Mol Cell* **60**, 728-741 (2015). https://doi.org/10.1016/j.molcel.2015.10.012

97 Dai, L., Taylor, M. S., O'Donnell, K. A. & Boeke, J. D. Poly(A) binding protein C1 is essential for efficient L1 retrotransposition and affects L1 RNP formation. *Mol Cell Biol* **32**, 4323-4336 (2012). https://doi.org/10.1128/MCB.06785-11

98 Flasch, D. A. *et al.* Genome-wide de novo L1 Retrotransposition Connects Endonuclease Activity with Replication. *Cell* **177**, 837-851 e828 (2019). https://doi.org/10.1016/j.cell.2019.02.050

99 Monot, C. *et al.* The specificity and flexibility of l1 reverse transcription priming at imperfect T-tracts. *PLoS Genet* **9**, e1003499 (2013). https://doi.org/10.1371/journal.pgen.1003499

100 Burns, K. H. Our Conflict with Transposable Elements and Its Implications for Human Disease. *Annu Rev Pathol* **15**, 51-70 (2020). https://doi.org/10.1146/annurev-pathmechdis-012419-032633

101 Smit, A. F., Hubley, R. & Green, P. *RepeatMasker Open–4.0.*, <http://www.repeatmasker.org> (2015).

102 Kumar, S. *et al.* TimeTree 5: An Expanded Resource for Species Divergence Times. *Mol Biol Evol* **39** (2022). https://doi.org/10.1093/molbev/msac174
103 Wylie, A. *et al.* p53 genes function to restrain mobile elements. *Genes Dev* **30**, 64-77 (2016). https://doi.org/10.1101/gad.266098.115
104 Ardeljan, D. *et al.* Cell fitness screens reveal a conflict between LINE-1 retrotransposition and DNA replication. *Nat Struct Mol Biol* **27**, 168-178 (2020). https://doi.org/10.1038/s41594-020-0372-1
105 Liu, N. *et al.* Selective silencing of euchromatic L1s revealed by genome-wide screens for L1 regulators. *Nature* **553**, 228-232 (2018). https://doi.org/10.1038/nature25179
106 Mita, P. *et al.* BRCA1 and S phase DNA repair pathways restrict LINE-1 retrotransposition in human cells. *Nat Struct Mol Biol* **27**, 179-191 (2020). https://doi.org/10.1038/s41594-020-0374-z
107 Luqman-Fatah, A. *et al.* The interferon stimulated gene-encoded protein HELZ2 inhibits human LINE-1 retrotransposition and LINE-1 RNA-mediated type I interferon induction. *Nat Commun* **14**, 203 (2023). https://doi.org/10.1038/s41467-022-35757-6
108 Miyoshi, T., Makino, T. & Moran, J. V. Poly(ADP-Ribose) Polymerase 2 Recruits Replication Protein A to Sites of LINE-1 Integration to Facilitate Retrotransposition. *Mol Cell* **75**, 1286-1298 e1212 (2019). https://doi.org/10.1016/j.molcel.2019.07.018
109 Gasior, S. L., Wakeman, T. P., Xu, B. & Deininger, P. L. The human LINE-1 retrotransposon creates DNA double-strand breaks. *J Mol Biol* **357**, 1383-1393 (2006). https://doi.org/10.1016/j.jmb.2006.01.089
110 Belgnaoui, S. M., Gosden, R. G., Semmes, O. J. & Haoudi, A. Human LINE-1 retrotransposon induces DNA damage and apoptosis in cancer cells. *Cancer Cell Int* **6**, 13 (2006). https://doi.org/10.1186/1475-2867-6-13
111 Wallace, N. A., Belancio, V. P. & Deininger, P. L. L1 mobile element expression causes multiple types of toxicity. *Gene* **419**, 75-81 (2008). https://doi.org/10.1016/j.gene.2008.04.013
112 McKerrow, W. *et al.* LINE-1 expression in cancer correlates with p53 mutation, copy number alteration, and S phase checkpoint. *Proc Natl Acad Sci U S A* **119** (2022). https://doi.org/10.1073/pnas.2115999119
113 De Cecco, M. *et al.* L1 drives IFN in senescent cells and promotes age-associated inflammation. *Nature* **566**, 73-78 (2019). https://doi.org/10.1038/s41586-018-0784-9
114 Simon, M. *et al.* LINE1 Derepression in Aged Wild-Type and SIRT6-Deficient Mice Drives Inflammation. *Cell Metab* **29**, 871-885 e875 (2019). https://doi.org/10.1016/j.cmet.2019.02.014
115 Gorbunova, V. *et al.* The role of retrotransposable elements in ageing and age-associated diseases. *Nature* **596**, 43-53 (2021). https://doi.org/10.1038/s41586-021-03542-y
116 Šulc, P. *et al.* Repeats Mimic Pathogen-Associated Patterns Across a Vast Evolutionary Landscape. *bioRxiv*, 2021.2011.2004.467016 (2023). https://doi.org/10.1101/2021.11.04.467016
117 Peze-Heidsieck, E. *et al.* Retrotransposons as a Source of DNA Damage in Neurodegeneration. *Front Aging Neurosci* **13**, 786897 (2021). https://doi.org/10.3389/fnagi.2021.786897
118 Terry, D. M. & Devine, S. E. Aberrantly High Levels of Somatic LINE-1 Expression and Retrotransposition in Human Neurological Disorders. *Front Genet* **10**, 1244 (2019). https://doi.org/10.3389/fgene.2019.01244
119 Zhang, X., Zhang, R. & Yu, J. New Understanding of the Relevant Role of LINE-1 Retrotransposition in Human Disease and Immune Modulation. *Front Cell Dev Biol* **8**, 657 (2020). https://doi.org/10.3389/fcell.2020.00657
120 Fukuda, S. *et al.* Alu complementary DNA is enriched in atrophic macular degeneration and triggers retinal pigmented epithelium toxicity via cytosolic innate immunity. *Sci Adv* **7**, eabj3658 (2021). https://doi.org/10.1126/sciadv.abj3658
121 Roulois, D. *et al.* DNA-Demethylating Agents Target Colorectal Cancer Cells by Inducing Viral Mimicry by Endogenous Transcripts. *Cell* **162**, 961-973 (2015). https://doi.org/10.1016/j.cell.2015.07.056
122 Sun, S. *et al.* Cancer cells co-evolve with retrotransposons to mitigate viral mimicry. *bioRxiv* (2023). https://doi.org/10.1101/2023.05.19.541456
123 Solovyov, A. *et al.* Mechanism-guided quantification of LINE-1 reveals p53 regulation of both retrotransposition and transcription. *bioRxiv*, 2023.2005.2011.539471 (2023). https://doi.org/10.1101/2023.05.11.539471
124 Ishizuka, J. J. *et al.* Loss of ADAR1 in tumours overcomes resistance to immune checkpoint blockade. *Nature* **565**, 43-48 (2019). https://doi.org/10.1038/s41586-018-0768-9
125 Chiappinelli, K. B. *et al.* Inhibiting DNA Methylation Causes an Interferon Response in Cancer via dsRNA Including Endogenous Retroviruses. *Cell* **162**, 974-986 (2015). https://doi.org/10.1016/j.cell.2015.07.011

126 Leonova, K. I. *et al.* p53 cooperates with DNA methylation and a suicidal interferon response to maintain epigenetic silencing of repeats and noncoding RNAs. *Proc Natl Acad Sci U S A* **110**, E89-98 (2013). https://doi.org/10.1073/pnas.1216922110
127 Novototskaya-Vlasova, K. A. *et al.* Inflammatory response to retrotransposons drives tumor drug resistance that can be prevented by reverse transcriptase inhibitors. *Proc Natl Acad Sci U S A* **119**, e2213146119 (2022). https://doi.org/10.1073/pnas.2213146119
128 Takahashi, T. *et al.* LINE-1 activation in the cerebellum drives ataxia. *Neuron* **110**, 3278-3287 e3278 (2022). https://doi.org/10.1016/j.neuron.2022.08.011
129 Jones, R. B. *et al.* Nucleoside analogue reverse transcriptase inhibitors differentially inhibit human LINE-1 retrotransposition. *PLoS One* **3**, e1547 (2008). https://doi.org/10.1371/journal.pone.0001547
130 Xie, Y. *et al.* Cell division promotes efficient retrotransposition in a stable L1 reporter cell line. *Mob DNA* **4**, 10 (2013). https://doi.org/10.1186/1759-8753-4-10
131 Banuelos-Sanchez, G. *et al.* Synthesis and Characterization of Specific Reverse Transcriptase Inhibitors for Mammalian LINE-1 Retrotransposons. *Cell Chem Biol* **26**, 1095-1109 e1014 (2019). https://doi.org/10.1016/j.chembiol.2019.04.010
132 Dai, L., Huang, Q. & Boeke, J. D. Effect of reverse transcriptase inhibitors on LINE-1 and Ty1 reverse transcriptase activities and on LINE-1 retrotransposition. *BMC Biochem* **12**, 18 (2011). https://doi.org/10.1186/1471-2091-12-18
133 Xie, Y., Rosser, J. M., Thompson, T. L., Boeke, J. D. & An, W. Characterization of L1 retrotransposition with high-throughput dual-luciferase assays. *Nucleic Acids Res* **39**, e16 (2011). https://doi.org/10.1093/nar/gkq1076
134 Hsiou, Y. *et al.* Structure of unliganded HIV-1 reverse transcriptase at 2.7 A resolution: implications of conformational changes for polymerization and inhibition mechanisms. *Structure* **4**, 853-860 (1996). https://doi.org/10.1016/s0969-2126(96)00091-3
135 Ruiz, F. X., Hoang, A., Dilmore, C. R., DeStefano, J. J. & Arnold, E. Structural basis of HIV inhibition by L-nucleosides: Opportunities for drug development and repurposing. *Drug Discov Today* **27**, 1832-1846 (2022). https://doi.org/10.1016/j.drudis.2022.02.016
136 Ren, J. *et al.* Structural mechanisms of drug resistance for mutations at codons 181 and 188 in HIV-1 reverse transcriptase and the improved resilience of second generation non-nucleoside inhibitors. *J Mol Biol* **312**, 795-805 (2001). https://doi.org/10.1006/jmbi.2001.4988
137 Ruiz, F. & Arnold, E. Evolving understanding of HIV-1 reverse transcriptase structure, function, inhibition, and resistance. *Curr Opin Struct Biol* **61**, 113-123 (2020). https://doi.org/10.1016/j.sbi.2019.11.011
138 Golinelli, M. P. & Hughes, S. H. Nontemplated nucleotide addition by HIV-1 reverse transcriptase. *Biochemistry* **41**, 5894-5906 (2002). https://doi.org/10.1021/bi0160415
139 Wulf, M. G. *et al.* Chemical capping improves template switching and enhances sequencing of small RNAs. *Nucleic Acids Res* **50**, e2 (2022). https://doi.org/10.1093/nar/gkab861
140 Lentzsch, A. M., Stamos, J. L., Yao, J., Russell, R. & Lambowitz, A. M. Structural basis for template switching by a group II intron-encoded non-LTR-retroelement reverse transcriptase. *J Biol Chem* **297**, 100971 (2021). https://doi.org/10.1016/j.jbc.2021.100971
141 Lentzsch, A. M., Yao, J., Russell, R. & Lambowitz, A. M. Template-switching mechanism of a group II intron-encoded reverse transcriptase and its implications for biological function and RNA-Seq. *J Biol Chem* **294**, 19764-19784 (2019). https://doi.org/10.1074/jbc.RA119.011337
142 Bibillo, A. & Eickbush, T. H. End-to-end template jumping by the reverse transcriptase encoded by the R2 retrotransposon. *J Biol Chem* **279**, 14945-14953 (2004). https://doi.org/10.1074/jbc.M310450200
143 Jamburuthugoda, V. K. & Eickbush, T. H. Identification of RNA binding motifs in the R2 retrotransposon-encoded reverse transcriptase. *Nucleic Acids Res* **42**, 8405-8415 (2014). https://doi.org/10.1093/nar/gku514
144 Benitez-Guijarro, M. *et al.* RNase H2, mutated in Aicardi-Goutieres syndrome, promotes LINE-1 retrotransposition. *EMBO J* **37** (2018). https://doi.org/10.15252/embj.201798506
145 Rice, G. I. *et al.* Reverse-Transcriptase Inhibitors in the Aicardi-Goutieres Syndrome. *N Engl J Med* **379**, 2275-2277 (2018). https://doi.org/10.1056/NEJMc1810983
146 Holm, L. Dali server: structural unification of protein families. *Nucleic Acids Res* **50**, W210-W215 (2022). https://doi.org/10.1093/nar/gkac387

147 van Kempen, M. *et al.* Fast and accurate protein structure search with Foldseek. *Nature Biotechnology* (2023). https://doi.org/10.1038/s41587-023-01773-0
148 An, W. *et al.* Characterization of a synthetic human LINE-1 retrotransposon ORFeus-Hs. *Mob DNA* **2**, 2 (2011). https://doi.org/10.1186/1759-8753-2-2
149 Rogala, K. B. *et al.* Structural basis for the docking of mTORC1 on the lysosomal surface. *Science* **366**, 468-475 (2019). https://doi.org/10.1126/science.aay0166
150 Sari, D. *et al.* The MultiBac Baculovirus/Insect Cell Expression Vector System for Producing Complex Protein Biologics. *Adv Exp Med Biol* **896**, 199-215 (2016). https://doi.org/10.1007/978-3-319-27216-0_13
151 Baldwin, E. T. *et al.* Human endogenous retrovirus-K (HERV-K) reverse transcriptase (RT) structure and biochemistry reveals remarkable similarities to HIV-1 RT and opportunities for HERV-K-specific inhibition. *Proc Natl Acad Sci U S A* **119**, e2200260119 (2022). https://doi.org/10.1073/pnas.2200260119
152 Jumper, J. *et al.* Highly accurate protein structure prediction with AlphaFold. *Nature* **596**, 583-589 (2021). https://doi.org/10.1038/s41586-021-03819-2
153 Casanal, A., Lohkamp, B. & Emsley, P. Current developments in Coot for macromolecular model building of Electron Cryo-microscopy and Crystallographic Data. *Protein Sci* **29**, 1069-1078 (2020). https://doi.org/10.1002/pro.3791
154 Blanc, E. *et al.* Refinement of severely incomplete structures with maximum likelihood in BUSTER-TNT. *Acta Crystallogr D Biol Crystallogr* **60**, 2210-2221 (2004). https://doi.org/10.1107/S0907444904016427
155 Adasme, M. F. *et al.* PLIP 2021: expanding the scope of the protein–ligand interaction profiler to DNA and RNA. *Nucleic Acids Research* **49**, W530-W534 (2021). https://doi.org/10.1093/nar/gkab294
156 Adasme, M. F. *et al.* PLIP 2021: expanding the scope of the protein-ligand interaction profiler to DNA and RNA. *Nucleic Acids Res* **49**, W530-W534 (2021). https://doi.org/10.1093/nar/gkab294
157 Zhang, J. H., Chen, T., Nguyen, S. H. & Oldenburg, K. R. A high-throughput homogeneous assay for reverse transcriptase using generic reagents and time-resolved fluorescence detection. *Anal Biochem* **281**, 182-186 (2000). https://doi.org/10.1006/abio.2000.4567
158 Fenaux, M., Saunders, O., Yokokawa, F. & Zhong, W. Alkynyl nucleoside analogs as inhibitors of human rhinovirus. USA patent 9,988,416 (2018).
159 Lama, L. *et al.* Development of human cGAS-specific small-molecule inhibitors for repression of dsDNA-triggered interferon expression. *Nat Commun* **10**, 2261 (2019). https://doi.org/10.1038/s41467-019-08620-4
160 Crossley, M. P. *et al.* Catalytically inactive, purified RNase H1: A specific and sensitive probe for RNA-DNA hybrid imaging. *J Cell Biol* **220** (2021). https://doi.org/10.1083/jcb.202101092
161 Mates, L. *et al.* Molecular evolution of a novel hyperactive Sleeping Beauty transposase enables robust stable gene transfer in vertebrates. *Nat Genet* **41**, 753-761 (2009). https://doi.org/10.1038/ng.343
162 Moran, J. V. *et al.* High frequency retrotransposition in cultured mammalian cells. *Cell* **87**, 917-927 (1996). https://doi.org/10.1016/s0092-8674(00)81998-4
163 Wu, T. *et al.* Three Essential Resources to Improve Differential Scanning Fluorimetry (DSF) Experiments. *bioRxiv*, 2020.2003.2022.002543 (2020). https://doi.org/10.1101/2020.03.22.002543
164 Chen, Z. L. *et al.* A high-speed search engine pLink 2 with systematic evaluation for proteome-scale identification of cross-linked peptides. *Nat Commun* **10**, 3404 (2019). https://doi.org/10.1038/s41467-019-11337-z
165 Yilmaz, S., Busch, F., Nagaraj, N. & Cox, J. Accurate and Automated High-Coverage Identification of Chemically Cross-Linked Peptides with MaxLynx. *Anal Chem* **94**, 1608-1617 (2022). https://doi.org/10.1021/acs.analchem.1c03688
166 Klykov, O. *et al.* Efficient and robust proteome-wide approaches for cross-linking mass spectrometry. *Nat Protoc* **13**, 2964-2990 (2018). https://doi.org/10.1038/s41596-018-0074-x
167 Mastronarde, D. N. SerialEM: A Program for Automated Tilt Series Acquisition on Tecnai Microscopes Using Prediction of Specimen Position. *Microscopy and Microanalysis* **9**, 1182-1183 (2003). https://doi.org/10.1017/s1431927603445911
168 Naydenova, K. & Russo, C. J. Measuring the effects of particle orientation to improve the efficiency of electron cryomicroscopy. *Nat Commun* **8**, 629 (2017). https://doi.org/10.1038/s41467-017-00782-3
169 Suloway, C. *et al.* Automated molecular microscopy: the new Leginon system. *J Struct Biol* **151**, 41-60 (2005). https://doi.org/10.1016/j.jsb.2005.03.010

170 Zheng, S. Q. *et al.* MotionCor2: anisotropic correction of beam-induced motion for improved cryo-electron microscopy. *Nat Methods* **14**, 331-332 (2017). https://doi.org/10.1038/nmeth.4193
171 Punjani, A., Rubinstein, J. L., Fleet, D. J. & Brubaker, M. A. cryoSPARC: algorithms for rapid unsupervised cryo-EM structure determination. *Nat Methods* **14**, 290-296 (2017). https://doi.org/10.1038/nmeth.4169
172 Punjani, A., Zhang, H. & Fleet, D. J. Non-uniform refinement: adaptive regularization improves single-particle cryo-EM reconstruction. *Nat Methods* **17**, 1214-1221 (2020). https://doi.org/10.1038/s41592-020-00990-8
173 Zivanov, J. *et al.* New tools for automated high-resolution cryo-EM structure determination in RELION-3. *Elife* **7** (2018). https://doi.org/10.7554/eLife.42166
174 Asarnow, D., Palovcak, E. & Cheng, Y. *UCSF pyem*.
175 Rohou, A. & Grigorieff, N. CTFFIND4: Fast and accurate defocus estimation from electron micrographs. *J Struct Biol* **192**, 216-221 (2015). https://doi.org/10.1016/j.jsb.2015.08.008
176 Sanchez-Garcia, R. *et al.* DeepEMhancer: a deep learning solution for cryo-EM volume post-processing. *Commun Biol* **4**, 874 (2021). https://doi.org/10.1038/s42003-021-02399-1
177 Afonine, P. V. *et al.* Real-space refinement in PHENIX for cryo-EM and crystallography. *Acta Crystallogr D Struct Biol* **74**, 531-544 (2018). https://doi.org/10.1107/S2059798318006551
178 Wagner, T. *et al.* SPHIRE-crYOLO is a fast and accurate fully automated particle picker for cryo-EM. *Commun Biol* **2**, 218 (2019). https://doi.org/10.1038/s42003-019-0437-z
179 Bell, J. M., Chen, M., Baldwin, P. R. & Ludtke, S. J. High resolution single particle refinement in EMAN2.1. *Methods* **100**, 25-34 (2016). https://doi.org/10.1016/j.ymeth.2016.02.018
180 Alber, F. *et al.* Determining the architectures of macromolecular assemblies. *Nature* **450**, 683-694 (2007). https://doi.org/10.1038/nature06404
181 Sali, A. From integrative structural biology to cell biology. *J Biol Chem* **296**, 100743 (2021). https://doi.org/10.1016/j.jbc.2021.100743
182 Rout, M. P. & Sali, A. Principles for Integrative Structural Biology Studies. *Cell* **177**, 1384-1403 (2019). https://doi.org/10.1016/j.cell.2019.05.016
183 Russel, D. *et al.* Putting the pieces together: integrative modeling platform software for structure determination of macromolecular assemblies. *PLoS Biol* **10**, e1001244 (2012). https://doi.org/10.1371/journal.pbio.1001244
184 Rotkiewicz, P. & Skolnick, J. Fast procedure for reconstruction of full-atom protein models from reduced representations. *J Comput Chem* **29**, 1460-1465 (2008). https://doi.org/10.1002/jcc.20906
185 Krivov, G. G., Shapovalov, M. V. & Dunbrack, R. L., Jr. Improved prediction of protein side-chain conformations with SCWRL4. *Proteins* **77**, 778-795 (2009). https://doi.org/10.1002/prot.22488
186 Liebschner, D. *et al.* Macromolecular structure determination using X-rays, neutrons and electrons: recent developments in Phenix. *Acta Crystallogr D Struct Biol* **75**, 861-877 (2019). https://doi.org/10.1107/S2059798319011471
187 Abraham, M. J. *et al.* GROMACS: High performance molecular simulations through multi-level parallelism from laptops to supercomputers. *SoftwareX* **1-2**, 19-25 (2015). https://doi.org/https://doi.org/10.1016/j.softx.2015.06.001
188 Michaud-Agrawal, N., Denning, E. J., Woolf, T. B. & Beckstein, O. MDAnalysis: a toolkit for the analysis of molecular dynamics simulations. *J Comput Chem* **32**, 2319-2327 (2011). https://doi.org/10.1002/jcc.21787
189 Zhang, S. *et al.* ProDy 2.0: increased scale and scope after 10 years of protein dynamics modelling with Python. *Bioinformatics* **37**, 3657-3659 (2021). https://doi.org/10.1093/bioinformatics/btab187
190 Das, K. *et al.* Conformational States of HIV-1 Reverse Transcriptase for Nucleotide Incorporation vs Pyrophosphorolysis-Binding of Foscarnet. *ACS Chem Biol* **11**, 2158-2164 (2016). https://doi.org/10.1021/acschembio.6b00187
191 Collier, J. H. *et al.* Statistical inference of protein structural alignments using information and compression. *Bioinformatics* **33**, 1005-1013 (2017). https://doi.org/10.1093/bioinformatics/btw757
192 Pettersen, E. F. *et al.* UCSF ChimeraX: Structure visualization for researchers, educators, and developers. *Protein Sci* **30**, 70-82 (2021). https://doi.org/10.1002/pro.3943
193 Cost, G. J., Feng, Q., Jacquier, A. & Boeke, J. D. Human L1 element target-primed reverse transcription in vitro. *EMBO J* **21**, 5899-5910 (2002). https://doi.org/10.1093/emboj/cdf592

194 Pimentel, S. C., Upton, H. E. & Collins, K. Separable structural requirements for cDNA synthesis, nontemplated extension, and template jumping by a non-LTR retroelement reverse transcriptase. *J Biol Chem* **298**, 101624 (2022). https://doi.org/10.1016/j.jbc.2022.101624
195 Kabinger, F. *et al.* Mechanism of molnupiravir-induced SARS-CoV-2 mutagenesis. *Nat Struct Mol Biol* **28**, 740-746 (2021). https://doi.org/10.1038/s41594-021-00651-0
196 Stamos, J. L., Lentzsch, A. M. & Lambowitz, A. M. Structure of a Thermostable Group II Intron Reverse Transcriptase with Template-Primer and Its Functional and Evolutionary Implications. *Mol Cell* **68**, 926-939 e924 (2017). https://doi.org/10.1016/j.molcel.2017.10.024
197 Das, K., Martinez, S. E., Bandwar, R. P. & Arnold, E. Structures of HIV-1 RT-RNA/DNA ternary complexes with dATP and nevirapine reveal conformational flexibility of RNA/DNA: insights into requirements for RNase H cleavage. *Nucleic Acids Res* **42**, 8125-8137 (2014). https://doi.org/10.1093/nar/gku487
