# Supplementary material for: Structures, functions and adaptations of the human LINE-1 ORF2 protein
Source: Nature. 2023 Dec 14;626(7997):194–206. doi: 10.1038/s41586-023-06947-z (PMC10830420; doi:10.1038/s41586-023-06947-z)
Supplement: Supplementary file 2 — Reporting Summary [file 41586_2023_6947_MOESM2_ESM.pdf]

Corresponding author(s): Martin Taylor, John LaCava, Donna Romero, Benjamin Greenbaum, Eddy Arnold, Michael Rout, Matthias Götte, Kathleen Burns

Last updated by author(s): Nov/22/23

Reporting Summary

Nature Portfolio wishes to improve the reproducibility of the work that we publish. This form provides structure for consistency and transparency in reporting. For further information on Nature Portfolio policies, see our Editorial Policies and the Editorial Policy Checklist.

Statistics

For all statistical analyses, confirm that the following items are present in the figure legend, table legend, main text, or Methods section.

n/a	Confirmed	
☐	☒	The exact sample size (n) for each experimental group/condition, given as a discrete number and unit of measurement
☐	☒	A statement on whether measurements were taken from distinct samples or whether the same sample was measured repeatedly
☐	☒	The statistical test(s) used AND whether they are one- or two-sided<br>*Only common tests should be described solely by name; describe more complex techniques in the Methods section.*
☒	☐	A description of all covariates tested
☐	☒	A description of any assumptions or corrections, such as tests of normality and adjustment for multiple comparisons
☐	☒	A full description of the statistical parameters including central tendency (e.g. means) or other basic estimates (e.g. regression coefficient) AND variation (e.g. standard deviation) or associated estimates of uncertainty (e.g. confidence intervals)
☐	☒	For null hypothesis testing, the test statistic (e.g. F, t, r) with confidence intervals, effect sizes, degrees of freedom and P value noted<br>*Give P values as exact values whenever suitable.*
☐	☒	For Bayesian analysis, information on the choice of priors and Markov chain Monte Carlo settings
☐	☒	For hierarchical and complex designs, identification of the appropriate level for tests and full reporting of outcomes
☐	☒	Estimates of effect sizes (e.g. Cohen's d, Pearson's r), indicating how they were calculated

Our web collection on statistics for biologists contains articles on many of the points above.

Software and code

Policy information about availability of computer code

Data collection	Full descriptions including versions are provided in the methods. For mass spectrometry, RAW data was searched using pLink 2.3.9, MaxLynx (MaxQuant 2.1.4.0) and Proteome Discoverer 2.4 with the XlinkX plugin. For crystallography, DIALS 3.14, Aimless 0.7.7, Phaser 3.60.1, Coot 0.9.6, and Buster 2.10.4. For cryoEM, MotionCor2, cryoSPARC v.3.1.0, Relion 3.1, CTFFIND 4.1, cryoEF 1.1.0, SerialEM 4.0, DeepEMhancer 0.14. For integrative modeling and molecular dynamics, GROMACS 2023, MDanalysis v2.4.3, ProDy v2.4, Integrative Modeling Platform (IMP) package 2.18. For ORF2p-ligand modeling and FEP+ Schrödinger Suite version 2023-1. For evolutionary analysis, Clustal Omega version 1.2.4, MUSTANG version 3.2.4, MMLigner version 1.0.2, Python scikit-learn 1.2.2.
Data analysis	Data were plotted using combinations of Matplotlib v3.7.0, Seaborn, and pyCircos v0.3.0 packages and Prism 9.5 (GraphPad). Structures were visualized with ChimeraX v1.5131. Full reports from PDB of the crystal and EM data are provided in a separate file.

For manuscripts utilizing custom algorithms or software that are central to the research but not yet described in published literature, software must be made available to editors and reviewers. We strongly encourage code deposition in a community repository (e.g. GitHub). See the Nature Portfolio guidelines for submitting code & software for further information.

Data

Policy information about availability of data

All manuscripts must include a data availability statement. This statement should provide the following information, where applicable:

- Accession codes, unique identifiers, or web links for publicly available datasets
- A description of any restrictions on data availability
- For clinical datasets or third party data, please ensure that the statement adheres to our policy

The coordinates for the ORF2p crystal structure have been deposited in the PDB ID: 8C8J. The single particle cryo-EM maps for ORF2p core have been deposited in the EMDB and their associated model coordinates in the PDB under the accession numbers: EMD-40858, PDB ID: 8SXT (heteroduplex); EMD-40859,8SXU (oligo(A)); EMD-40856(apo). Raw movies and motion corrected micrographs for apo ORF2p has been deposited in the Electron Microscopy Public Image Archive under the accession number EMPIAR-11556. The mass spectrometry proteomics data have been deposited to the ProteomeXchange Consortium (http://proteomecentral.proteomexchange.org) via the PRIDE partner repository with the dataset identifier PXD038615. Files containing the input data, scripts, and output results are available at https://integrativemodeling/ORF2p and the nascent integrative modeling section of the worldwide Protein Data Bank (wwPDB) PDB-Dev95 repository for integrative structures and corresponding data under accession code PDBDEV_00000211. AlphaFold2 predictions, Molecular dynamics simulations results, and full-atom versions of best-matching models are available in ModelArchive repository [https://www.modelarchive.org/doi/10.5452/ma-fejd6, https://www.modelarchive.org/doi/10.5452/ma-joo4d, https://www.modelarchive.org/doi/10.5452/ma-lzyrq https://www.modelarchive.org/doi/10.5452/ma-xlzzy, https://www.modelarchive.org/doi/10.5452/ma-9wovj]

Research involving human participants, their data, or biological material

Policy information about studies with human participants or human data. See also policy information about sex, gender (identity/presentation), and sexual orientation and race, ethnicity and racism.

Reporting on sex and gender	N/a
Reporting on race, ethnicity, or other socially relevant groupings	N/a
Population characteristics	N/a
Recruitment	N/a
Ethics oversight	N/a

Note that full information on the approval of the study protocol must also be provided in the manuscript.

Field-specific reporting

Please select the one below that is the best fit for your research. If you are not sure, read the appropriate sections before making your selection.

☒ Life sciences ☐ Behavioural & social sciences ☐ Ecological, evolutionary & environmental sciences

For a reference copy of the document with all sections, see nature.com/documents/nr-reporting-summary-flat.pdf

Life sciences study design

All studies must disclose on these points even when the disclosure is negative.

Sample size	Sample size is explicitly stated in figure legends where possible and in the Statistics and Reproducibility section in the Methods. Crystallography data and numbers of crystals reported in methods per experiment. Particle numbers for cryo-EM are reported in each experiment and in relevant tables and workflow figures. For biochemistry n=2 or n=3 reactions were setup in parallel and the experiments were repeated at least two times. For cell-based assays, n=3 or larger experiments were setup in parallel and the experiments were repeated at least three times.
Data exclusions	No data were excluded
Replication	Where replicates were appropriate, n>=3 was used, such as biochemical measurements.
Randomization	n/a
Blinding	n/a

Reporting for specific materials, systems and methods

We require information from authors about some types of materials, experimental systems and methods used in many studies. Here, indicate whether each material, system or method listed is relevant to your study. If you are not sure if a list item applies to your research, read the appropriate section before selecting a response.

Materials & experimental systems

n/a	Involved in the study
☐	☒ Antibodies
☐	☒ Eukaryotic cell lines
☒	☐ Palaeontology and archaeology
☒	☐ Animals and other organisms
☒	☐ Clinical data
☒	☐ Dual use research of concern
☒	☐ Plants

Methods

n/a	Involved in the study
☒	☐ ChIP-seq
☒	☐ Flow cytometry
☒	☐ MRI-based neuroimaging

Antibodies

Antibodies used

Rabbit monoclonal S9.6 (Kerafast Kf-Ab01137-23.0 lot T2216B05), mouse anti-Flag M2 (Sigma #F1804), mouse anti-ORF1 4H1 (Burns lab stock; available as Millipore MABC1152), GFP-tag polyclonal (Life Technologies # 50430-2-AP lot 00110230). Catalytically inactive D210N human RNase H1 (dRNH1) affinity / imaging reagent, while not an antibody, was purified from E. Coli expressing Addgene Plasmid #174448, a gift from Dr. Karlene Cimprich (Methods).

Validation

S9.6 was generated in the Leppla lab in 2006 and has been validated in at least 94 publications. Hybrid signal in our work from S9.6 was also validated by its absence in an ORF2p RT mutant and in with RT inhibitor treatment. anti-Flag M2 (Sigma #F1804 lot 035K6196) is extensively validated; ORF2p-Flag staining was further validated by co-localization with ORF1p. Mouse anti-ORF1 4H1 (Burns lab stock; available as Millipore MABC1152) was used from aliquots frozen from original stocks described and validated in Taylor et al. Cell 2013 (doi: 10.1016/j.cell.2013.10.021) stored at -80C, recently re-validated in Taylor et al. Cancer Discovery 2023 Supplementary Figure 15 (doi: 10.1158/2159-8290.CD-23-0313), and by co-localization with ORF2p and L1 granules. GFP-tag polyclonal (Life Technologies # 50430-2-AP) is verified by the manufacturer to bind specifically to the tag in imaging and blotting applications and has been cited in 1783 papers and was further validated in controls lacking dRNH1. dRNH1 was validated in Crossley et. al JCB 2021 (doi: 10.1083/jcb.202101092) and we re-validated the plasmid by whole plasmid sequencing; the purified protein was validated by GFP fluorescence, heparin binding, and molecular weight of the fusion protein on Coomassie-stained SDS-PAGE gels. Imaging results with dRNH1 were further validated by the absence of cytoplasmic signal in untransfected cells, RT- LINE-1 transfections, and with RT inhibitor treatment. All antibodies used in imaging were further validated by specific signal present only in the transfected subset of cells and co-localization of specific signals.

Eukaryotic cell lines

Policy information about cell lines and Sex and Gender in Research

Cell line source(s)

HeLa Tet-On 3G cell line was from Takara; MCF7, HeLa and U2-OS from American Type Culture Collection (ATCC); THP1-Dual and THP1-Dual KO-TREX1 cells were from InvivoGen.

Authentication

THP1 cells were authenticated by resistance to blasticidin and Zeocin. TREX1 presence or knockout was authenticated by western blotting and by interferon production after decitabine treatment. HeLa Tet-On were validated by doxycycline-inducible production of ORF1p after stable integration of a tet-on LINE-1 expressing plasmid. HeLa, U2-OS, and MCF7 cells were not authenticated after receipt from ATCC.

Mycoplasma contamination

Cell lines were tested monthly and were negative for mycoplasma.

Commonly misidentified lines
(See ICLAC register)

n/a
