# Supplementary material for: Structures, functions and adaptations of the human LINE-1 ORF2 protein
Source: Nature. 2023 Dec 14;626(7997):194–206. doi: 10.1038/s41586-023-06947-z (PMC10830420; doi:10.1038/s41586-023-06947-z)

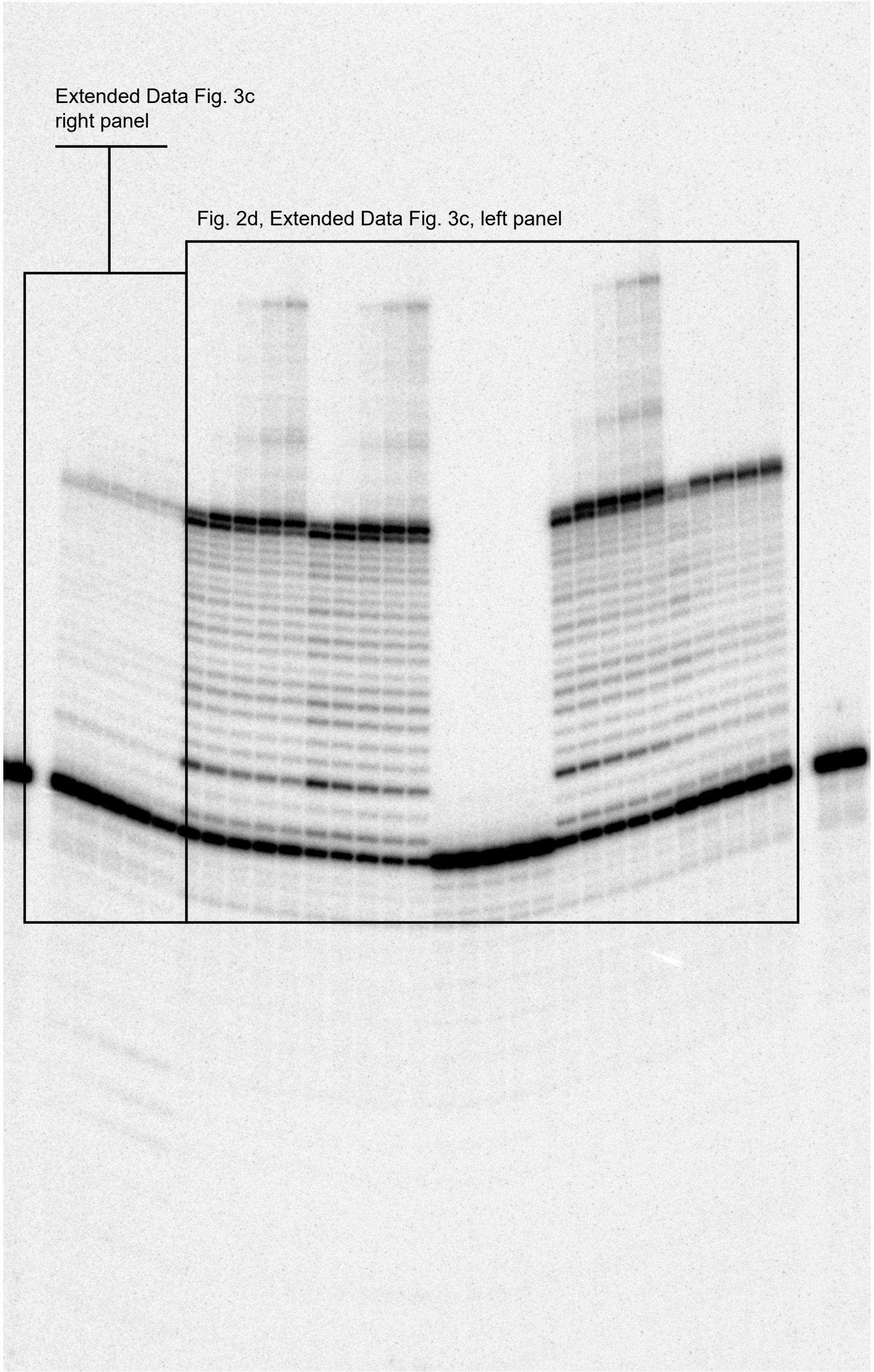

Extended Data Fig. 3d

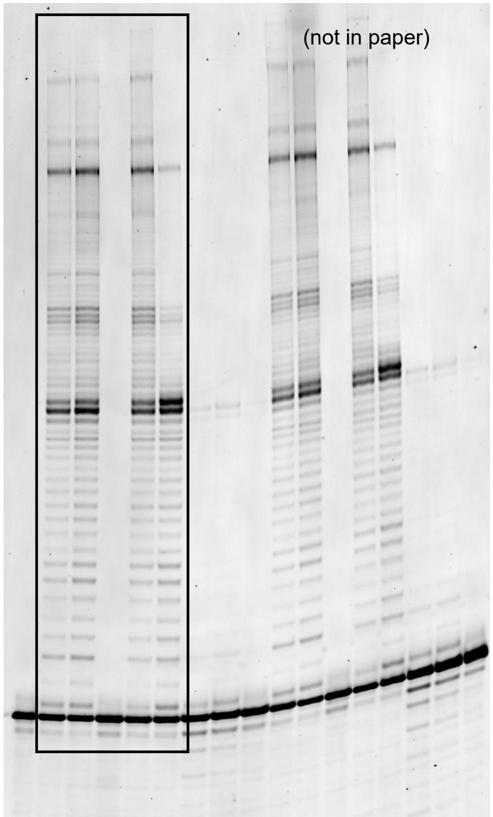

Fig. 3a, Extended Data Fig. 3c, Supplemental Fig. 3b

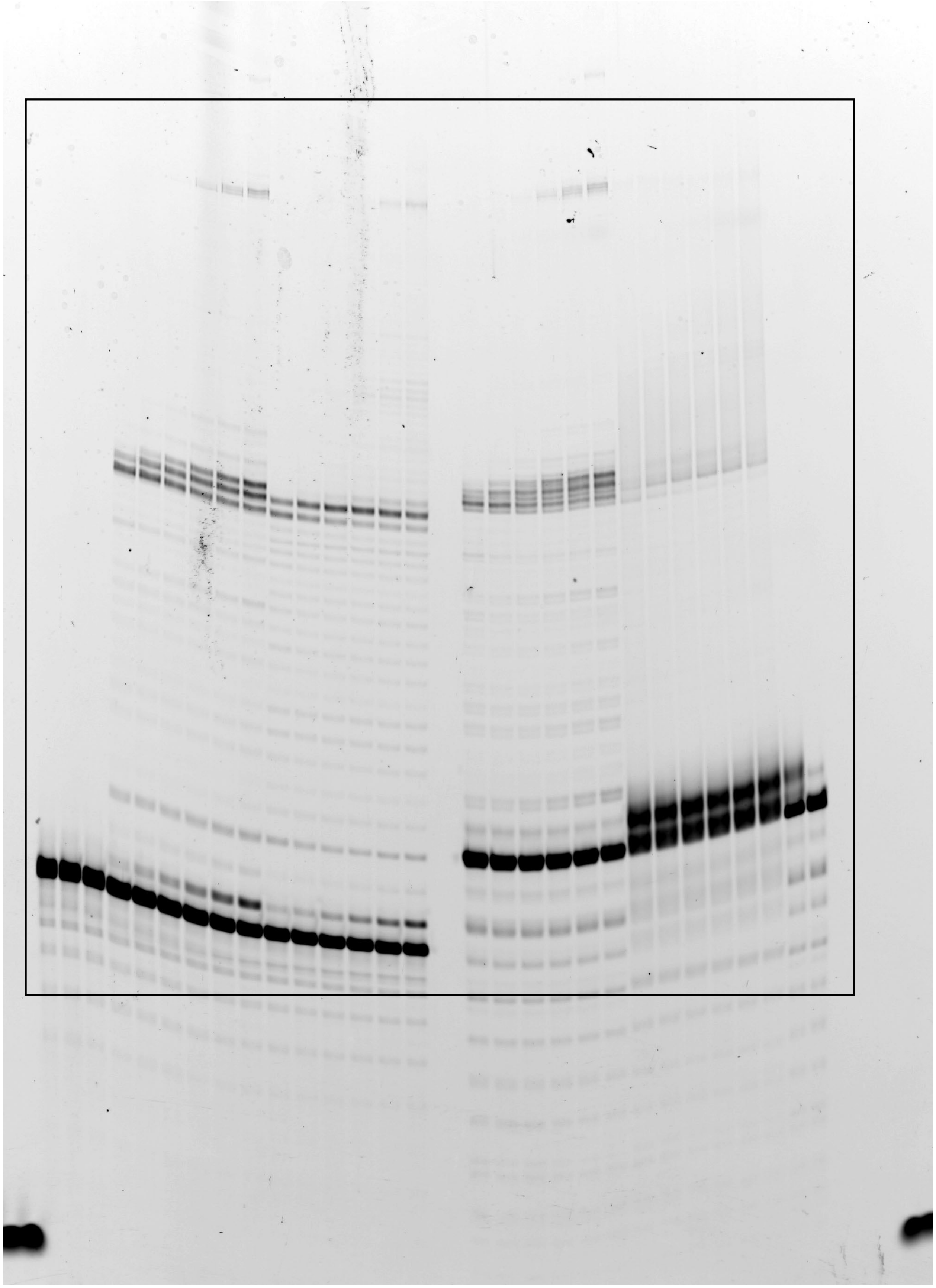

Supplemental Fig. 3a

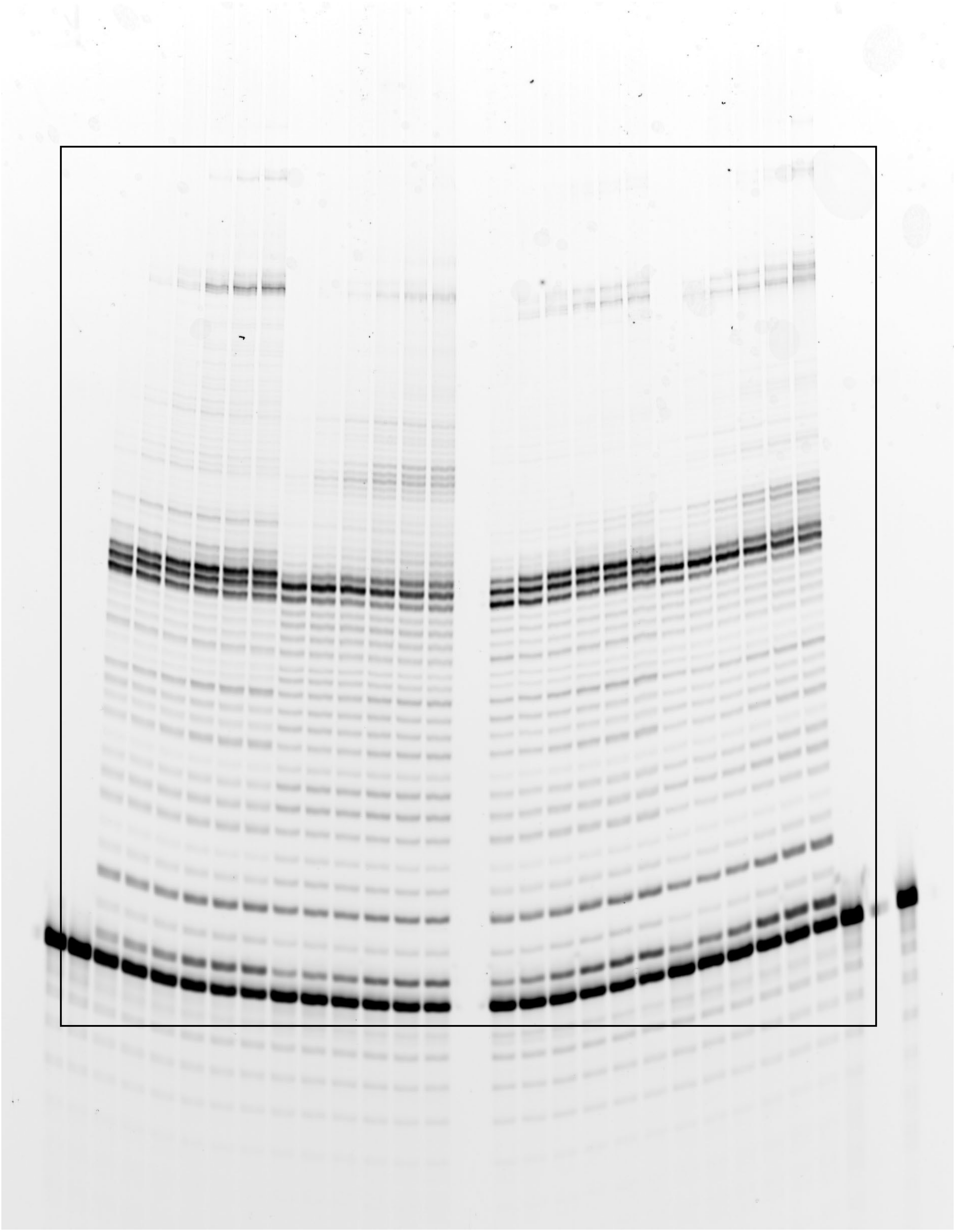

Supplemental Fig. 3c

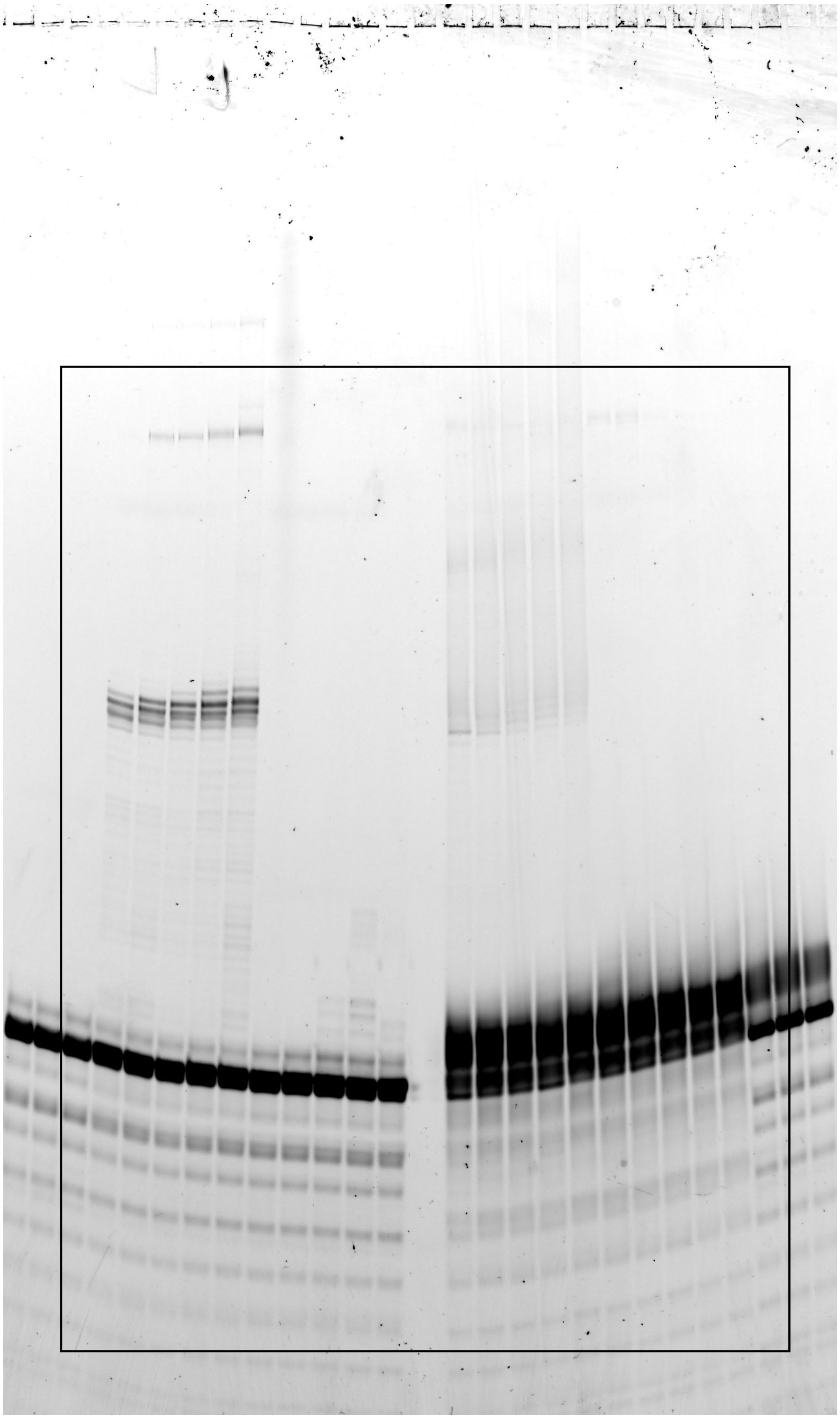

Supplemental Fig. 4

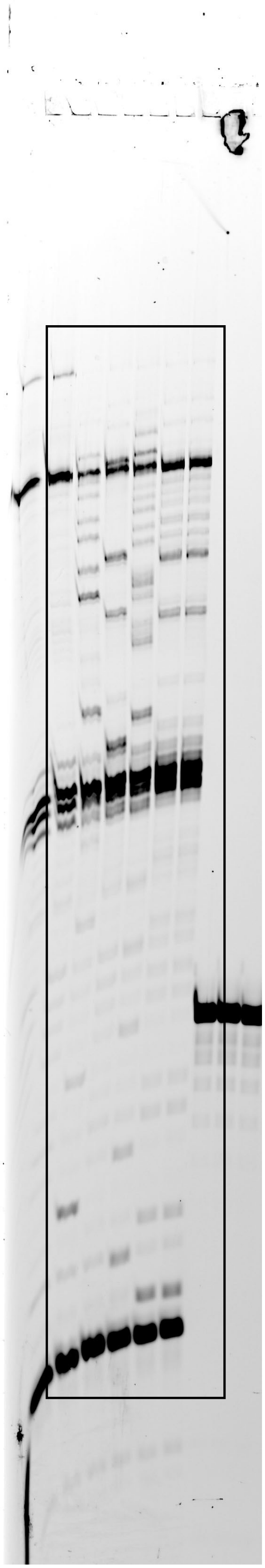

Extended Data Fig. 4a

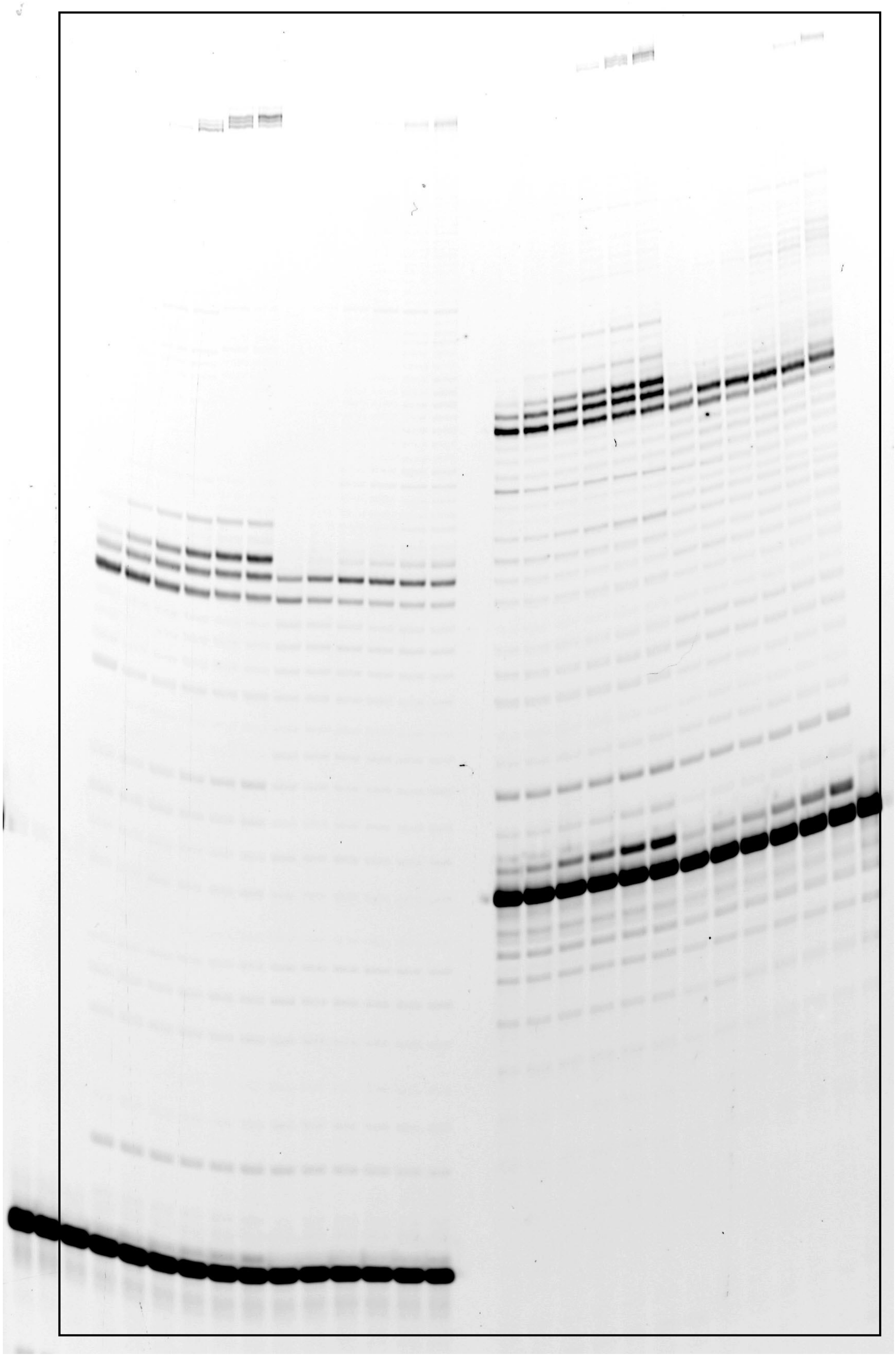

Extended Data Fig. 4b

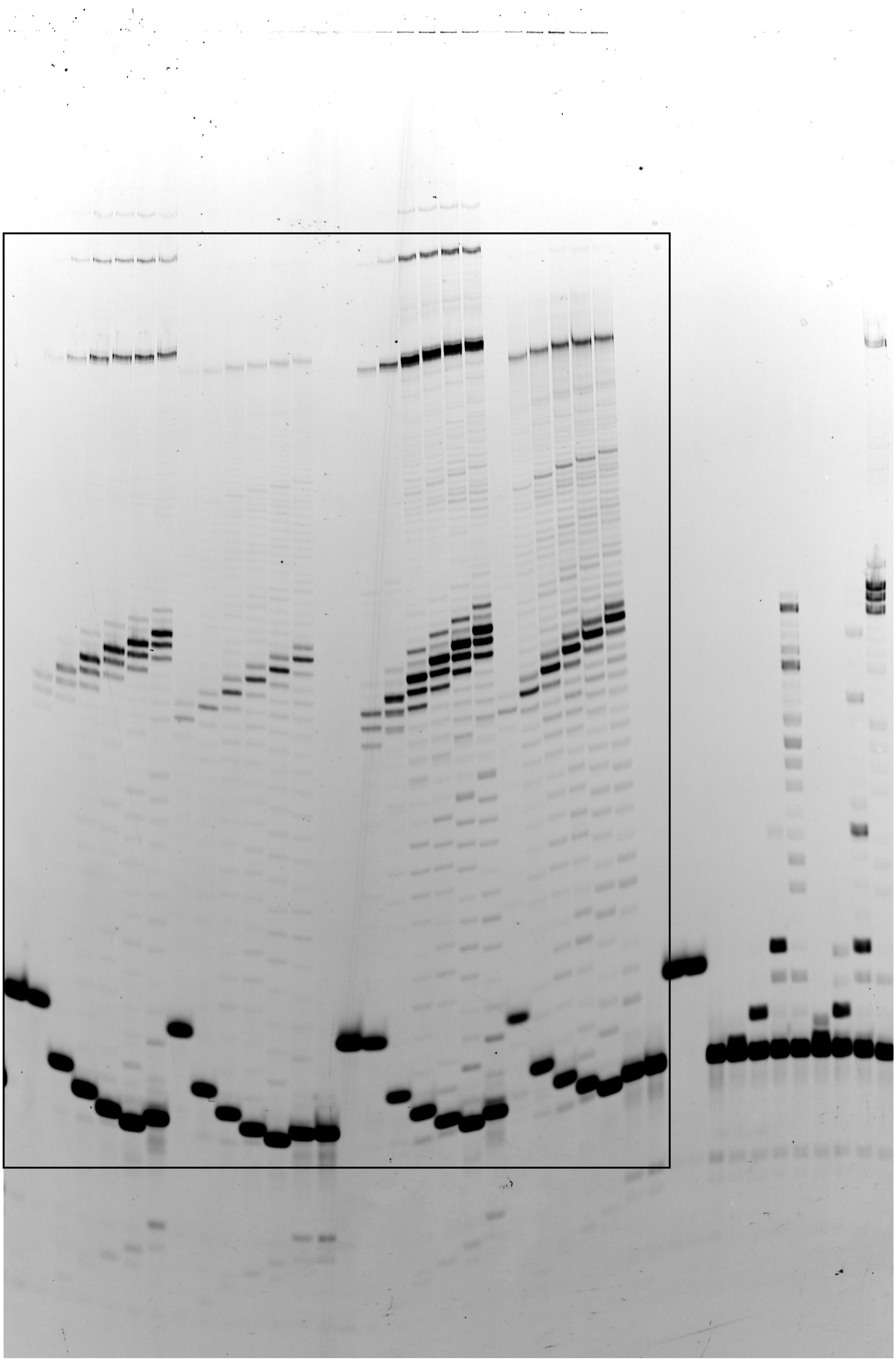

Extended Data Fig. 4c

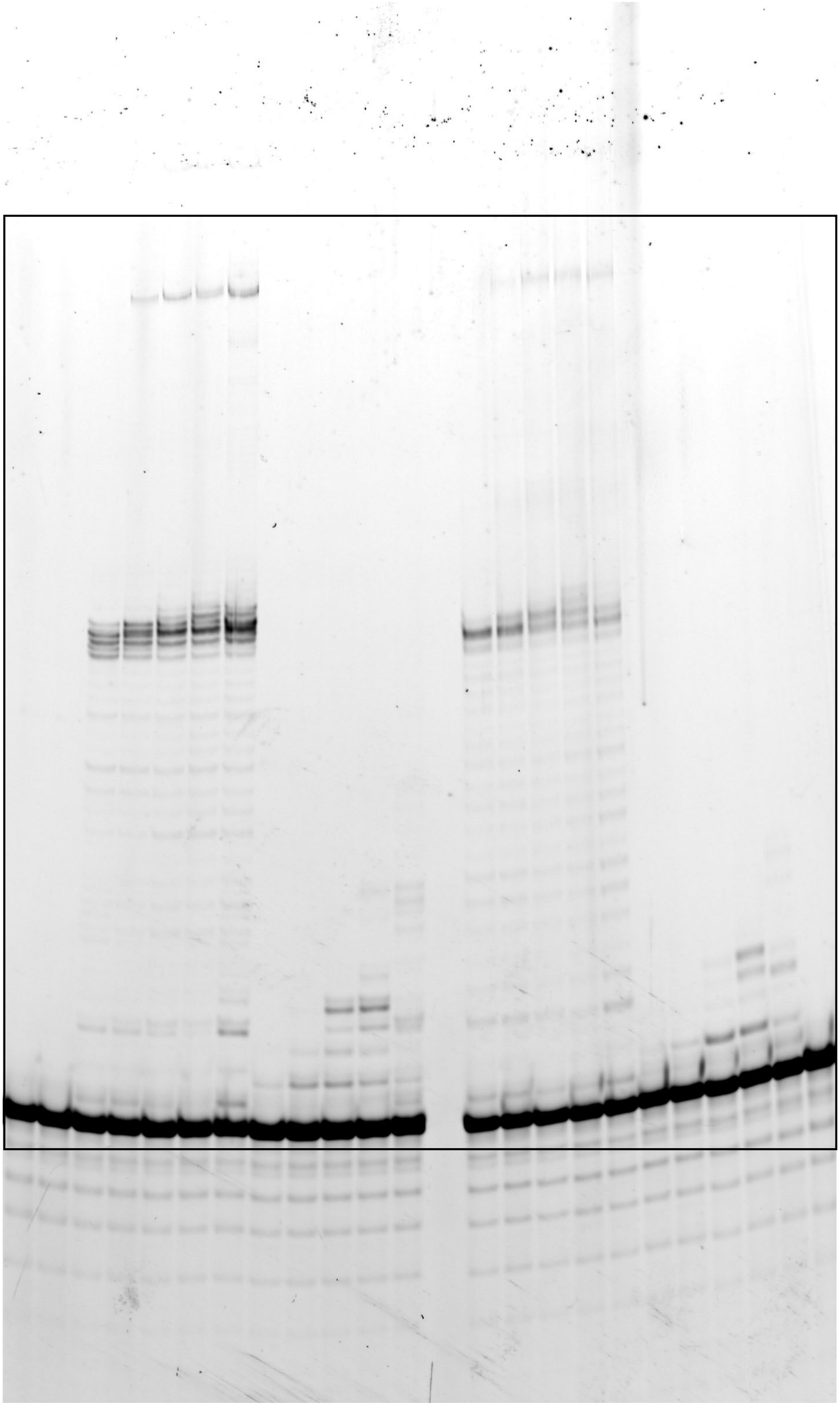

Extended Data Fig. 4d

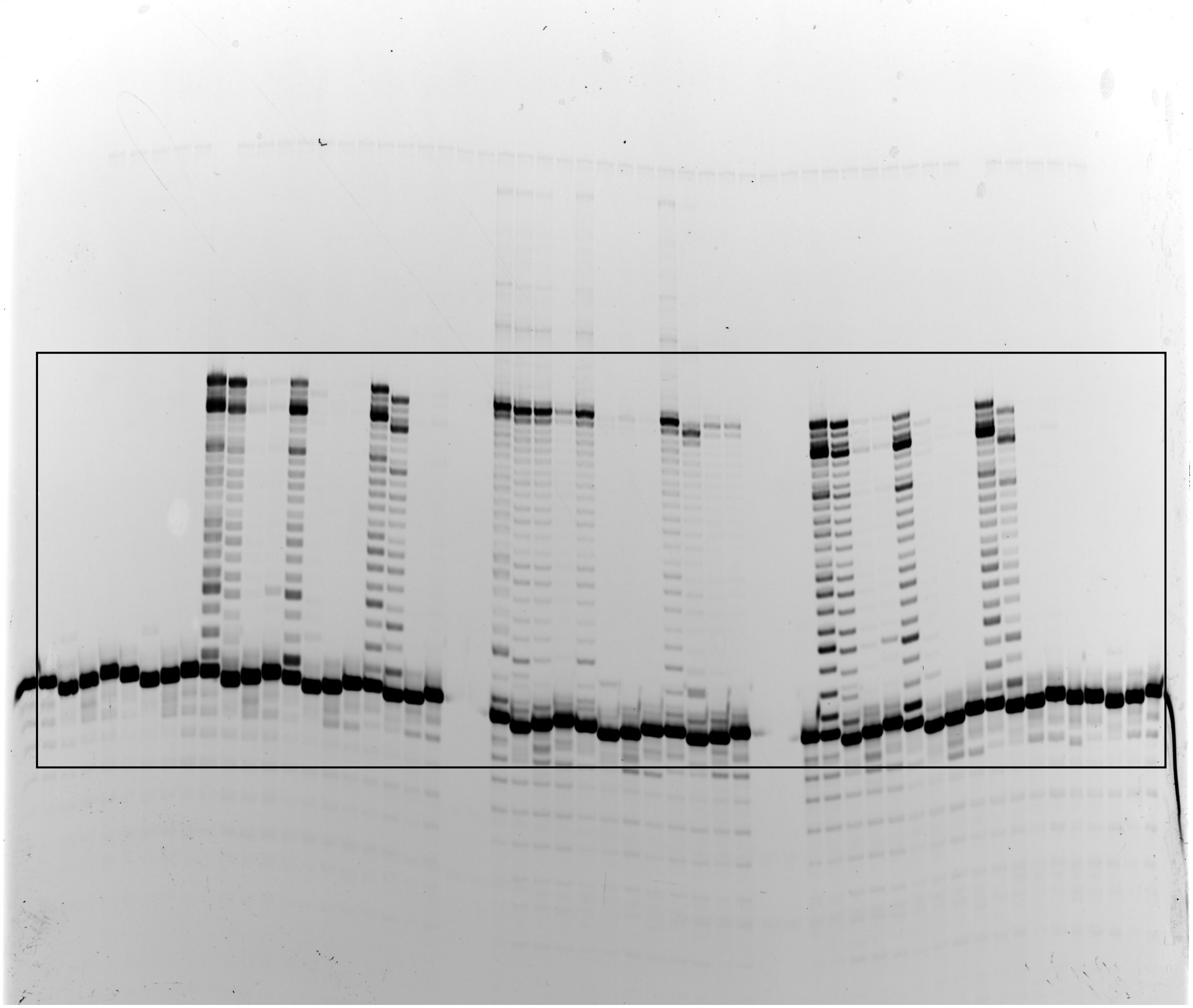

Gel Source Data | Electrophoresis based analyses | E.T. Baldwin et al. *Structures, Functions, and Adaptations of the Human LINE-1 ORF2 Protein*

Fig. 3b / Extended Data Fig. 5a

RNA Template

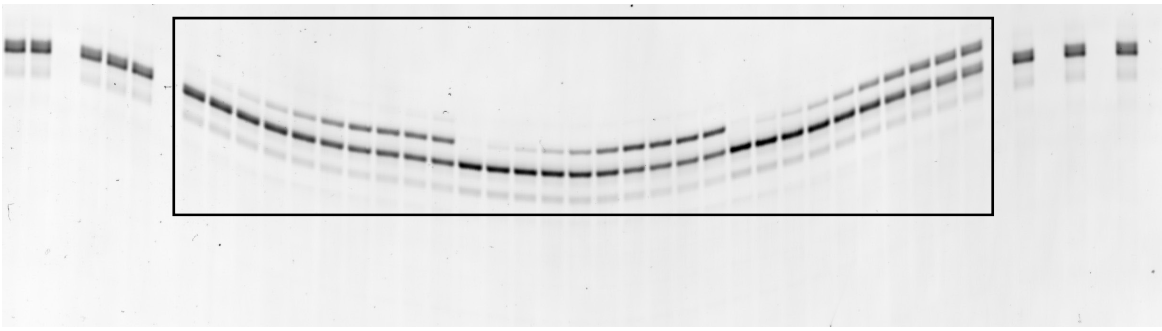

DNA Template

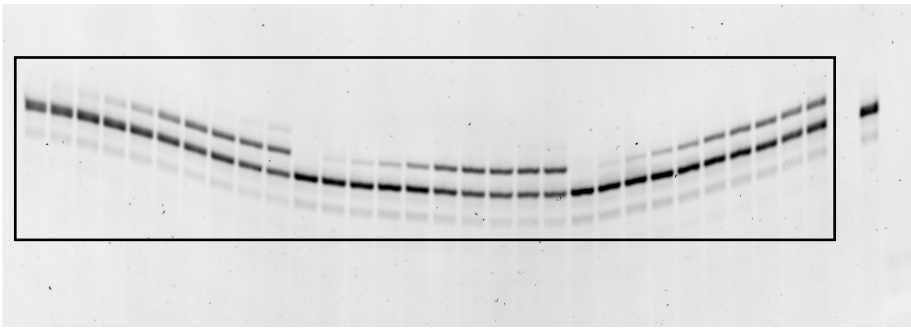

Extended Data Fig. 5B

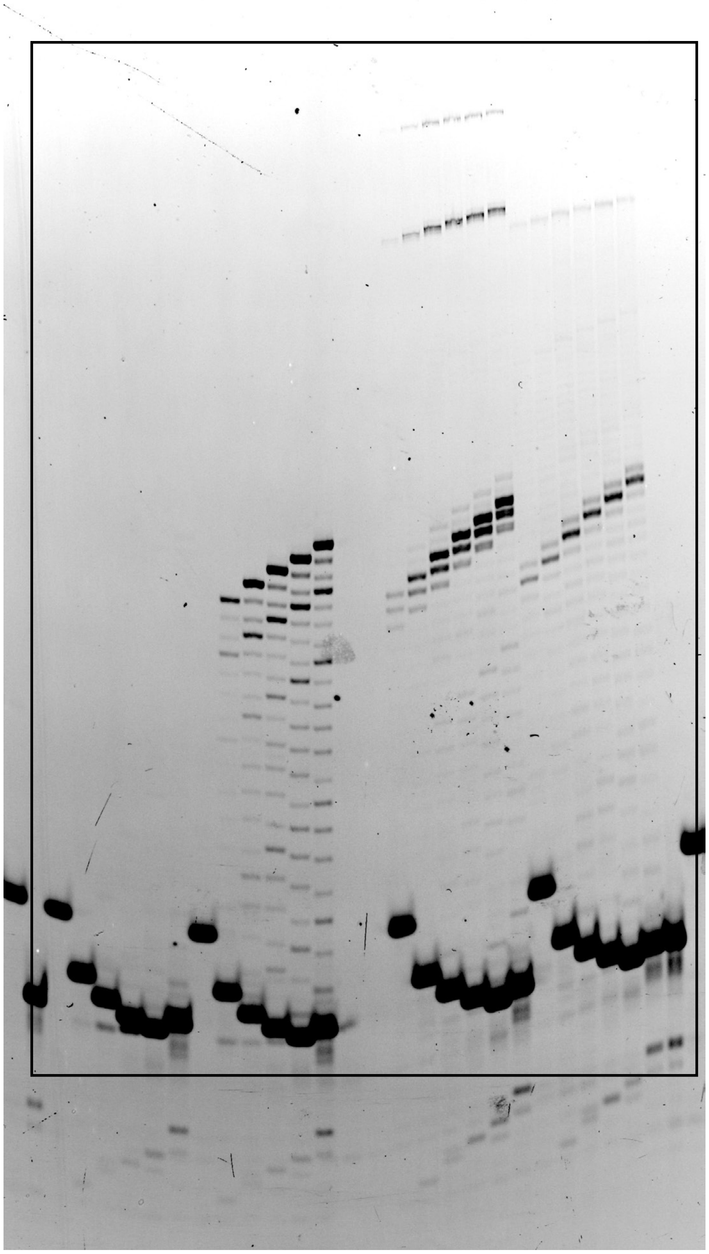

Extended Data Fig. 5c

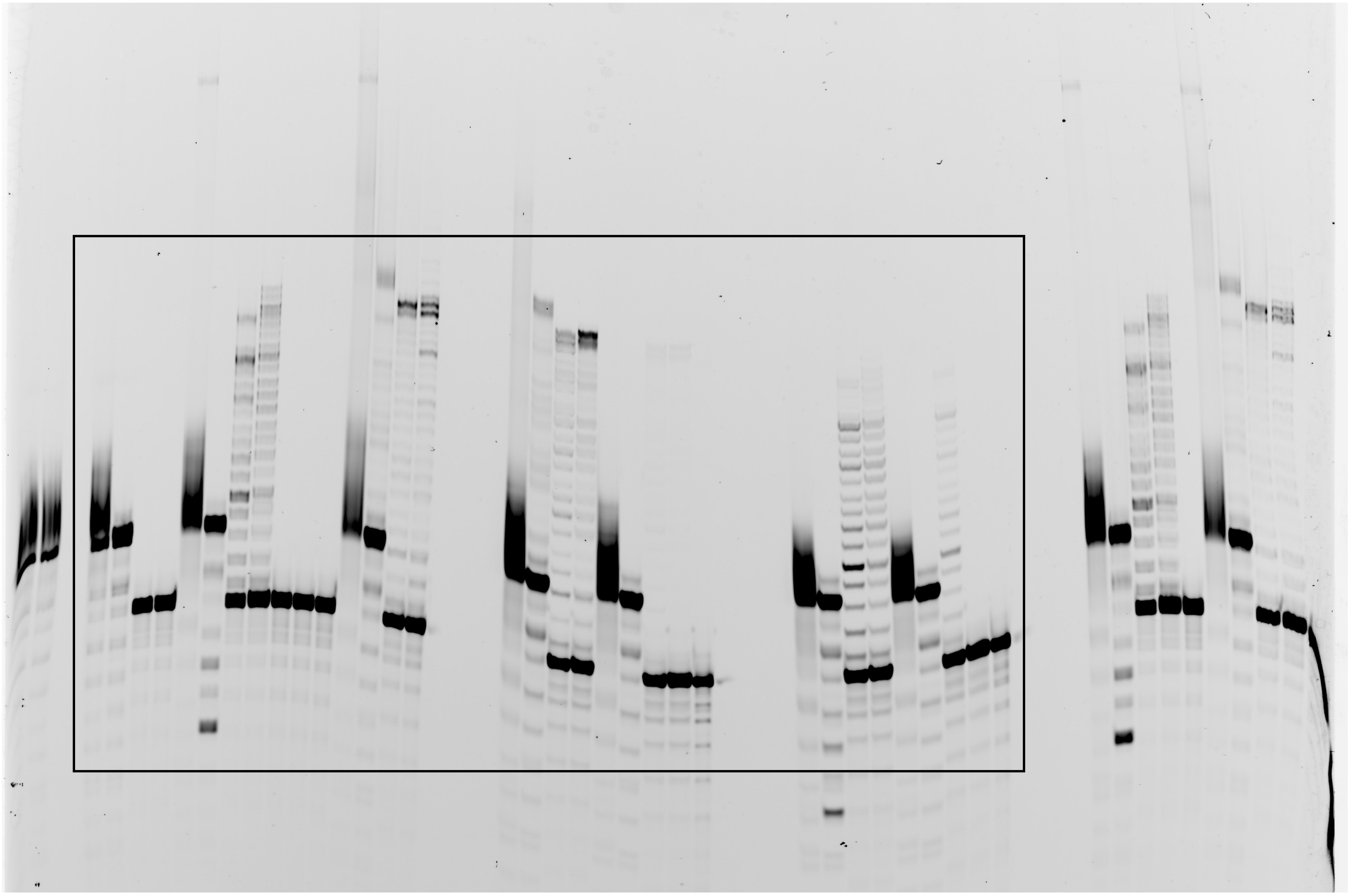

Fig. 3d / Supplemental Fig. 5a

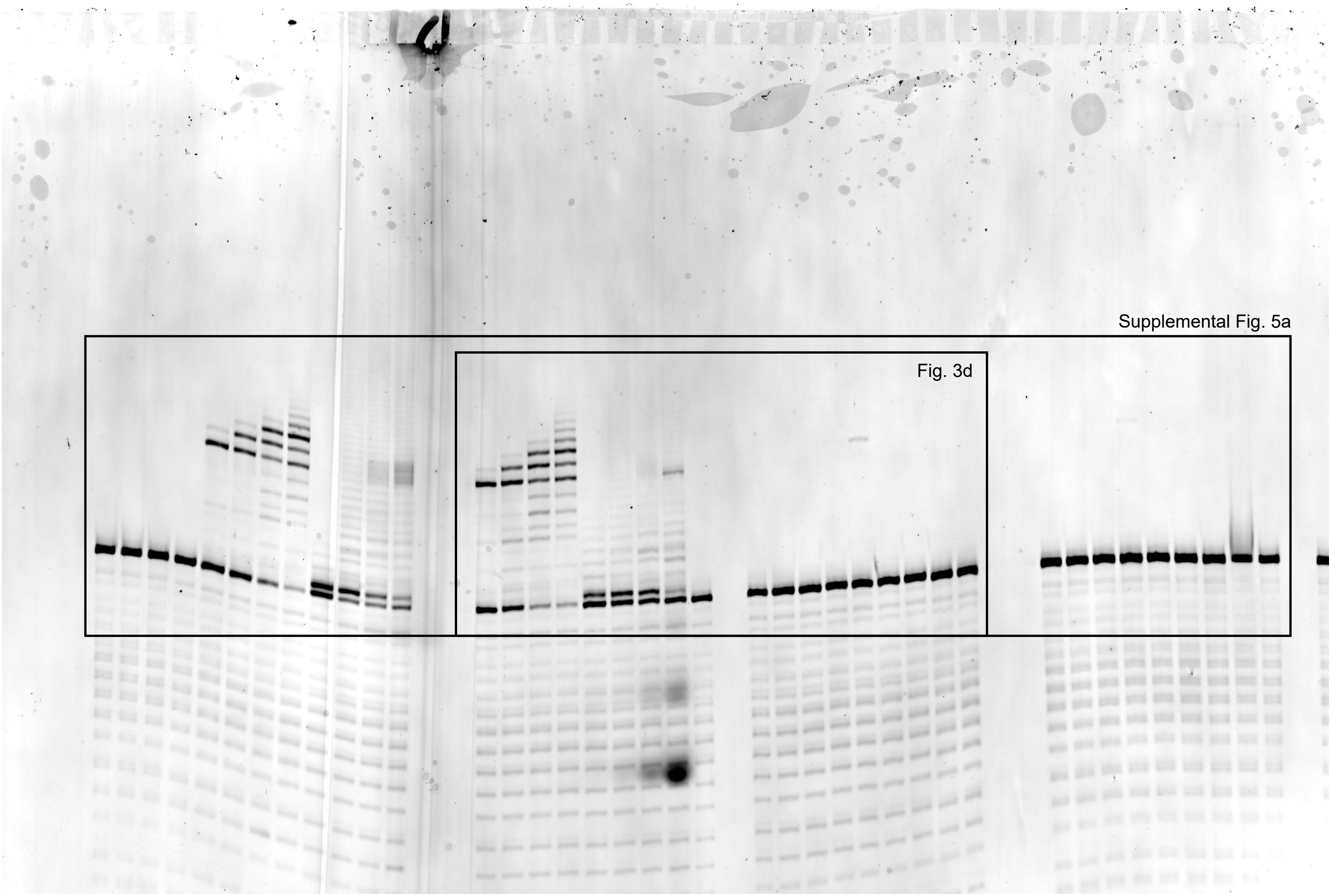

Fig. 3e / Supplemental Fig. 5b

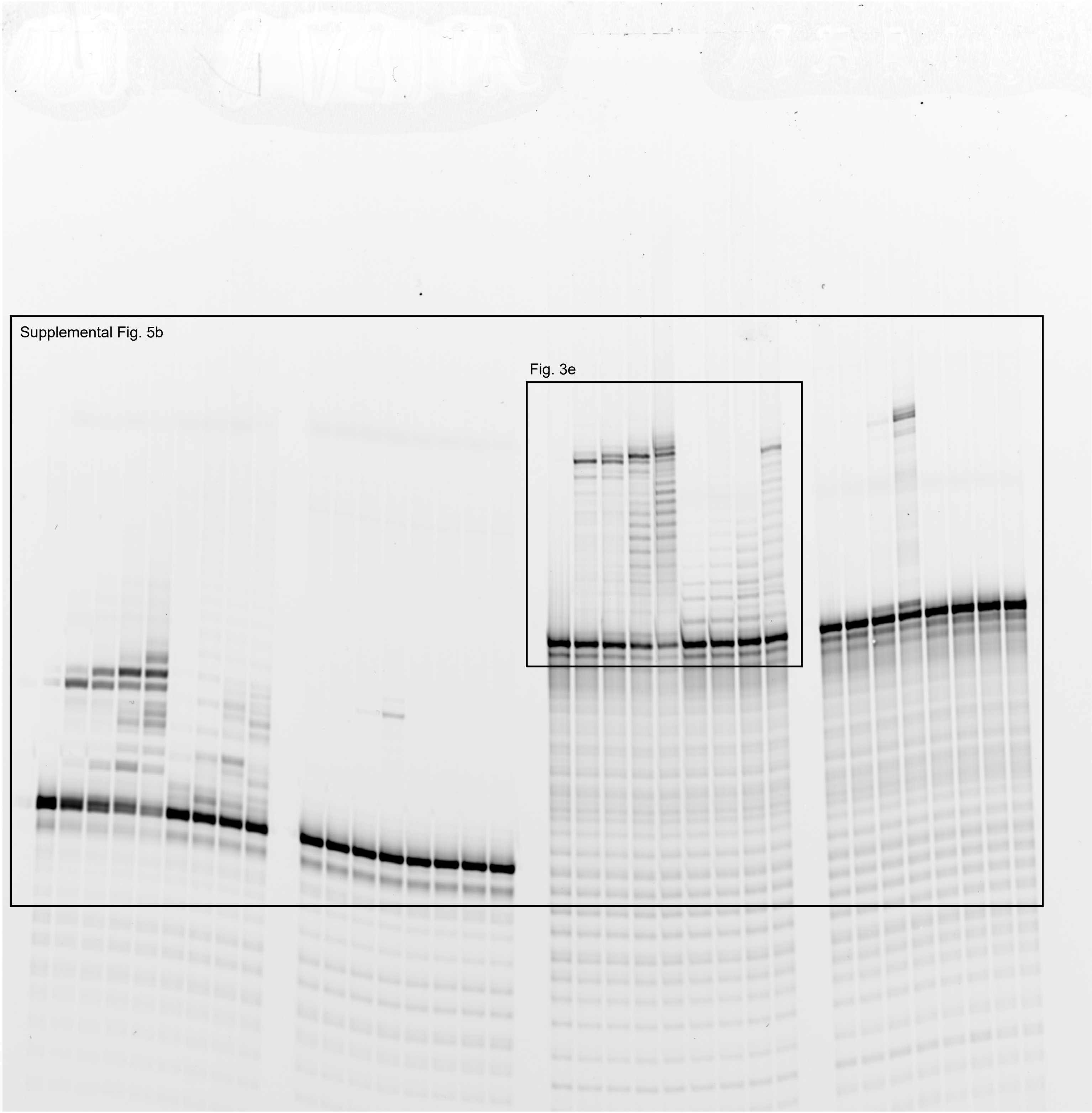

Fig. 3g

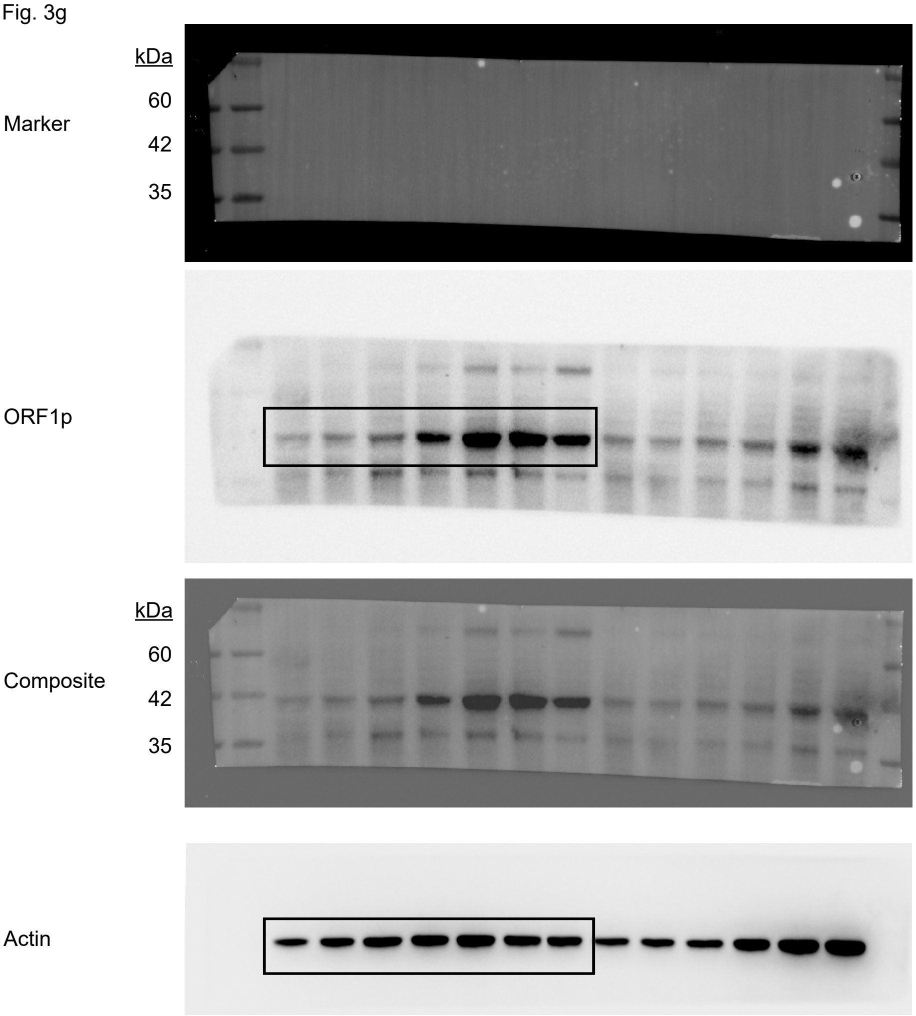

Extended Data Fig. 7a

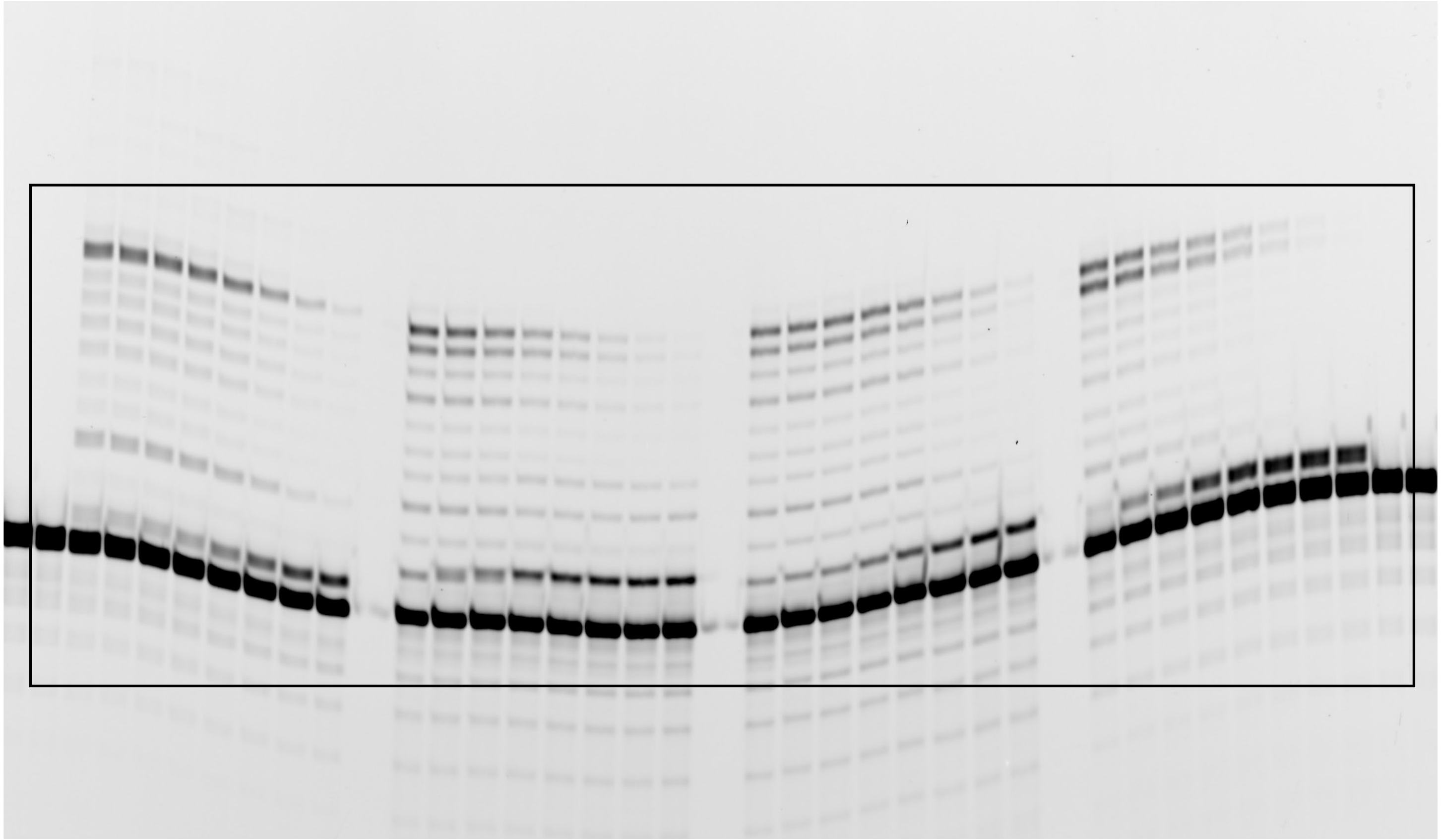

Fig. 4b / Extended Data Fig. 7e

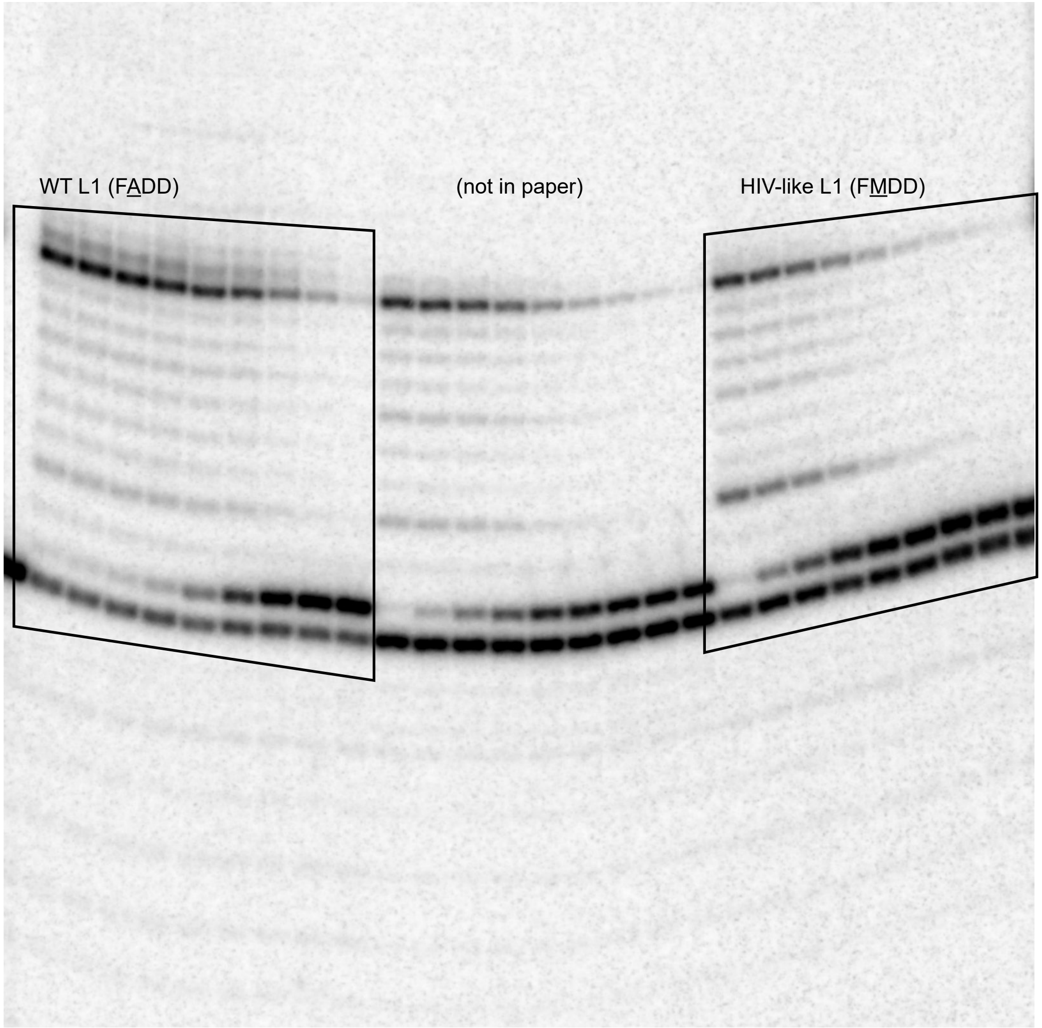

Supplementary Fig. 8c, left panel

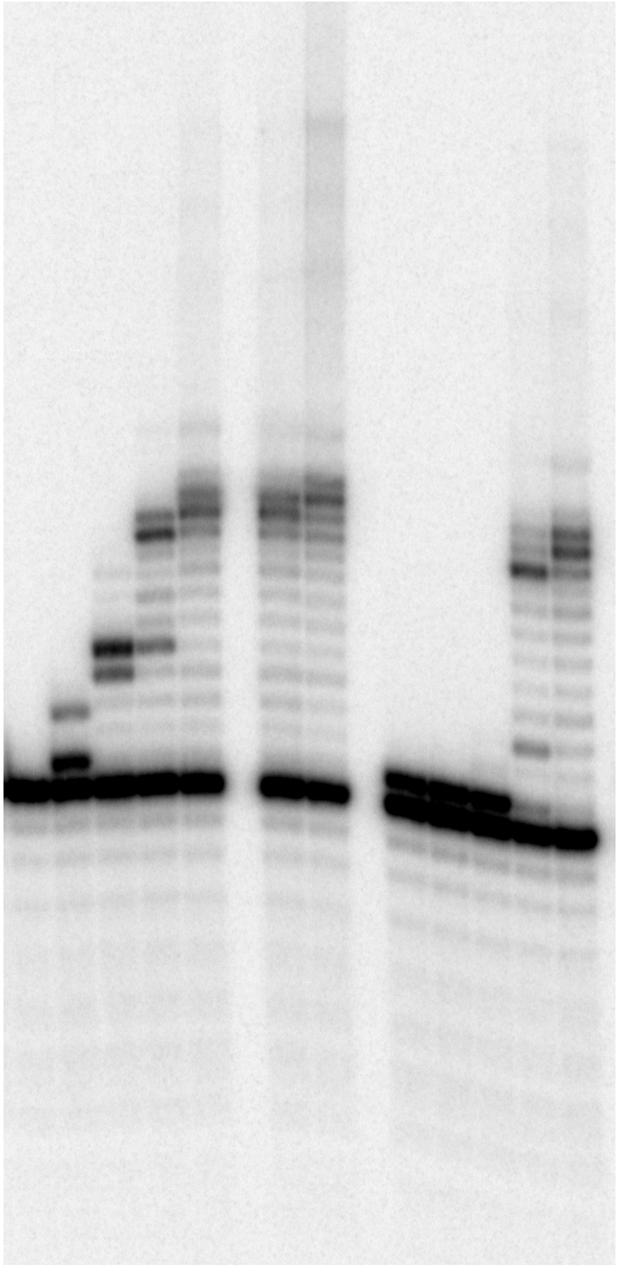

Extended Data Fig. 7f / Supplemental Fig. 8c, right panel

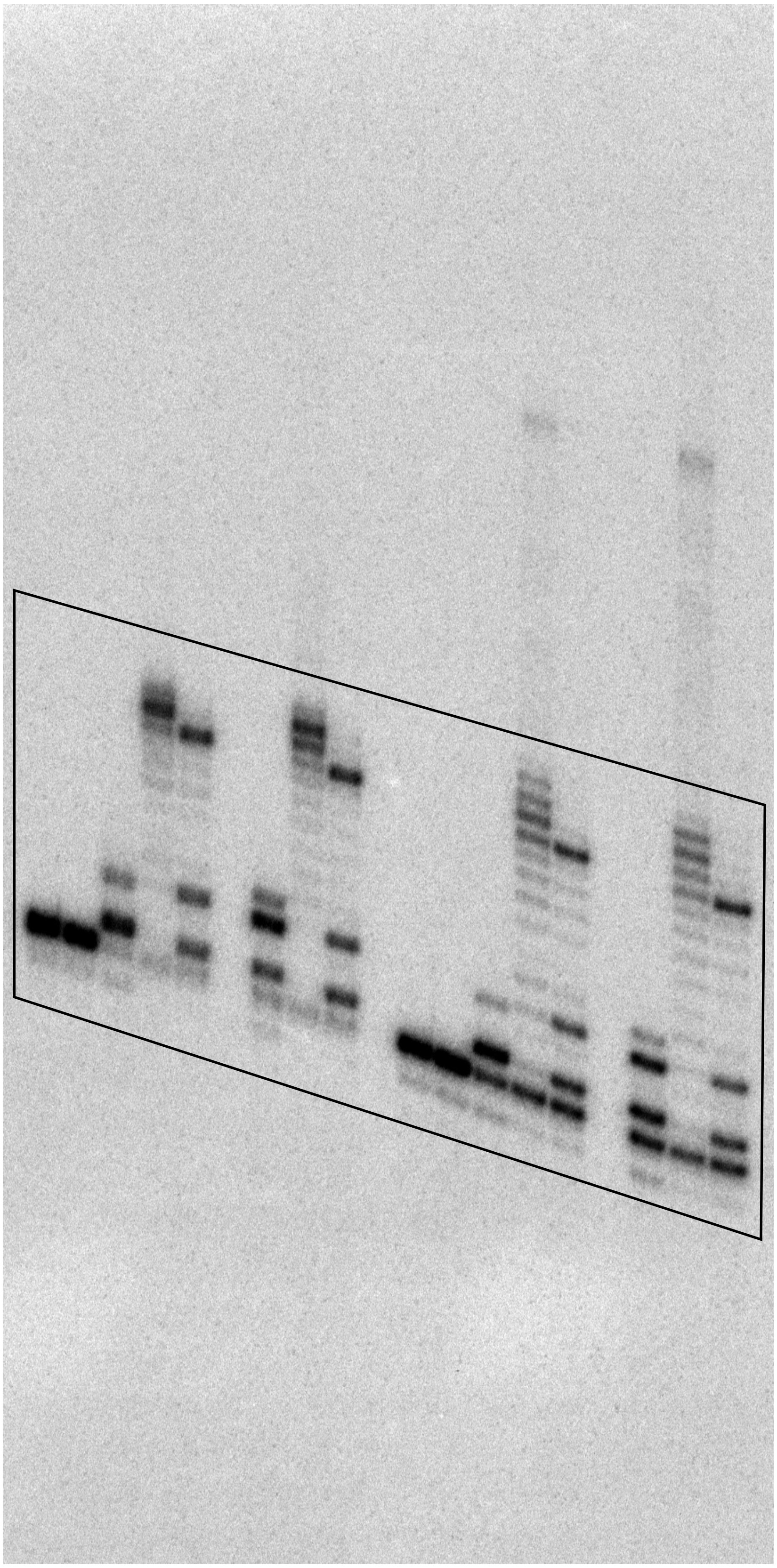

Supplemental Fig. 8b

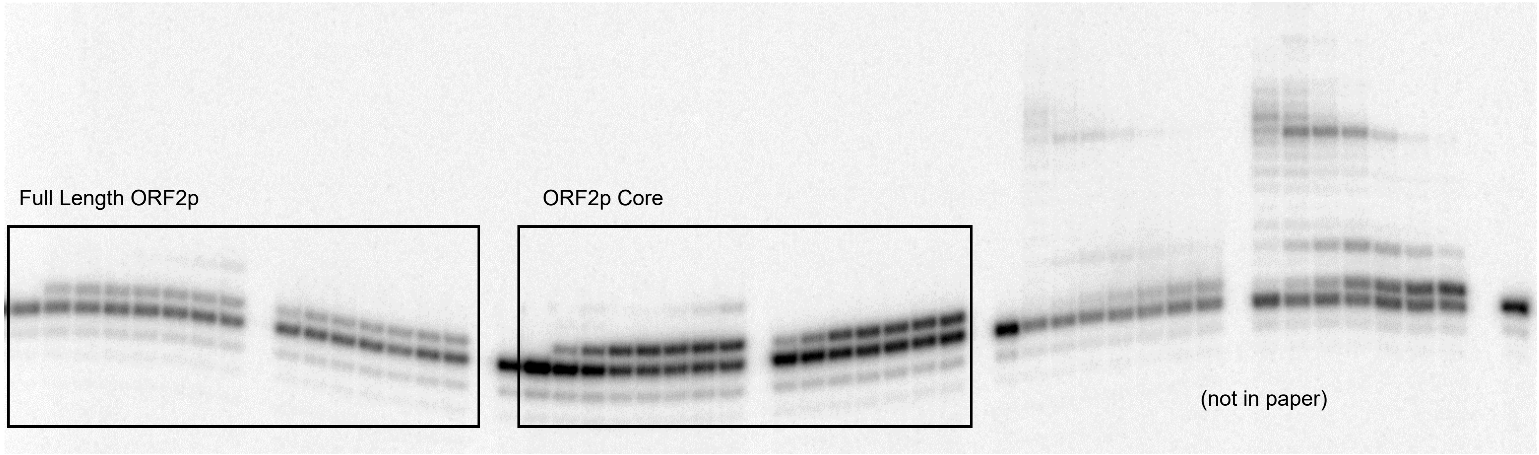

Supplement: Supplementary file 3 — Gel source data. [file 41586_2023_6947_MOESM3_ESM.pdf]
